# Reconfiguration of DNA nanostructures induced by enzymatic ligation treatment

**DOI:** 10.1093/nar/gkac606

**Published:** 2022-07-26

**Authors:** Tanxi Bai, Jiayi Zhang, Kai Huang, Wen Wang, Bowen Chen, Yujie Li, Mengyao Zhao, Suoyu Zhang, Chenyou Zhu, Dongsheng Liu, Bryan Wei

**Affiliations:** School of Life Sciences, Tsinghua University-Peking University Center for Life Sciences, Center for Synthetic and Systems Biology, Tsinghua University, Beijing 100084, China; School of Life Sciences, Tsinghua University-Peking University Center for Life Sciences, Center for Synthetic and Systems Biology, Tsinghua University, Beijing 100084, China; School of Life Sciences, Tsinghua University-Peking University Center for Life Sciences, Center for Synthetic and Systems Biology, Tsinghua University, Beijing 100084, China; School of Life Sciences, Tsinghua University-Peking University Center for Life Sciences, Center for Synthetic and Systems Biology, Tsinghua University, Beijing 100084, China; School of Life Sciences, Tsinghua University-Peking University Center for Life Sciences, Center for Synthetic and Systems Biology, Tsinghua University, Beijing 100084, China; Key Laboratory of Bioorganic Phosphorus Chemistry and Chemical Biology, Department of Chemistry, Tsinghua University, Beijing 100084, China; School of Life Sciences, Tsinghua University-Peking University Center for Life Sciences, Center for Synthetic and Systems Biology, Tsinghua University, Beijing 100084, China; School of Life Sciences, Tsinghua University-Peking University Center for Life Sciences, Center for Synthetic and Systems Biology, Tsinghua University, Beijing 100084, China; Key Laboratory of Bioorganic Phosphorus Chemistry and Chemical Biology, Department of Chemistry, Tsinghua University, Beijing 100084, China; Key Laboratory of Bioorganic Phosphorus Chemistry and Chemical Biology, Department of Chemistry, Tsinghua University, Beijing 100084, China; School of Life Sciences, Tsinghua University-Peking University Center for Life Sciences, Center for Synthetic and Systems Biology, Tsinghua University, Beijing 100084, China

## Abstract

Enzymatic ligation is a popular method in DNA nanotechnology for structural enforcement. When employed as stability switch for chosen components, ligation can be applied to induce DNA nanostructure reconfiguration. In this study, we investigate the reinforcement effect of ligation on addressable DNA nanostructures assembled entirely from short synthetic strands as the basis of structural reconfiguration. A careful calibration of ligation efficiency is performed on structures with programmable nicks. Systematic investigation using comparative agarose gel electrophoresis enables quantitative assessment of enhanced survivability with ligation treatment on a number of unique structures. The solid ligation performance sets up the foundation for the ligation-based structural reconfiguration. With the capability of switching base pairing status between permanent and transient (ON and OFF) by a simple round of enzymatic treatment, ligation induced reconfiguration can be engineered for DNA nanostructures accordingly.

## INTRODUCTION

In the past four decades, the extraordinary power of DNA self-assembly has been showcased in the construction of a myriad of complex DNA nanostructures (1–7). Apart from static structures with increasing complexity and diversity, dynamic constructs driven by strand displacement have also emerged as useful tools in computation and nanomechanics (8–10). With similar design principles, dynamic constructs composed of complex DNA origami units have also been produced (10–15). Moreover, by carefully designing nucleic acids-protein working interface, enzymes have been applied to drive the structural reconfiguration of synthetic DNA constructs (16–18). Enzymatic ligation to seal nicks with phosphodiester bonds is a well-adopted method for the reinforcement of DNA nanostructures (19–24), but has rarely been presented in dynamic DNA constructs (18,25). To utilize the stability difference between the ligated and unligated structural components, we demonstrate DNA nanostructure reconfiguration induced by ligation treatment in this work.

**Figure 1.**
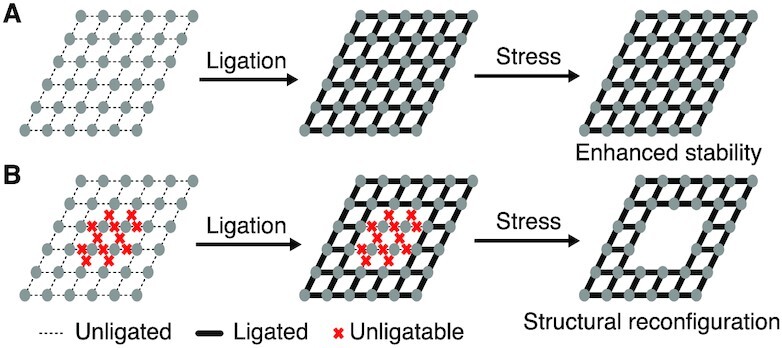
DNA nanostructure stability enhancement and reconfiguration based on enzymatic ligation treatment. (**A**) Schematics of ligation-based stability enhancement. (**B**) Schematics of ligation-induced structural reconfiguration.

The general goal of this work is to utilize the structural stability enhancement from ligation treatment to enable DNA nanostructure reconfiguration (Figure 1). We first hypothesize that a substantial stability enhancement would be achieved with a majority of nicks ligated for a certain addressable DNA nanostructure. Our secondary hypothesis is that a substructure with key segments to be specifically ligated would reconfigure from the original complete structure based on the structural stability difference with and without ligation treatment.

## MATERIALS AND METHODS

### Materials

DNA oligonucleotides were synthesized by Integrated DNA Technology Incorporation or Bioneer Corporation and were used without further purifications. Enzymes were purchased from New England Biolabs (NEB), including T4 polynucleotide kinase (Catalog #M0201L), T4 ligase (Catalog #M0202L), exonuclease III (Catalog #M0206L).

### DNA sequence design

Structures shown in this study were adapted from previous studies (26,27), including three 6 × 6 lattices from 4-arm junction motifs (J4-I, J4-II and J4-III), two 6 × 6 lattices from DX motifs (DX-I and DX-II) and one 6 × 7 lattice from 3-arm junction motifs (J3). The 2 × 2 and 3 × 3 lattices were derived from lattice J4-I with fewer motifs. All the structures were designed by sequence generation software Uniquimer (28).

### Structural assembly

The structures with and without enzymatic ligation treatment followed a typical self-assembly annealing protocol described in previous studies (26,27). The unpurified component strands were mixed roughly equimolar at a final concentration of 100–400 nM in 0.5× TE buffer (5 mM Tris, pH 7.9, 1 mM EDTA) supplemented with 15 mM MgCl_2_ (not subjected to ligation) or 1× T4 DNA ligase buffer (NEB) (subjected to ligation), respectively. The mixtures were annealed under a ‘ramp’ annealing protocol in which samples were denatured at 85 °C for 15 min and then cooled from 85°C to 61°C at 5 min per 1°C and from 60°C to 10°C at 25 min per 1°C over a course of 24 h.

### Ligation treatment

There were two additional steps for the structures with enzymatic ligation treatment, phosphorylation and ligation. (i) Phosphorylation. The unpurified DNA strands of a certain structure were incubated with T4 polynucleotide kinase (PNK) in 1× T4 DNA ligase buffer at 37 °C for 5 h to get 5′ ends phosphorylated before structural assembly since the 5′ phosphate group is not available for a typical synthetic strand. (ii) Ligation. T4 DNA ligase was applied to the post-assembly samples to seal nicks over an incubation at 16 °C for 17 h.

### Agarose gel electrophoresis (AGE)

Samples were subjected to native AGE in an ice-water bath, and gels were prepared in 0.5× TBE buffer (44.5 mM Tris, 44.5 mM boric acid and 1 mM EDTA) supplemented with 10 mM MgCl_2_ and were pre-stained with SYBR Safe (Thermo Fisher Scientific) unless indicated otherwise. AGE results were obtained using a Typhoon scanner (GE Healthcare) equipped with 488 nm laser and Cy2 filter, and a SynGene G:Box F3 gel imager equipped with blue light transilluminator (emission max ∼450 nm) and a UV032 filter (bandpass 572–630 nm).

### Denaturing polyacrylamide gel electrophoresis (PAGE)

Gels contained 10% acrylamide (29:1, acrylamide/bis-acrylamide), 8 M urea and 1× TBE and were run at 50°C unless indicated otherwise. Samples were pre-mixed with equal volume of 2× TBE–urea sample buffer (Sangon Biotech) and denatured at 90°C for 10 min prior to loading. Gels were stained with SYBR Safe and imaged with the Typhoon scanner.

### Thermal denaturation test

Aliquots of self-assembled structures were incubated at temperatures ranging from 30°C to 95°C for 4 h. The products after thermal incubation were directly subjected to 2% native agarose gel. A control sample (stored at 4°C) and 1-kb DNA ladder (Thermo Scientific) were loaded as mobility references.

### Quantitative analysis of assembly yield and melting curves

Gel images were analyzed by ImageJ (version 1.49). To quantify the assembly yield, the intensity of the target band was compared against a standard band (29) (e.g. 1500-bp band from a 1-kb DNA ladder mixture). The mass value of the target band was deduced from the intensity-mass correlation based on the standard band, and was used to calculate the yield of the desired structure. To quantify the survival rate, the intensity of the target band was normalized by that of the control band. Average survival rates and standard deviations were calculated based on three independent AGE results. Melting curves were obtained by SciDAVis (version 0.2.4) and OriginPro (version 2018).

### Atomic force microscopy (AFM) imaging

AFM images were obtained using a SPM Multimode with Nanoscope V controller (Bruker). Normally 50 μl of 0.5× TE buffer with 15–20 mM MgCl_2_ were applied to a freshly cleaved mica surface, and then a 5 μl droplet of a sample (2–10 nM) was added and incubated for ∼2 min. Supplementary 10–20 μl of 10 mM NiCl_2_ was added to increase the strength of DNA-mica binding (30). Additional dilution of the sample was possibly performed to achieve the desired sample density. Samples were imaged under liquid ScanAsyst mode with C-type triangular tips (resonant frequency, ${f_0}$ = 40–75 kHz; spring constant, *k* = 0.24 N m^−1^) from the SNL-10 silicon nitride cantilever chip (Bruker).

## RESULTS

### Ligation-based stability enhancement

To verify our first hypothesis (Figure 1A), we applied ligation treatment on addressable structures entirely from short synthetic strands for stability investigation (26,27). T4 ligase, which catalyzes the sealing of a nick between 3′ hydroxyl group and 5′ phosphate group to form a phosphodiester bond with a high efficiency (>0.9 at 16°C) (31,32), was adopted in our investigations. Because numbers and positions of ligatable nicks are programmable in addressable structures, a careful quantitative analysis of catalytic efficiency leads to more mechanistic insights of ligation reaction. The thermal stability of the ligated structures is characterized by AGE and the corresponding morphologies of the treated samples are confirmed by AFM. Parallel characterization of several unique structures over a broad temperature range points to the general thermal stability enhancement—melting temperatures of ligated structures increase more than 15°C compared to the unligated counterparts.

**Figure 2.**
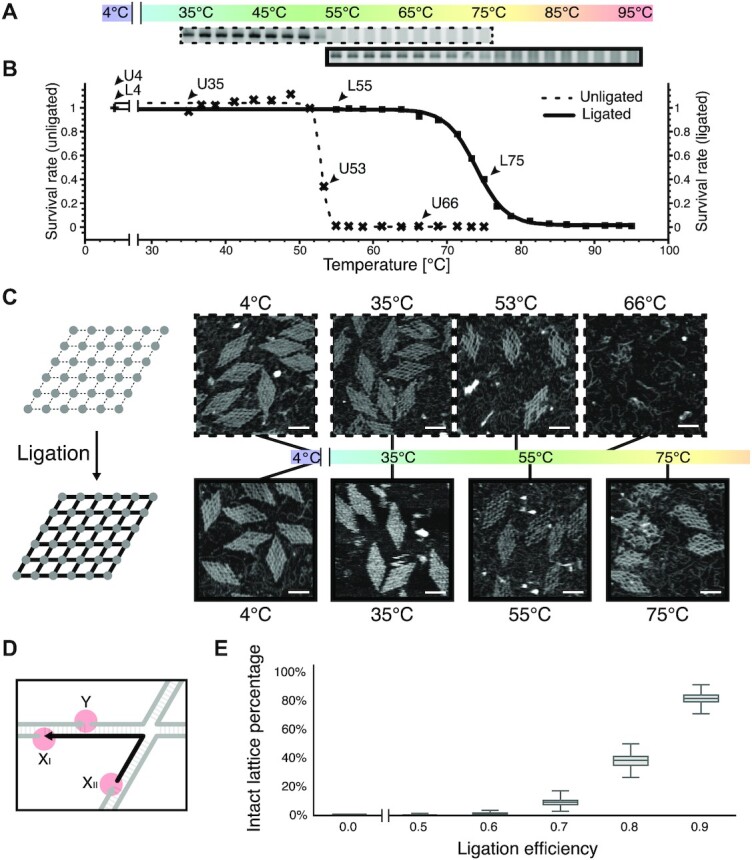
Thermal stability analysis for a 6 × 6 lattice with and without ligation treatment. (**A**) Agarose gel electrophoresis results of unligated (top panels) and ligated (bottom panels) lattices incubated at 35–75°C and 55–95°C respectively. (**B**) Melting curves based on the AGE analysis. Dashed melting curve for the unligated lattice and solid melting curve for the ligated lattice. Arrows point at samples of representative survival rates for AFM imaging. (**C**) Lattice morphologies after thermal denaturation test. AFM images of samples after thermal denaturation test at certain temperatures are highlighted in dashed (unligated) and solid (ligated) boxes. Scale bars: 50 nm. (**D**) Schematics of representative ligation events to lock in a certain component strand (highlighted in black). Full lock-in effect rendered for ligation events at nicks X_I_ or X_II_ and partial effect for that at nick Y. (**E**) Simulation results of intact lattice proportion survived at an elevated temperature as a function of gradient levels of ligation efficiency.

We first implemented a thermal denaturation test to study the thermal stability of DNA nanostructures with and without ligation treatment (Supplementary Figures S1–S15). The samples were incubated in gradient temperatures ranging from 30°C to 95°C (Supplementary Table S1) and then directly subjected to AGE. The intensities of the target bands were assessed and normalized by the control band from an untreated sample. The normalized yields were defined as the survival rates at specific temperatures (Supplementary Figure S9). The average survival rate of triplicates at a certain temperature was plotted as a function of temperature, and a melting curve was obtained by fitting the dataset to a Boltzmann sigmoid. The specific incubation temperature corresponding to a survival rate of 0.5 was defined as the melting temperature (*T*_m_) of the structure. We took a 6 × 6 lattice assembled from 36 4-arm junction motifs (J4-I) as a case study (Figure 2; Supplementary Figures S3 and S10). Each arm of the motif has an 11-bp root domain (double-stranded) and a 10-nt stem domain (single-stranded) with a typical edge resulting from base pairing of two complementary stem domains appended to the respective root domains (Supplementary Figures S1 and S2). There are 120 nicks in total for the entire lattice of 60 edges. According to our AGE analysis, the *T*_m_ of the ligated lattice increased to 74°C compared to the *T*_m_ of 53°C for the unligated control (Figure 2B; Supplementary Figure S3; Supplementary Table S2), which clearly shows a substantial enhancement of thermal stability (Δ*T*_m_ > 20°C). Sample points of representative survival rates (1, 0.4, 0) were collected for unligated controls (4°C, 35°C, 53°C and 66°C) and ligated samples (4°C, 35°C, 55°C, 75°C and 89°C) for AFM imaging respectively (Figure 2C; Supplementary Figure S10). Handsomely corroborating our AGE analysis, AFM results showed intact morphologies below *T*_m_ and a deteriorating integrity around and above *T*_m_.

To calibrate the efficiency of enzymatic ligation on DNA nanostructures, we took a 2 × 2 lattice of 4-arm junction motifs in our specific investigation (Supplementary Figures S16–S20). Since the non-circular ligation products were generated regardless of the proper self-assembly of the designated structures, we focused on the circular product for the yield analysis. According to our careful analysis and calculation based on denaturing PAGE, the ligation efficiency was estimated at 0.89 (Supplementary Figure S20; see the Supplementary Information for details of the calculations). Another 3 × 3 lattice from 4-arm junction motifs was designed for the ligation performance investigation (Supplementary Figures S21–S26). The pattern of the circular ligated products on AGE was in nice agreement with the theoretical estimation based on the calibrated ligation efficiency (Supplementary Figure S26).

The thermal stability enhancement of the ligated DNA nanostructures could be explained by the Kinetically Interlocking Multi-Unit effect (33,34). After ligation treatment, the short pairing domains would get buried in the interlocked longer ligated pairing domains and their dissociation would become less likely due to the unfavorable kinetics (higher activation energy). As our simulation results also revealed, an elevated temperature above the melting temperature of a root/stem domain (10/11 bp) but below that of the combined segment of a root domain and a stem domain (21 bp) would not affect the general integrity of the ligated structure with a modest ligation efficiency (Figure 2D and E; Supplementary Figures S27–S29). Notably, the simulated proportion of intact lattice shown in Figure 2E might be an underestimation since some dissociation events could be transient.

**Figure 3.**
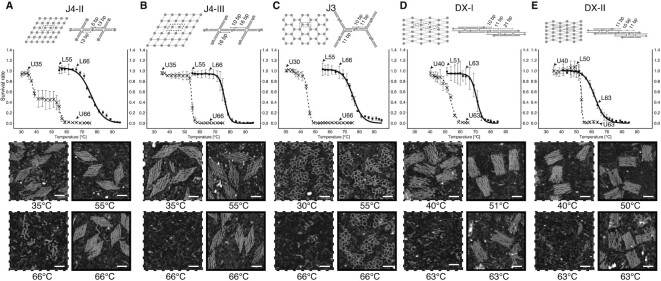
Thermal denaturation test for five lattice species with and without ligation treatment. (A and B) 6 × 6 lattices from 4-arm junction motifs. 13-bp root domains/6-bp stem domains for J4-II (**A**), and 16-bp root domains/10-bp stem domains for J4-III (**B**). (**C**) 6 × 7 lattice from 3-arm junction motifs. (D and E) 6 × 6 lattices from DX motifs. 21-bp rigidity domains/10-bp sticky domains for DX-I (**D**) and 11-bp rigidity domains/11-bp sticky domains for DX-II (**E**). Top panels: schematics of unligated lattices and dashed boxes highlight component motifs with strand level of details. Middle panels: melting curves of unligated (dashed lines) and ligated (solid lines) lattices. Double-Boltzmann sigmoidal fitting for unligated lattice J4-II (dashed curve in A) and Boltzmann sigmoidal fitting for the other lattices. Arrows point at samples incubated at specific temperatures for AFM imaging. Bottom panels: AFM images of samples after thermal denaturation test at chosen temperatures are shown in dashed (unligated) and solid (ligated) boxes. Scale bars: 50 nm.

Because of the streamlined procedures of enzymatic ligation treatment and parallel characterization for the unligated and ligated structures, we then managed to investigate the thermal stability enhancement of ligation treatment for five other lattice species (Figure 3) to show the general stabilizing effect on different types of DNA nanostructures. Another two 6 × 6 wireframe lattices from 4-arm junction motifs (13-bp root domains/6-bp stem domains for J4-II and 16-bp root domains/10-bp stem domains for J4-III) were tested (Figure 3A and B). Besides, one 6 × 7 wireframe lattice from 3-arm junction motifs (11-bp root domains/10-bp stem domains) (Figure 3C) and two 6 × 6 lattices from DX motifs (21-bp rigidity domains/10-bp sticky domains for DX-I and 11-bp rigidity domains/11-bp sticky domains for DX-II) (Figure 3D and E) were also prepared for ligation treatment and thermal denaturation test. There are 120, 120, 108, 146 and 110 ligatable nicks for lattices J4-II, J4-III, J3, DX-I and DX-II respectively. According to the melting curve shown in Figure 3A, a two-stage melting was presented for the unligated lattice J4-II. Presumably, the denaturing of the 6-bp stem domains corresponds to the first stage melting and the denaturing of the 13-bp root domains to the second stage. Despite the similarity between lattices J4-II and J4-III, the length difference between the root and stem domains for the unligated lattice J4-III was not as significant, and a two-stage melting was not presented. In all six pairs of melting curves (unligated and ligated), we observed a significant *T*_m_ increment after ligation (Δ*T*_m_ of 9–38°C) (Figure 3, middle panels; Supplementary Figures S4–S8; Supplementary Table S2). AFM images of samples at chosen incubation temperatures also confirmed the enhanced thermal stability from ligation treatment (Figure 3, bottom panels; Supplementary Figures S11–S15). Likewise, substantial enhancement of thermal stability was observed in DNA origami structures with ligatable nicks (results not shown). On the contrary, another addressable wireframe lattice without ligatable nicks showed no significant enhancement of thermal stability after ligation treatment (Supplementary Figure S30).

The AGE result did not necessarily reveal the mechanical fragility of a certain structure (26). For example, although gel bands of unligated lattice J4-II appeared similar to the ligated counterparts below *T*_m_, our AFM results revealed that the unligated lattices with 6-bp stem domains were mechanically fragile upon deposition on mica surface. Instead of complete lattices with all the component motifs, only partial lattices alongside with broken-off individual motifs were observed below *T*_m_ (Figure 3A). That was also the case for the sample without ligation treatment stored at 4°C (Supplementary Figure S31A). On the other hand, samples after ligation treatment gained improved mechanical stability and the majority of structures shown under AFM were complete lattices below *T*_m_.

### Ligation-induced structural reconfiguration

Having the first hypothesis of substantial enhancement of structural stability verified, we continued on for the second hypothesis to implement ligation treatment on constructs with both ligatable and unligatable segments for dynamic structural reconfiguration. A thermal stability difference can be created between ligated segments and unligated ones within a certain DNA nanostructure. In other words, when the chosen edges were designed as ligatable and others as unligatable, the ligation followed by heating treatment resulted in reconfiguration of the complete lattice into a desired shape, presenting the switch of base pairing status (permanent versus transient) from ON to OFF. Furthermore, when the switch of base pairing status (transient versus permanent) was implemented from OFF to ON, reconfiguration directly responsive to ligation treatment was accomplished. In general, both thermal and mechanical stability of the ligated structures were significantly enhanced, and site specific ligation treatment on addressable DNA nanostructures resulted in dynamic DNA self-assembly.

**Figure 4.**
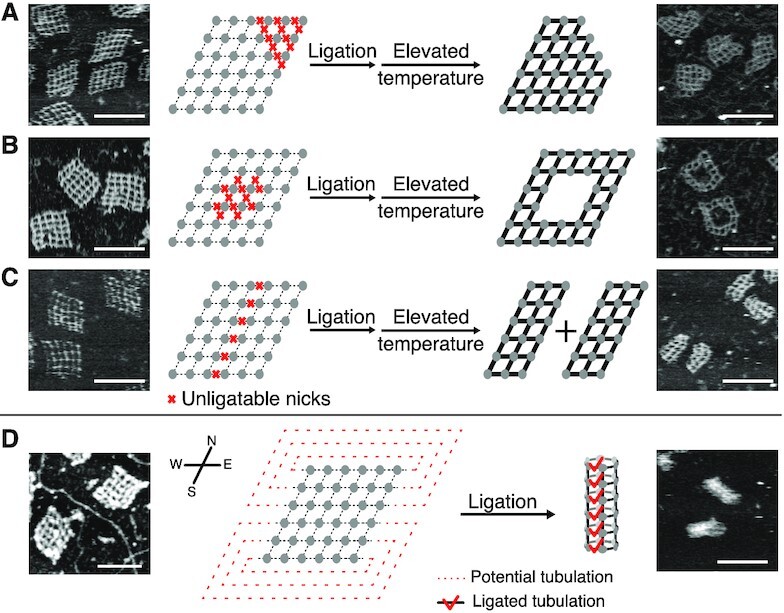
Dynamic structural reconfigurations of a 6 × 6 lattice by enzymatic ligation. (**A–C**) Dynamic structural reconfigurations by loss of ligation function. The dissociation of edges marked with red crosses resulted in three respective patterns. (**D**) Dynamic structural reconfigurations by gain of ligation function. The potential of tubulation (marked by dashed lines in red) is realized by ligation (marked by red ticks). AFM images (leftmost and rightmost) are shown side by side with the respective substrates and products of ligation-related treatment. Scale bars: 100 nm.

As such, a lattice adapted from J4-I was used for the investigation (Figure 4A–C; Supplementary Figures S32–S35). Taking advantage of programmability and addressability of such a DNA construct, we applied phosphorylation (addition of 5′-phosphate groups by T4 polynucleotide kinase) on the selective component strands for them to be ligated in successive ligation treatment. Three different patterns were implemented on this particular construct with 96, 96 and 108 ligatable nicks (out of 120) respectively (Figure 4A–C, left panels). After ligation treatment, the thermal stability enhancement was established only for the ligatable segments but not the unligatable ones. The following heating treatment above 50°C dissociated unligatable segments and resulted in structural reconfiguration into desired patterns from the original 6 × 6 lattice (Figure 4A–C, right panels).

The carving out of certain sub-lattices from the complete lattice by dissociation of certain unligated sites can be viewed as reconfiguration engineered from the loss of ligation function. We then sought to realize structural dynamics based on the gain of ligation function (Figure 4D; Supplementary Figures S36–S38). In another lattice adapted from J4-I, the individual 3-nt overhangs on the east and west sides of the lattice were designed as complementary respectively. Because of the weak base pairing, such a structure remained as a planar sheet after self-assembly (Figure 4D, left). Upon ligation treatment, the transient binding of the sticky ends became efficiently locked in, which collectively transformed the planar lattice into a tubular shape (Figure 4D, right). As negative controls, when the side overhangs were intentionally prepared as non-complementary or unligatable, a planar configuration remained after ligation treatment. Our positive controls of 10-bp sticky end side overhangs with and without ligation pointed to the similar tubular configuration in AGE and AFM results. The results of negative and positive controls further validated the induction role of ligation treatment on the sheet-to-tube reconfiguration (Supplementary Figures S37 and S38).

## DISCUSSION

Enzymatic ligation is already a proven method to enhance thermal and mechanical stability of DNA nanostructures (19–24). In this study, addressable structures with programmable nicks serve as a desirable system to better investigate ligation performance quantitatively. Multiple addressable structures entirely from synthetic strands tested under a series of environmental conditions are characterized by AGE in parallel. Most of the earlier investigations of enzymatic ligation were based on structures of compact helices. According to the conventional wisdom, the accessibility issue for ligase to get a proper hold on the nicking sites to form the phosphodiester bonds could lead to poor ligation efficiency. The porous type of structures of wireframe architecture provides a more workable interface with other biomolecules such as enzymes. For example, the steric hindrance would be less of an issue for enzymatic ligation treatment. In our investigations of various types of structures, difference of ligation efficiency between compact structures and wireframe structures is not significant (*T*_m_ of different lattice species with ligation are similar; Supplementary Table S2). Gaps between compact helices due to the electrostatic repulsion could presumably provide access of ligase to seal nicks of the DNA nanostructures (35).

Because of the collective enforcement from the ligated segments, the base pairing behavior and the enhanced structural stability of the chosen sub-lattice can be adjusted by ligation treatment. Such a reliable base pairing status switch based on collective ligation enforcement is believed to be the enabler of our DNA nanostructure dynamic reconfiguration. According to our simulation, an enhanced structural stability is achievable even in a non-optimized ligation efficiency (e.g. >0.7). Such a finding helps us better understand the robust performance of stability enhancement and dynamic reconfiguration by ligation treatment.

The reconfigured shape after ligation can be viewed as the desired output masked by the original one, which indicates a potential for our method to be used in biomolecular encryption (36–38). Ligation patterns are stochastic among different lattice copies. When the number of sealed nicks of a lattice copy reaches a certain threshold, the interlocked rings can percolate through the entire structure as an interlocked supermolecule with a chain mail topology. Percolated supermolecules can survive in stringent conditions and be perfectly resistant to a number of DNA-digesting enzymes. This special topology led to resistance to all kinds of extreme denaturing conditions (e.g. 90°C incubation, 70% formamide and 8 M urea) and exonuclease digestion (Supplementary Figure S24). The resulting constructs could then enable broad applications in material science and biology research (39–41).

## DATA AVAILABILITY

All data needed to evaluate the conclusions in the paper are present in the paper and/or Supplementary Information.

## Supplementary Material

gkac606_Supplemental_File

## Notes

Present address: Kai Huang, Technology and Process Development, WuXi Biologics, 288 Fute Zhong Road, Waigaoqiao Free Trade Zone, Shanghai 200131, China.

Present address: Wen Wang, BGI-Shenzhen, George Church Institute of Regenesis, Shenzhen 518083, China.

Present address: Suoyu Zhang, Department of Head and Neck Surgery, Sun Yat-Sen University Cancer Center, Guangzhou 510060, China.
