# Supplementary material for: Reconfiguration of DNA nanostructures induced by enzymatic ligation treatment
Source: Nucleic Acids Res. 2022 Jul 26;50(14):8392–8. doi: 10.1093/nar/gkac606 (PMC9371897; doi:10.1093/nar/gkac606)
Supplement: gkac606_Supplemental_File [file gkac606_supplemental_file.pdf]

Supplementary Information

Reconfiguration of DNA nanostructures induced by enzymatic ligation treatment

Tanxi Bai[1,†], Jiayi Zhang[1,†], Kai Huang[1], Wen Wang[1], Bowen Chen[1], Yujie Li[2], Mengyao Zhao[1], Suoyu Zhang[1], Chenyou Zhu[2], Dongsheng Liu[2,*], Bryan Wei[1,*]

[1]School of Life Sciences, Tsinghua University-Peking University Center for Life Sciences, Center for Synthetic and Systems Biology, Tsinghua University, Beijing 100084, China.
[2]Key Laboratory of Bioorganic Phosphorus Chemistry and Chemical Biology, Department of Chemistry, Tsinghua University, Beijing 100084, China
[†]These authors contributed equally.
*Correspondences and requests for materials should be addressed to B.W. (email: bw@tsinghua.edu.cn) or D.L. (email: liudongsheng@tsinghua.edu.cn)

Table of Contents

S1 Thermal denaturation test 1
- **S1.1 Detailed designs of addressable structures with ligatable nicks 1**
- **S1.2 AGE results of structures with ligatable nicks 2**
- **S1.3 Analysis results of survival rate and melting temperature 9**
- **S1.4 Full-size AFM images 10**

S2 Calibration of ligation efficiency 16
- **S2.1 Design of the 2 × 2 lattice 16**
- **S2.2 Characterization of self-assembly of the 2 × 2 lattice 16**
- **S2.3 Characterization of correlation between self-assembly and ligation 17**
- **S2.4 Detailed description of ligation efficiency calculation 19**

S3 Analysis of ligation performance 20
- **S3.1 Population analysis of ligation products 20**
- **S3.2 Design of the 3 × 3 lattice 21**
- **S3.3 Characterization of the 3 × 3 lattice of ligation treatment 22**
- **S3.4 Analysis of population distribution of ligated structures 24**

S4 Simulation of lattice integrity after ligation 25

S5 AGE results of structures without ligatable nicks 28

S6 Morphology investigation of mechanical stability 29

S7 Dynamic structural reconfiguration by enzymatic ligation 30
- **S7.1 Loss of ligation function based structural reconfiguration 30**
- **S7.2 Gain of ligation function based structural reconfiguration 34**

References 38

Sequences 39

S1 Thermal denaturation test

S1.1 Detailed designs of addressable structures with ligatable nicks

Addressable structures entirely from short synthetic strands were adopted in this study. A certain edge of a structure consists of two root domains (double-stranded) and a stem domain (single-stranded) which is flanked by a pair of ligatable nicks (Figure S1).

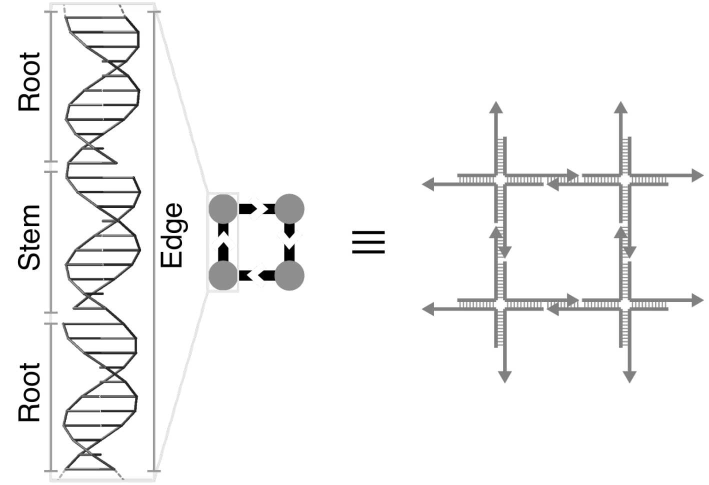

Figure S1. Schematic diagrams of a typical edge of ligatable nicks. A typical edge resulting from base pairing of two complementary stem domains appended to the respective root domains.

We implemented thermal denaturation test on six addressable lattices (J4-I, J4-II, J4-III, J3, DX-I and DX-II) with and without ligation treatment. Detailed designs of the lattices are shown in Figure S2.

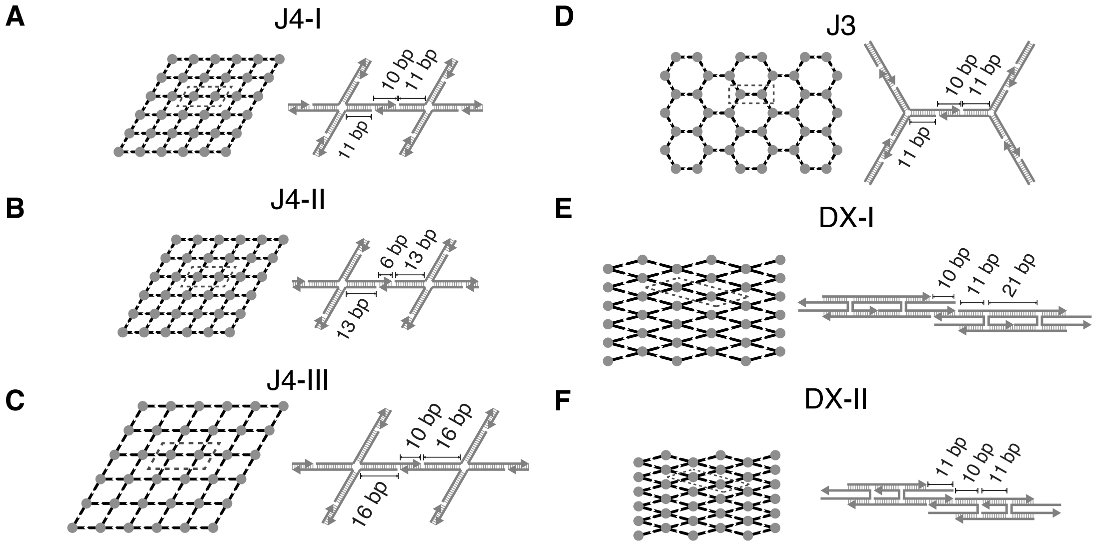

Figure S2. Schematics of six lattices. (A-C) 6 × 6 lattices from 4-arm junction motifs. 11-bp root domains/10-bp stem domains for J4-I (A), 13-bp root domains/6-bp stem domains for J4-II (B), and 16-bp root domains/10-bp stem domains for J4-III (C). (D) 6 × 7 lattice from 3-arm junction motifs. (E and F) 6 × 6 lattices from DX motifs. 21-bp rigidity domains/10-bp sticky domains for DX-I (E) and 11-bp rigidity domains/11-bp sticky domains for DX-II (F). Left: schematics of the lattices and dash boxes highlight component motifs with strand level of details.

S1.2 AGE results of structures with ligatable nicks

Full-size representative AGE results of six lattices (J4-I, J4-II, J4-III, J3, DX-I and DX-II) under certain thermal incubation temperature ranges are shown in Figure S3 - Figure S8 respectively. The number above each lane denotes the corresponding incubation temperature. Incubation temperature ranges and total numbers of data points of unligated (U) and ligated (L) lattices are listed in Table S1.

Table S1. Thermal incubation temperature ranges and total numbers of data points of the 6 lattices.

	J4-I	J4-II	J4-III	J3	DX-I	DX-II
U [°C] (data points)	35-75 (19 × 3)	30-75 (22 × 3)	35-75 (19 × 3)	30-75 (22 × 3)	40-65 (13 × 3)	35-65 (14 × 3)
L [°C] (data points)	55-95 (19 × 3)	55-95 (19 × 3)	55-95 (19 × 3)	55-95 (19 × 3)	60-85 (18 × 3)	40-85 (21 × 3)

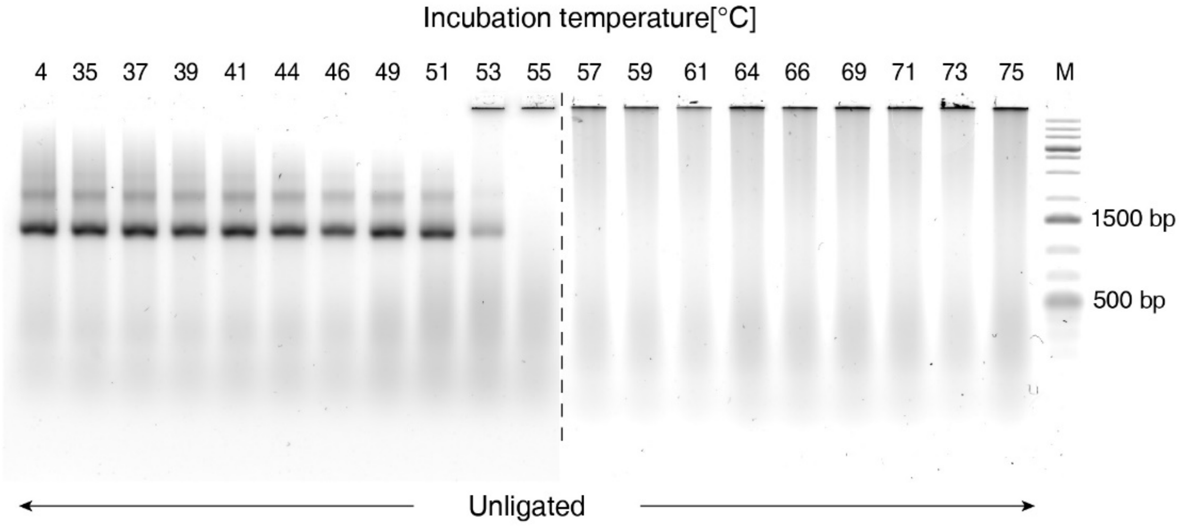

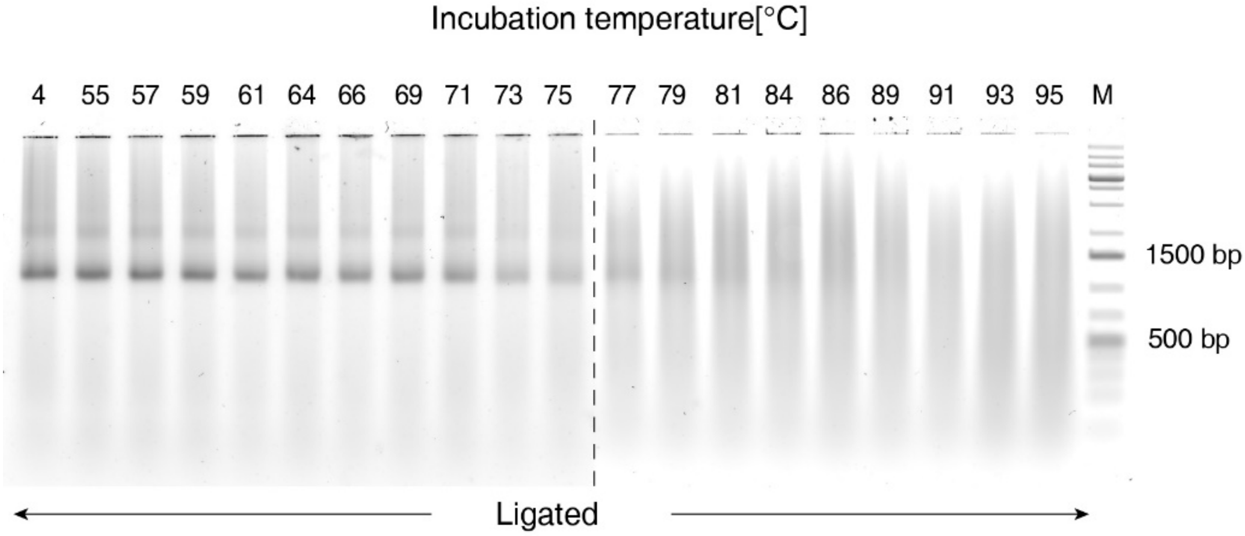

Figure S3. AGE results of lattice J4-I after thermal incubation. Top: AGE results of unligated lattice J4-I. Bottom: AGE results of ligated lattice J4-I. Numbers above gel lanes indicate corresponding incubation temperatures. Lane M: 1-kb ladder.

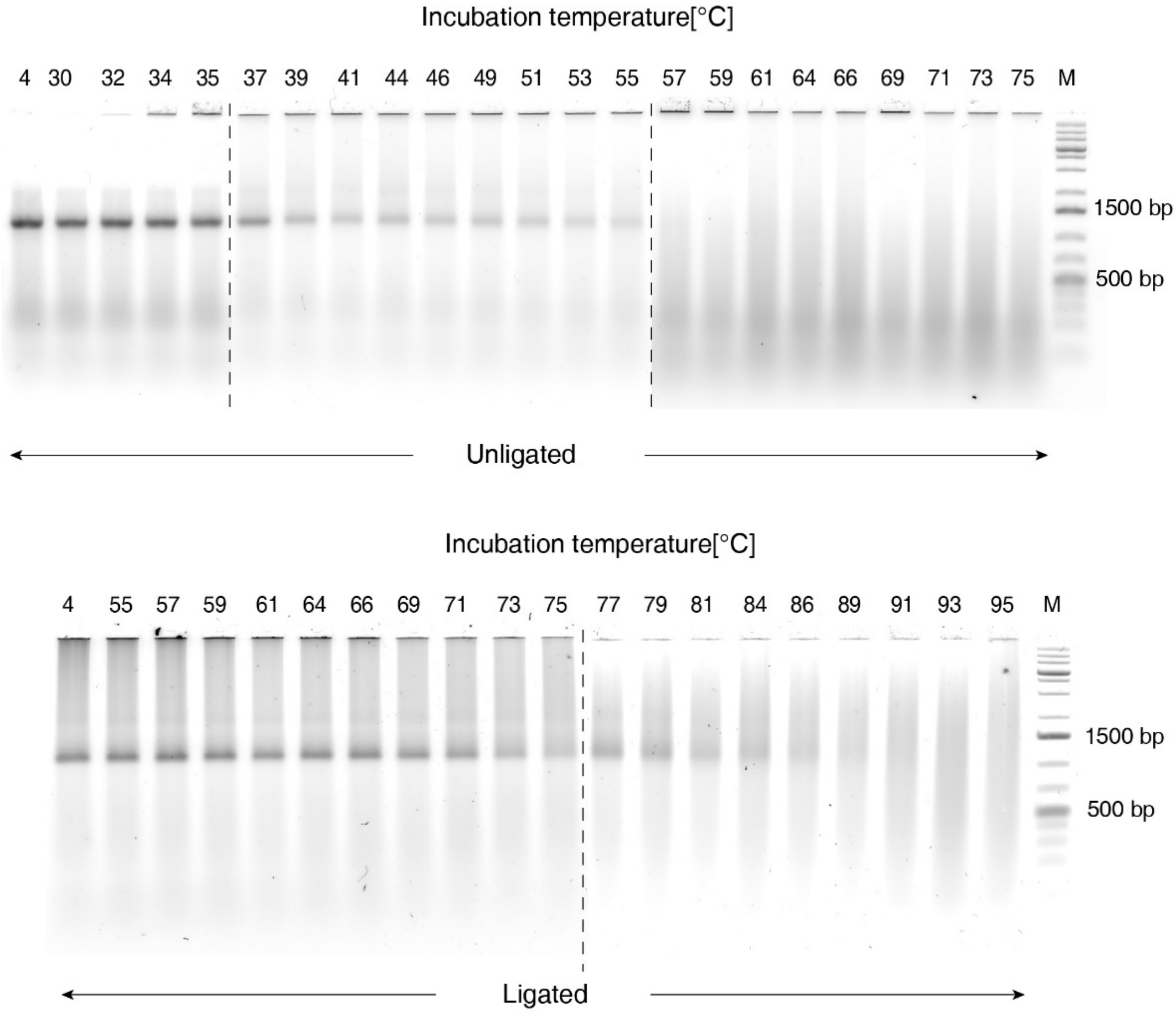

Figure S4. AGE results of lattice J4-II after thermal incubation. Top: AGE results of unligated lattice J4-II. Bottom: AGE results of ligated lattice J4-II. Numbers above gel lanes indicate corresponding incubation temperatures. Lane M: 1-kb ladder.

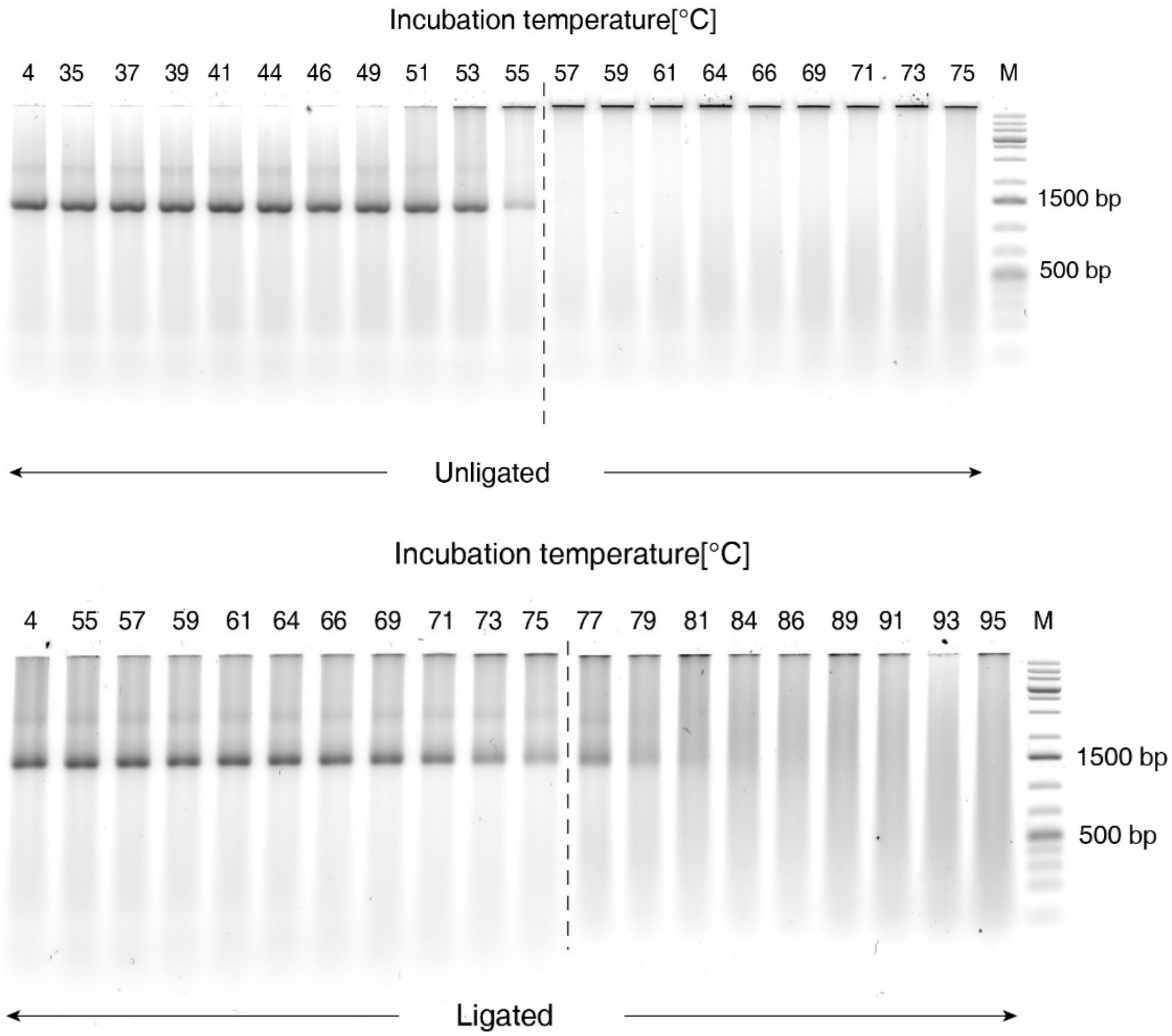

Figure S5. AGE results of lattice J4-III after thermal incubation. Top: AGE results of unligated lattice J4-III. Bottom: AGE results of ligated lattice J4-III. Numbers above gel lanes indicate corresponding incubation temperatures. Lane M: 1-kb ladder.

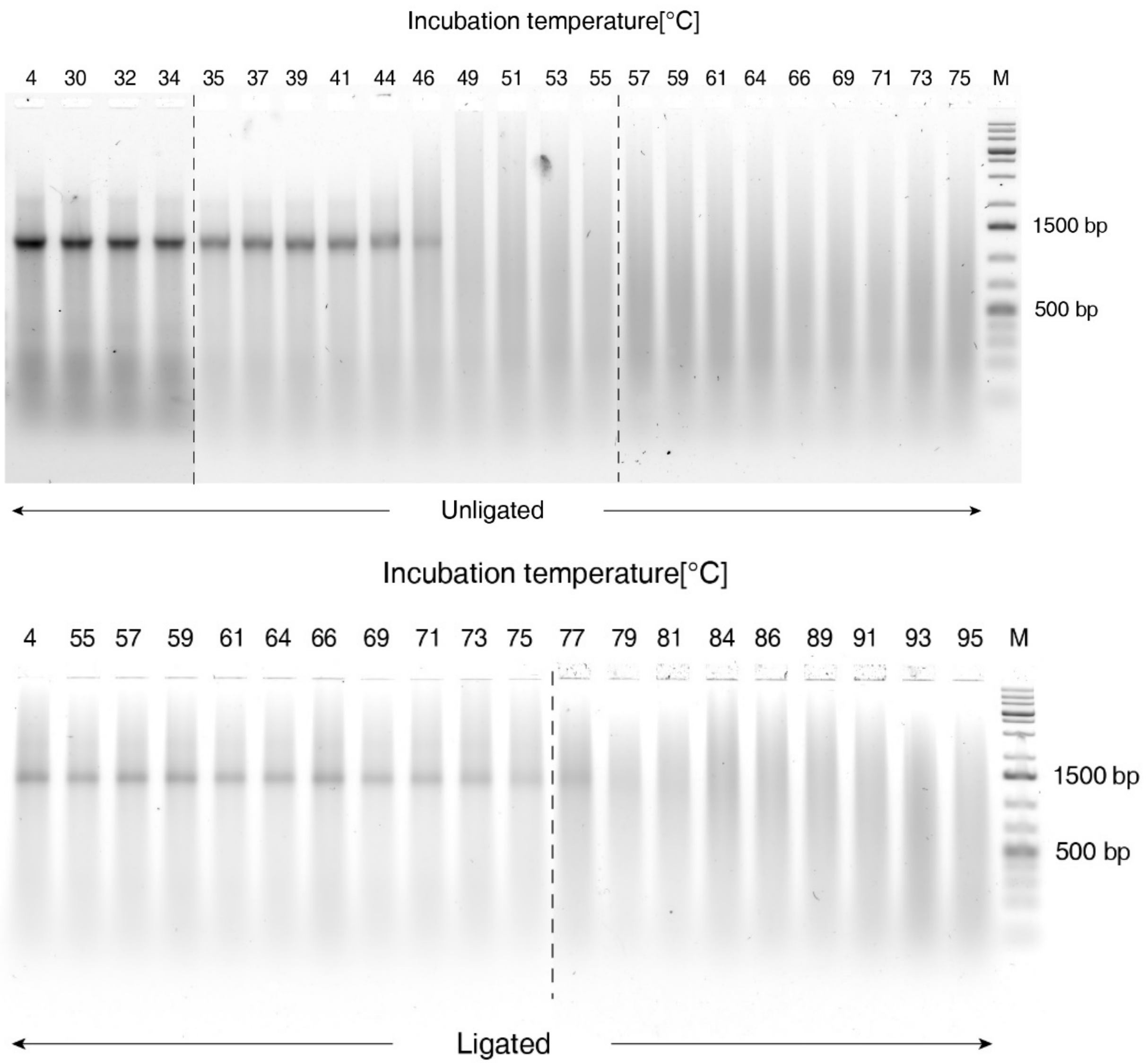

Figure S6. AGE results of lattice J3 after thermal incubation. Top: AGE results of unligated lattice J3. Bottom: AGE results of ligated lattice J3. Numbers above gel lanes indicate corresponding incubation temperatures. Lane M: 1-kb ladder.

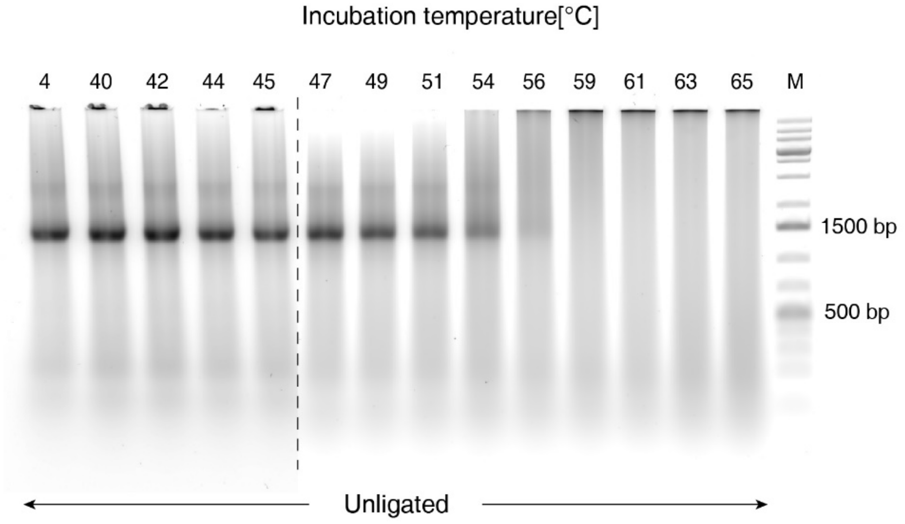

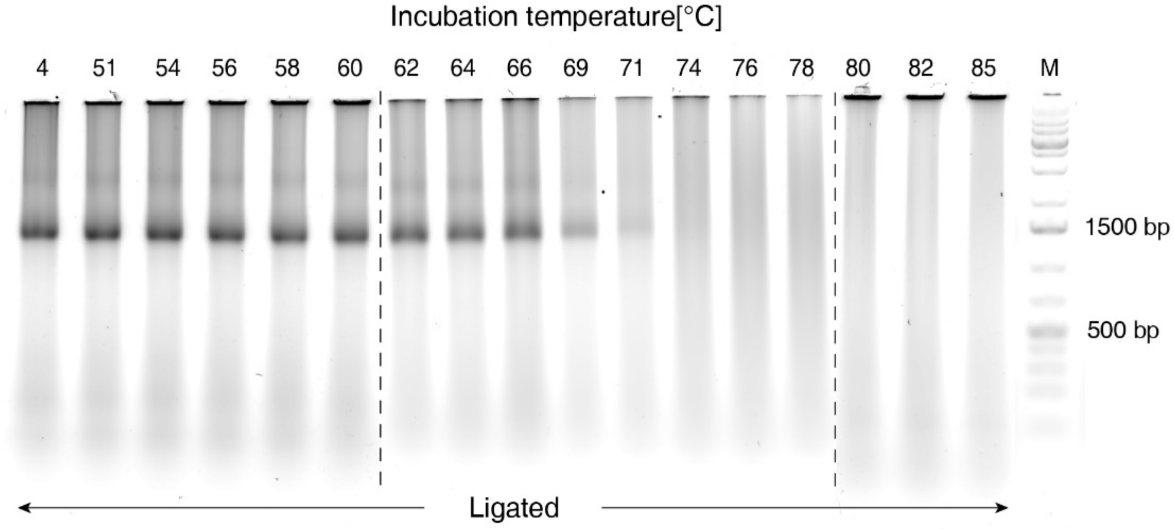

Figure S7. AGE results of lattice DX-I after thermal incubation. Top: AGE results of unligated lattice DX-I. Bottom: AGE results of ligated lattice DX-I. Numbers above gel lanes indicate corresponding incubation temperatures. Lane M: 1-kb ladder.

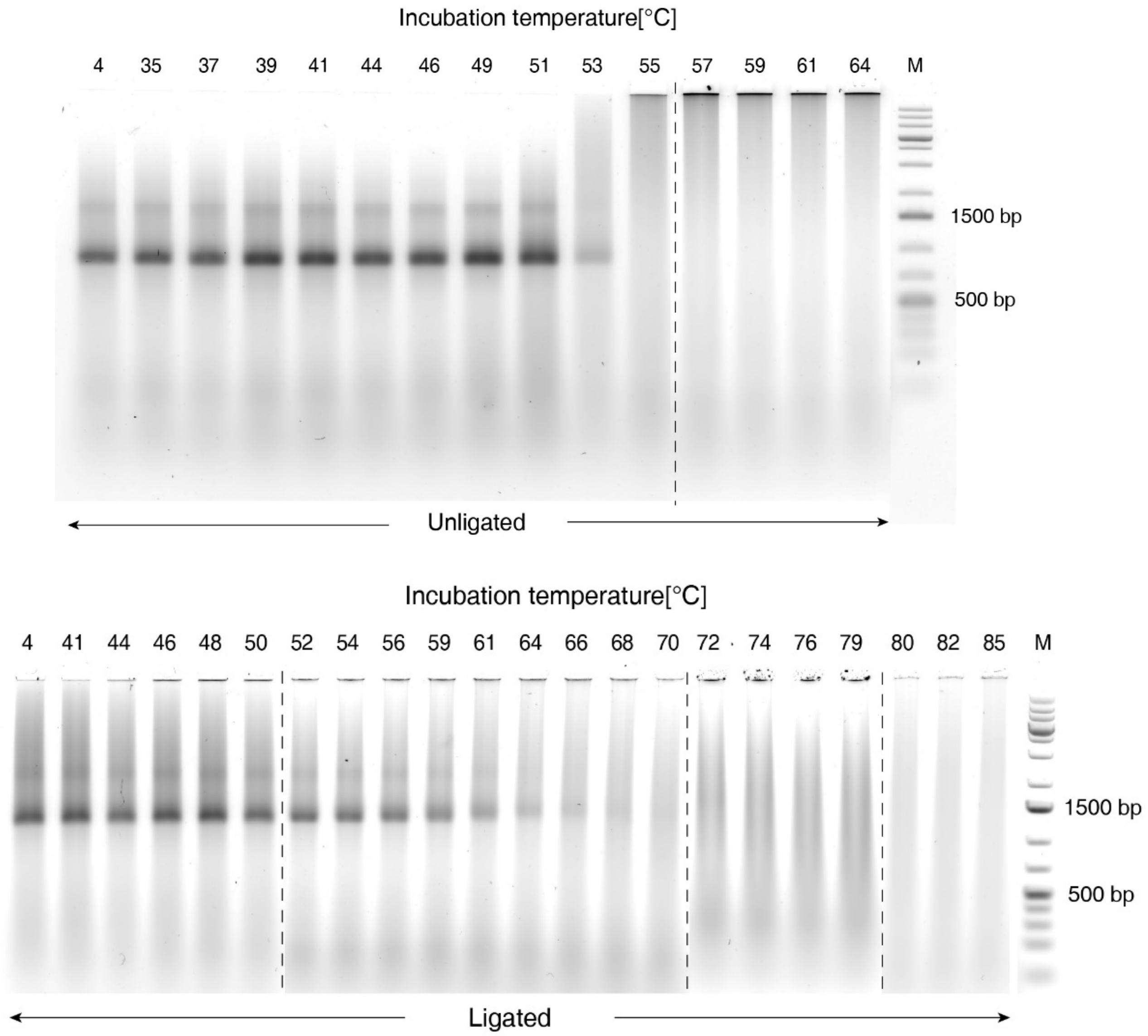

Figure S8. AGE results of lattice DX-II after thermal incubation. Top: AGE results of unligated lattice DX-II. Bottom: AGE results of ligated lattice DX-II. Numbers above gel lanes indicate corresponding incubation temperatures. Lane M: 1-kb ladder.

S1.3 Analysis results of survival rate and melting temperature

According to AGE results of the six lattice species (J4-I, J4-II, J4-III, J3, DX-I and DX-II), after a certain incubation temperature, no band was available but a low level of fluorescence was shown across the entire gel lane. A background subtraction was applied to better present the survival rates of the temperature range. Survival rates before and after background subtraction based on AGE results of ligated lattice J4-I (Figure S3, bottom) are shown in Figure S9. Such a background subtraction was applied in all 6 lattices for the plots shown in Figures 2 and 3.

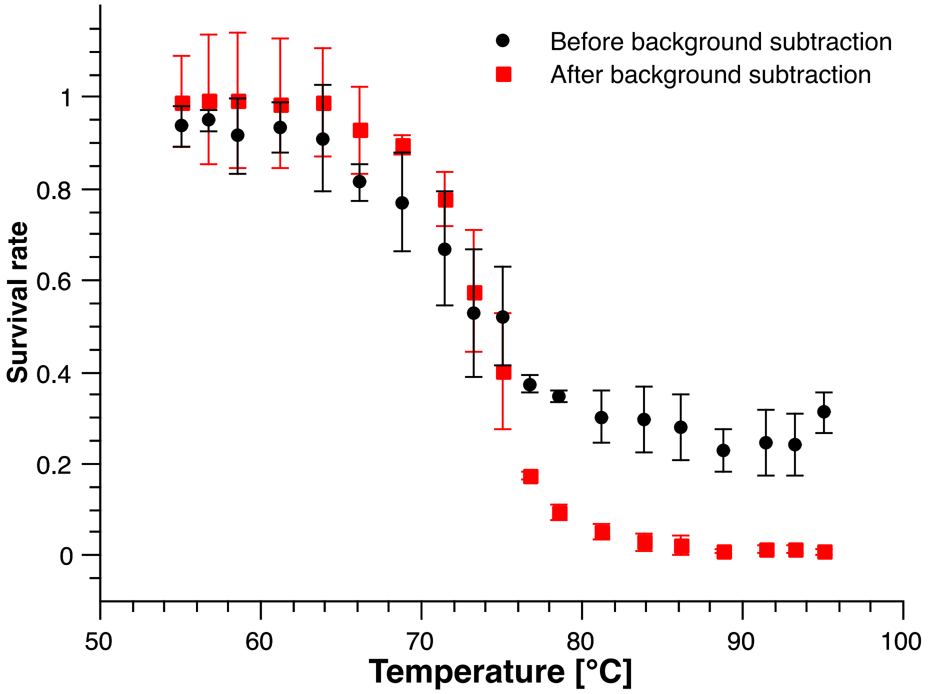

Figure S9. Survival rates of ligated lattice J4-I. Black dots and red squares represent average survival rates before and after background subtraction respectively.

We obtained six pairs of melting curves of the six lattice species by fitting the dataset to a Boltzmann sigmoid except for the one of unligated lattice J4-II, which was a double Boltzmann sigmoidal fit. The melting temperatures of 6 lattice species were calculated based on the melting curves shown in Figures 2 and 3 and results are listed in Table S2. The melting temperature of ligated lattice DX-I is slightly lower than those of ligated wireframe lattices (J4-I, J4-II, J4-III and J3), which is presumably due to the limited accessibility of the core nicks in DX motifs[1]. An even lower melting temperature of ligated lattice DX-II may be attributed to the unligatable core nicks within the structure.

Table S2. Melting temperature (T_m) of 6 lattice species

	**J4-I**	**J4-II**	**J4-III**	**J3**	**DX-I**	**DX-II**
**T_m (unligated) [°C]**	53	37	55	45	53	53
**T_m (ligated) [°C]**	74	75	76	75	71	62
**ΔT_m [°C]**	21	38	21	30	18	9

Notes: The double Boltzmann fit of unligated lattice J4-II resulted in two melting temperatures (55°C and 37°C), which we presumed corresponded to the domain length difference of the 13-nt root domains and the 6-nt stem domains. The melting temperature (37°C) of the 6-nt stem domains was adopted as that of the unligated lattice J4-II as the structure would no longer exist if the 6-nt stem domains disassociated.

S1.4 Full-size AFM images

Full-size AFM images of J4-I, J4-II, J4-III, J3, DX-I and DX-II of certain survival rates or at select temperatures are shown in Figure S10 - Figure S15 respectively.

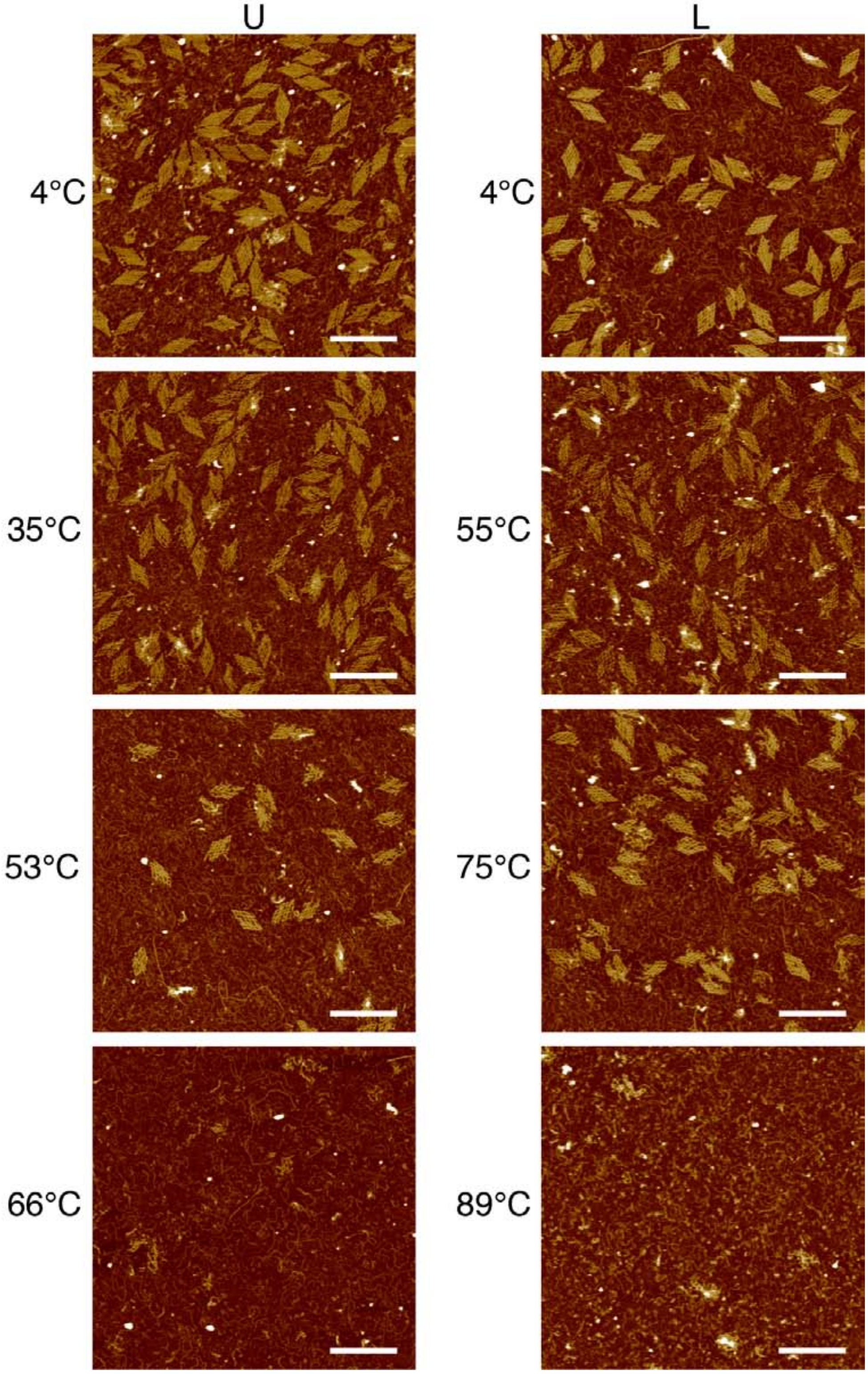

Figure S10. Full-size AFM images of lattice J4-I after thermal denaturation test. Left: images of unligated lattices (U) incubated at specific temperatures. Right: images of ligated lattices (L) incubated at specific temperatures. Temperatures on the left side of images indicate corresponding incubation conditions. Scale bars: 200 nm.

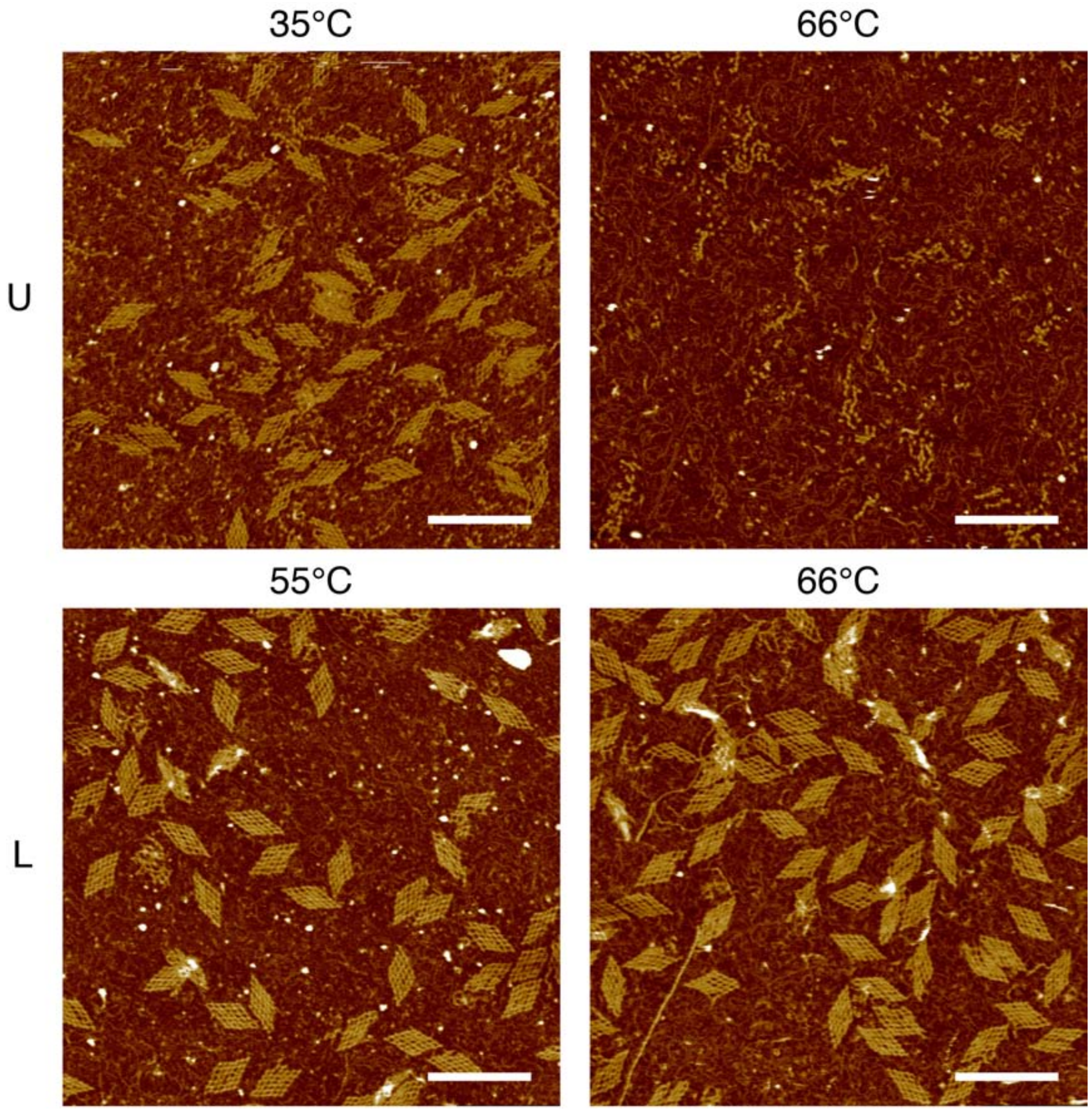

Figure S11. Full-size AFM images of lattice J4-II after thermal denaturation test. Top: images of unligated lattices (U) incubated at specific temperatures. Bottom: images of ligated lattices (L) incubated at specific temperatures. Temperatures above images indicate corresponding incubation conditions. Scale bars: 200 nm.

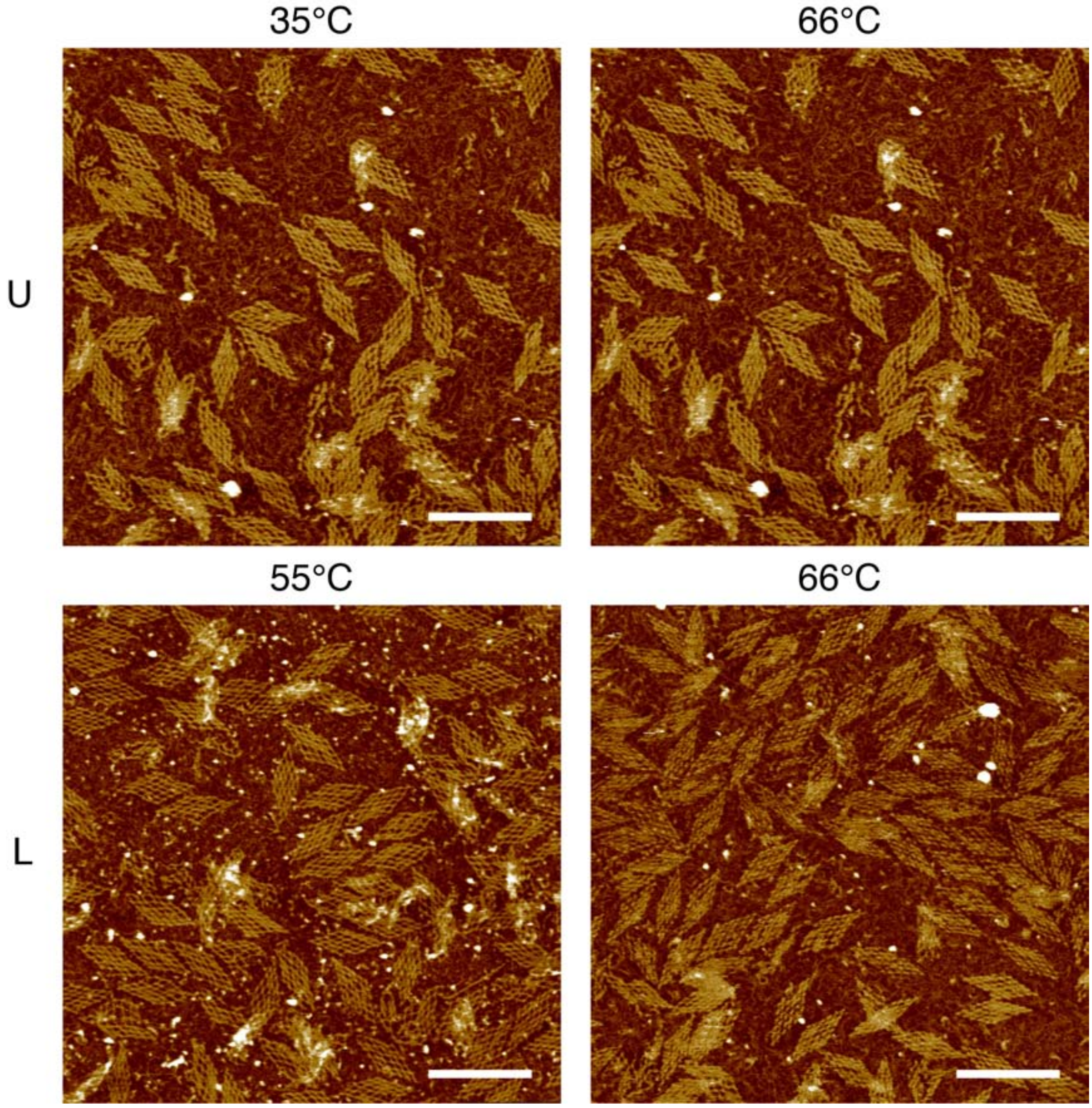

Figure S12. Full-size AFM images of lattice J4-III after thermal denaturation test. Top: images of unligated lattices (U) incubated at specific temperatures. Bottom: images of ligated lattices (L) incubated at specific temperatures. Temperatures above images indicate corresponding incubation conditions. Scale bars: 200 nm.

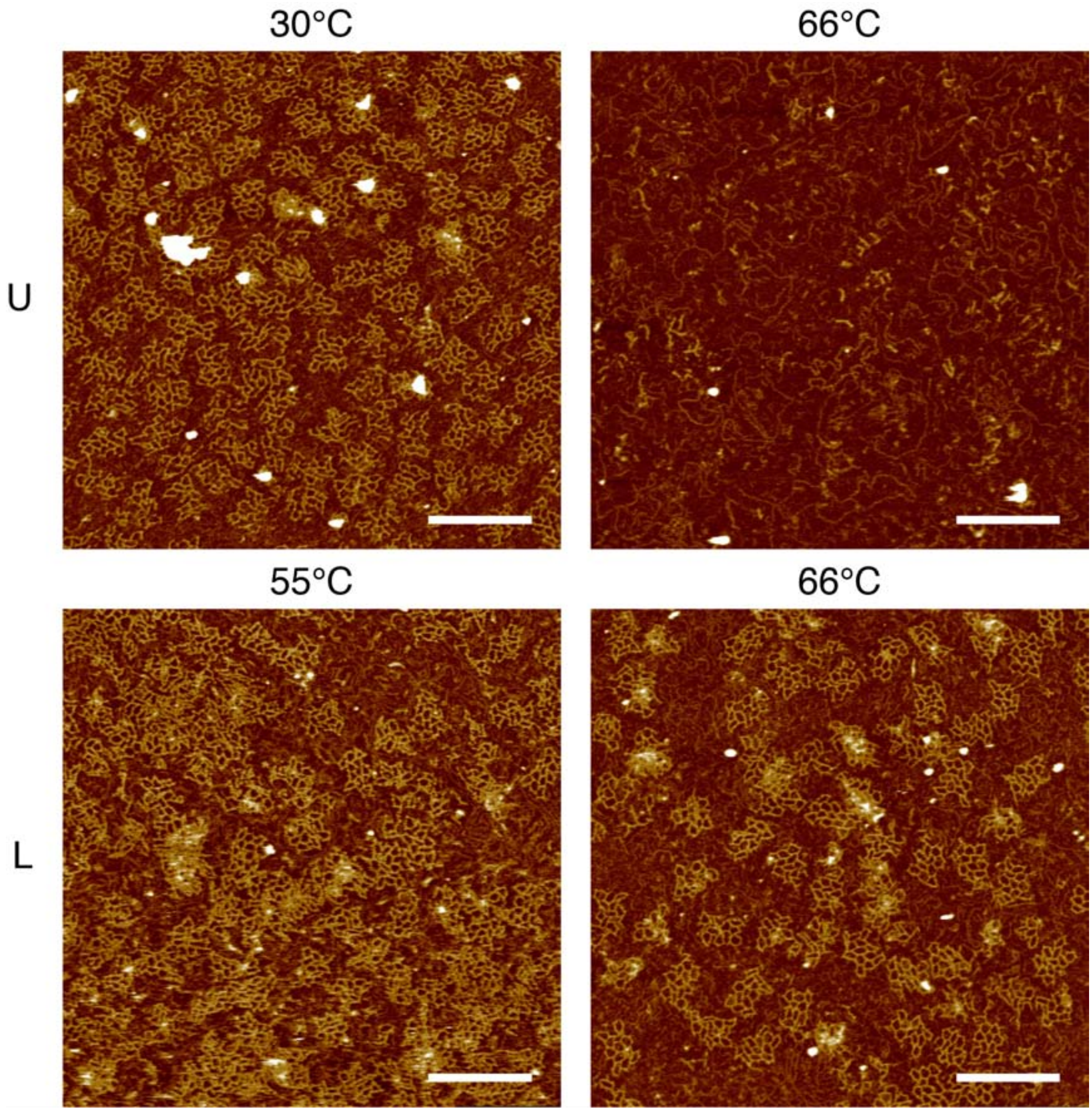

Figure S13. Full-size AFM images of lattice J3 after thermal denaturation test. Top: images of unligated lattices (U) incubated at specific temperatures. Bottom: images of ligated lattices (L) incubated at specific temperatures. Temperatures above images indicate corresponding incubation conditions. Scale bars: 200 nm.

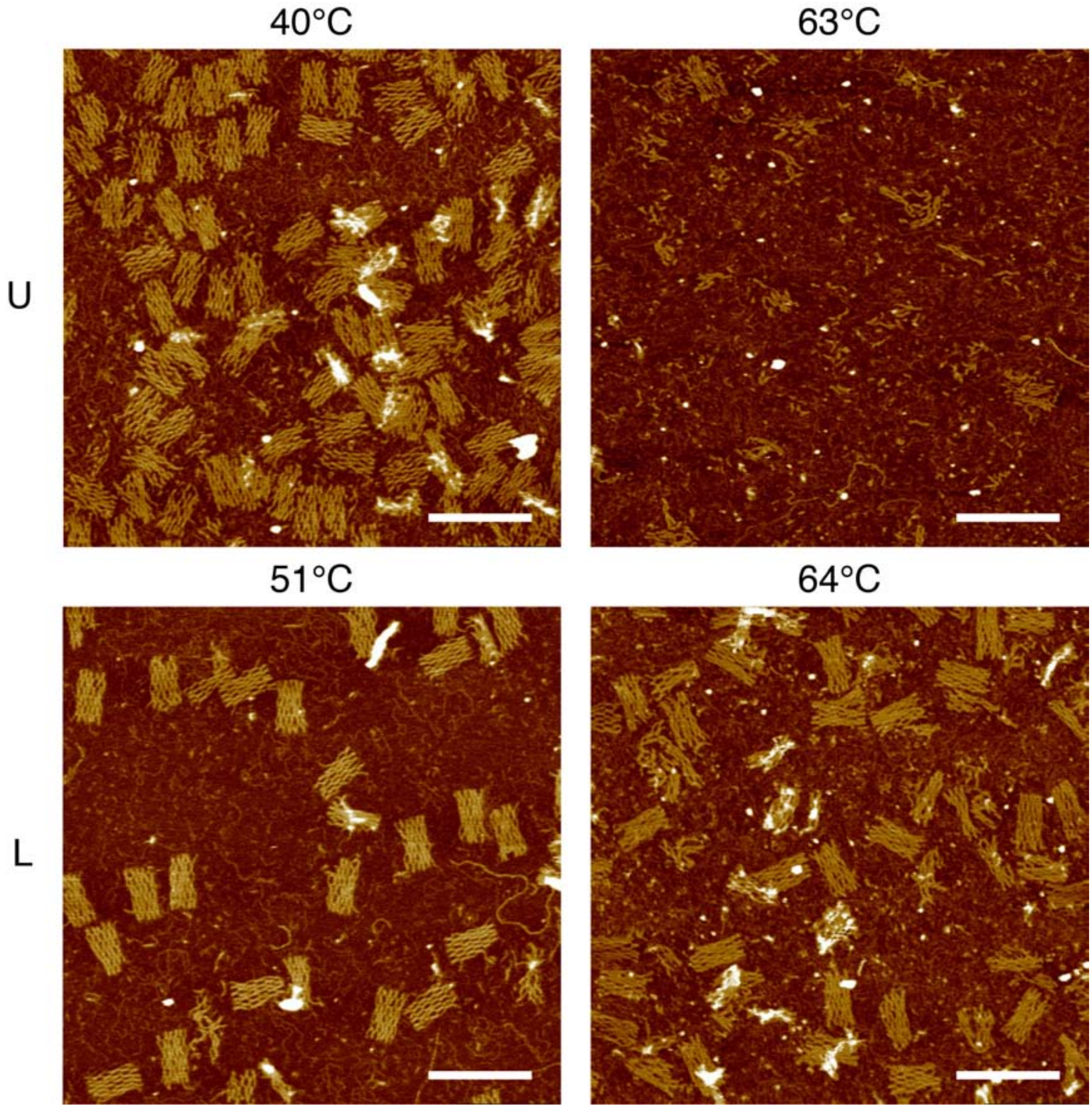

Figure S14. Full-size AFM images of lattice DX-I after thermal denaturation test. Top: images of unligated lattices (U) incubated at specific temperatures. Bottom: images of ligated lattices (L) incubated at specific temperatures. Temperatures above images indicate corresponding incubation conditions. Scale bars: 200 nm.

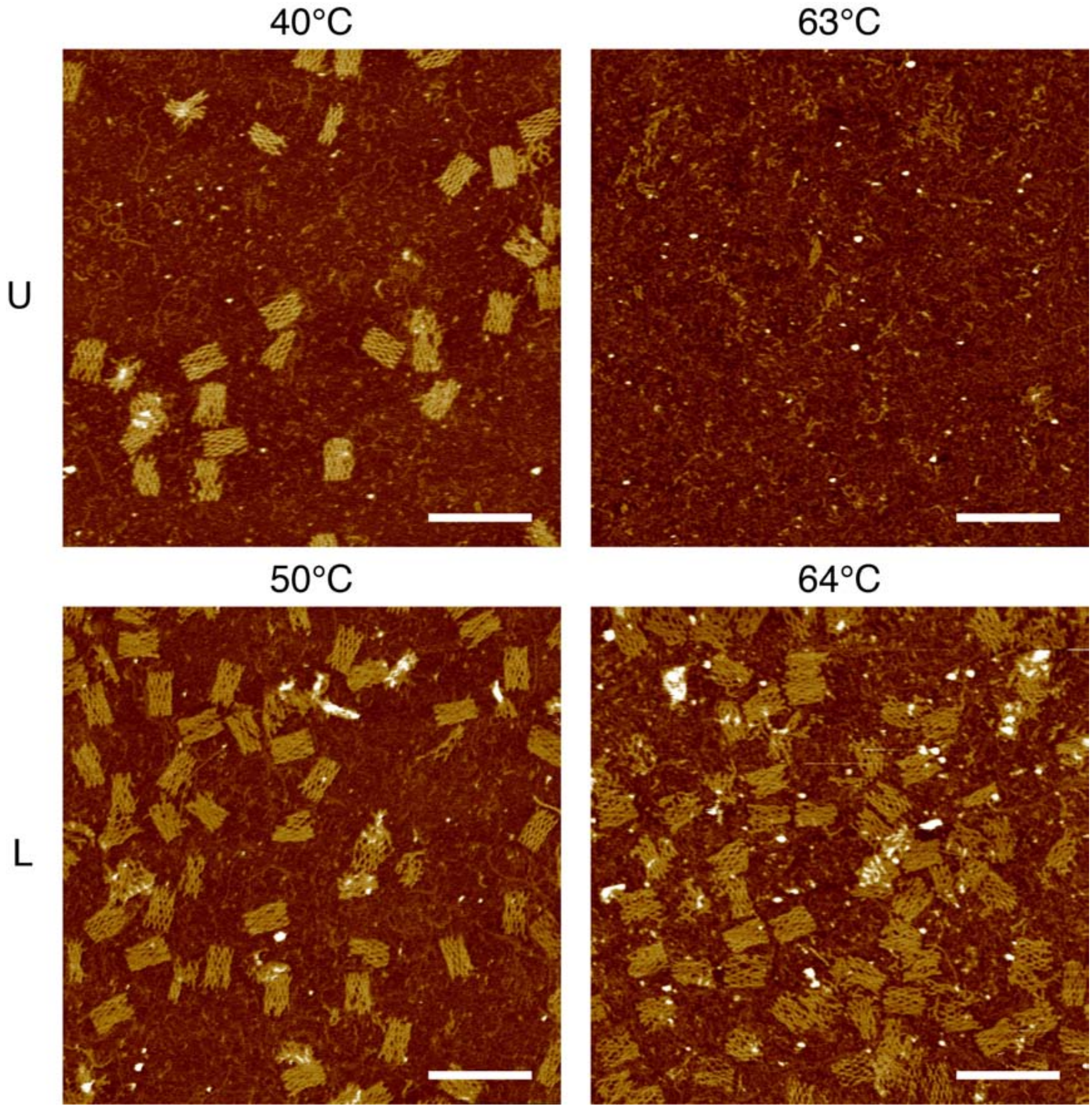

Figure S15. Full-size AFM images of lattice DX-II after thermal denaturation test. Top: images of unligated lattices (U) incubated at specific temperatures. Bottom: images of ligated lattices (L) incubated at specific temperatures. Temperatures above images indicate corresponding incubation conditions. Scale bars: 200 nm.

S2 Calibration of ligation efficiency

S2.1 Design of the 2 × 2 lattice

A 2 × 2 lattice of 4-arm junction motifs was adopted for calibration of the ligation efficiency. Each arm of the motif has an 11-bp root domain (double-stranded) and a 10-bp stem domain (single-stranded) with a typical edge resulting from base pairing of two complementary stem domains appended to the respective root domains (Figure S16).

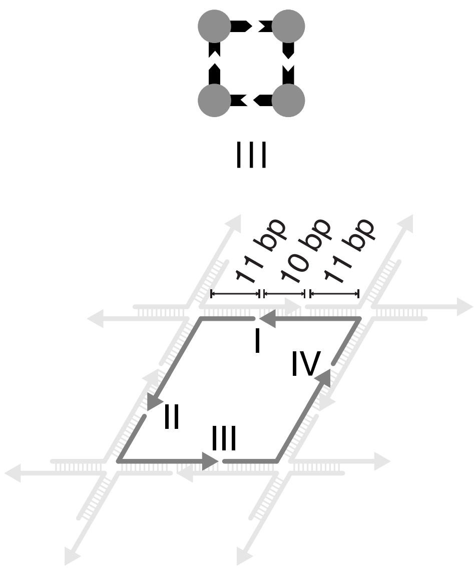

Figure S16. Schematics of the 2 × 2 lattice. Four component strands (highlighted in dark grey) corresponding to four nicks (I, II, III and IV) are presented for ligation treatment.

S2.2 Characterization of self-assembly of the 2 × 2 lattice

We calculated the assembly yield of the 2 × 2 lattice based on AGE result (Figure S17 bottom). The average yield was calculated as 84% ± 4% (N=3).

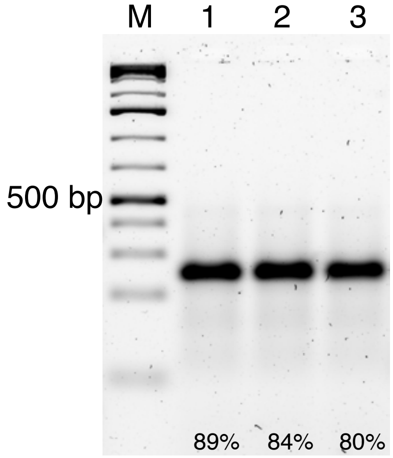

Figure S17. AGE result of the self-assembled 2 × 2 lattice. Lane M contains 1-kb ladder. Lanes 1-3 contain the triplicate of the self-assembled 2 × 2 lattice. The assembly yield of each sample is presented at the bottom.

S2.3 Characterization of correlation between self-assembly and ligation

We compared the ligation products of the 2 × 2 lattices that have one (I), two (I and II), three (I, II and III) and four (I, II, III and IV) ligatable nicks respectively. The component strands corresponding to the nicks were 5' phosphorylated and self-assembled with the rest component strands prior to ligation. The 2 × 2 lattices of one to three ligatable nicks resulted in linear ligation products with length of multiple times of 32 nucleotides (nt) whereas the 2 × 2 lattices of four ligatable nicks resulted in circular ligation products of 128 nt. For treatment for samples of one to three ligatable nicks, the ligation results of each sample pair with or without self-assembly were similar (Figure S18, lanes 1-6). Only the results of the sample pair of four ligatable nicks were substantially different ((Figure S18, lanes 7 and 8). The results indicate that the circular ligation product can better reveal the correlation between self-assembly and ligation. We further calculated the ratio between the top and the bottom band of each sample lane based on the denaturing PAGE result as shown in Figure S18.

Lanes	1	2	3	4	5	6	7	8
Nicks	I	I	I,II	I,II	I,II,III	I,II,III	I,II,III,IV	I,II,III,IV
Self-assembly	-	+	-	+	-	+	-	+

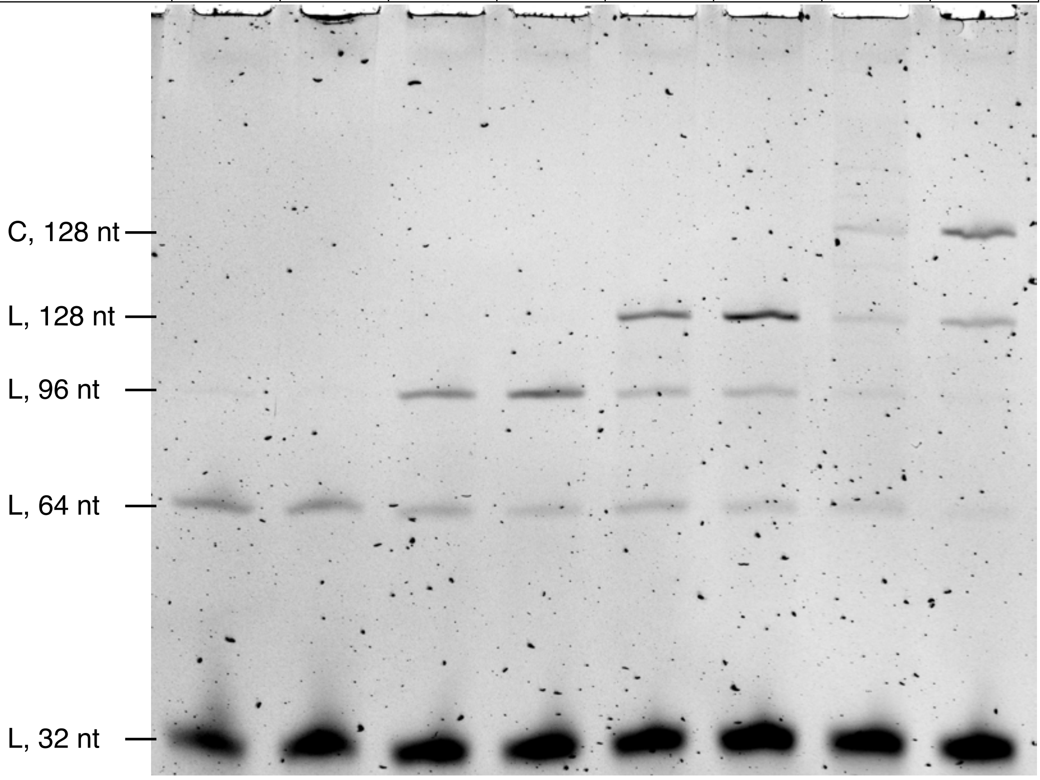

0.34 0.27 0.36 0.43 0.19 0.22 0.14 0.30

Figure S18. Denaturing PAGE result of the ligation products of the 2 × 2 lattice. Lanes 1, 3, 5 and 7 contain the ligation products of the 2 × 2 lattice without self-assembly of one (I), two (I and II), three (I, II and III) and four (I, II, III and IV) ligatable nicks respectively. Lanes 2, 4, 6 and 8 contain the ligation products of the 2 × 2 lattice with self-assembly of one (I), two (I and II), three (I, II and III) and four (I, II, III and IV) ligatable nicks respectively. L and C represent linear and circular ligation products respectively. The intensity ratio of the top and the bottom band in each lane is presented at the bottom.

As shown in Figure S19, the ligation of all four nicks highlighted (I, II, III and IV) resulted in a circular product (128 nt). Since the non-circular ligation products were generated regardless of the proper self-assembly of the designated structures, we focused on the circular product for the yield analysis.

Lanes	1	2	3	4
Phosphorylation	+	+	+	-
Self-assembly	+	-	+	+
Ligation	+	+	-	+

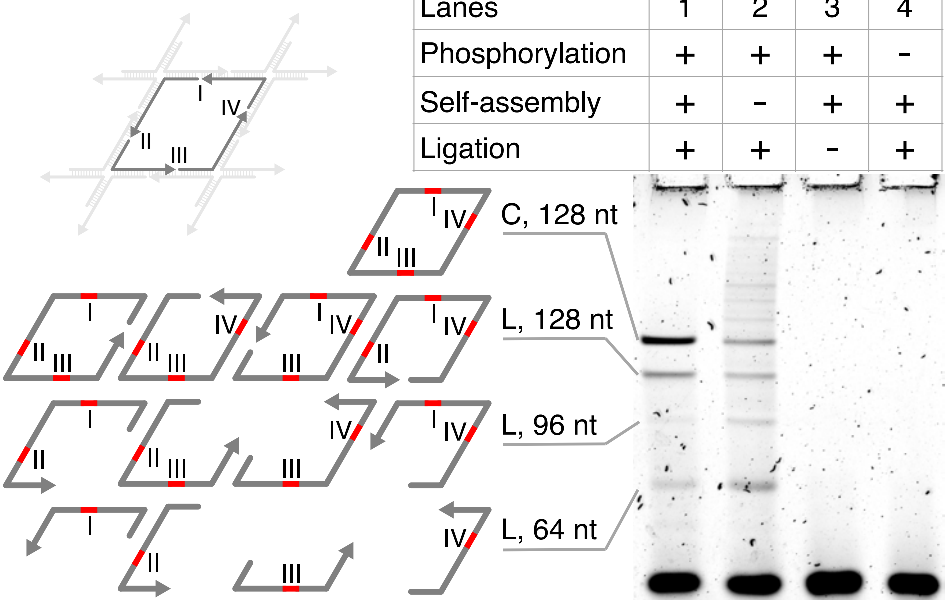

Figure S19. Characterization of the ligation products by denaturing PAGE. Left: schematics of different species of ligation products. Nicks sealed with phosphodiester bonds are highlighted in red. Right: denaturing PAGE results of the 2 × 2 lattice. Lane 1 contains the ligation products of the self-assembled 2 × 2 lattice whose four component strands have been 5’ phosphorylated. Lane 2 contains the products of the 2 × 2 lattice without self-assembly. Lane 3 contains the products of the 2 × 2 lattice without ligation. Lane 4 contains the products of the 2 × 2 lattice without phosphorylation. L and C represent linear and circular products.

S2.4 Detailed description of ligation efficiency calculation

We used the circular ligation product of the 2×2 lattice to estimate the ligation efficiency. Assume that ligation occurs with frequency x, the ratio of molecule number of 128-nt circular product (as in "C, 128 nt" band) to molecule number of 32-nt linear substrate (as in "L, 32 nt" band) will be as follows:

$$\frac{x^4}{12 + (1-x)^4 \times 4 + 4x(1-x)^3 \times 2 + 4x^2(1-x)^2 \times 1}$$

We calculated the ligation efficiency based on the denaturing PAGE result (Figure S20) and the average ligation efficiency was estimated as 0.89 ± 0.01 (N = 3). The calculation is based on the final yield after phosphorylation, self-assembly and ligation, and the ligation efficiency might be an underestimation.

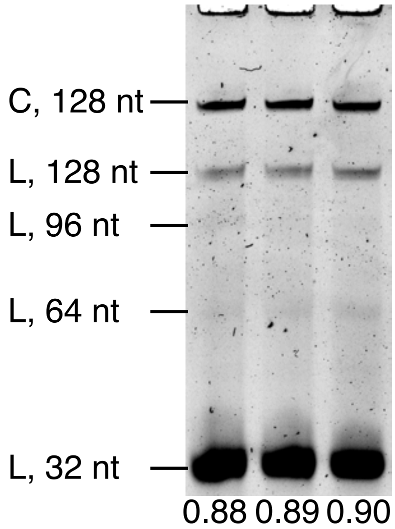

Figure S20. Denaturing PAGE result of the ligation products of the 2 × 2 lattice of four ligatable nicks. L and C represent linear and circular ligation products respectively. The ligation efficiency of each sample is presented at the bottom.

S3 Analysis of ligation performance

S3.1 Population analysis of ligation products

We hypothesize that the ligation products follow a binomial distribution, where each individual nick would be either ligated or not ligated, with a probability of p (ligation efficiency) for a ligated event and the total number of independent events n is the number of ligatable nicks present in the structure. The probability $P(x)$ of x ligated nicks is calculated as:

$$P(x) = \frac{n!}{(n-x)!} p^x (1-p)^{n-x}$$

A typical population distribution of ligation products with 24 nicks after ligation treatment is shown in Figure S21, where n = 24 and p is estimated at 0.8, 0.9 or 0.95 (Figure S21).

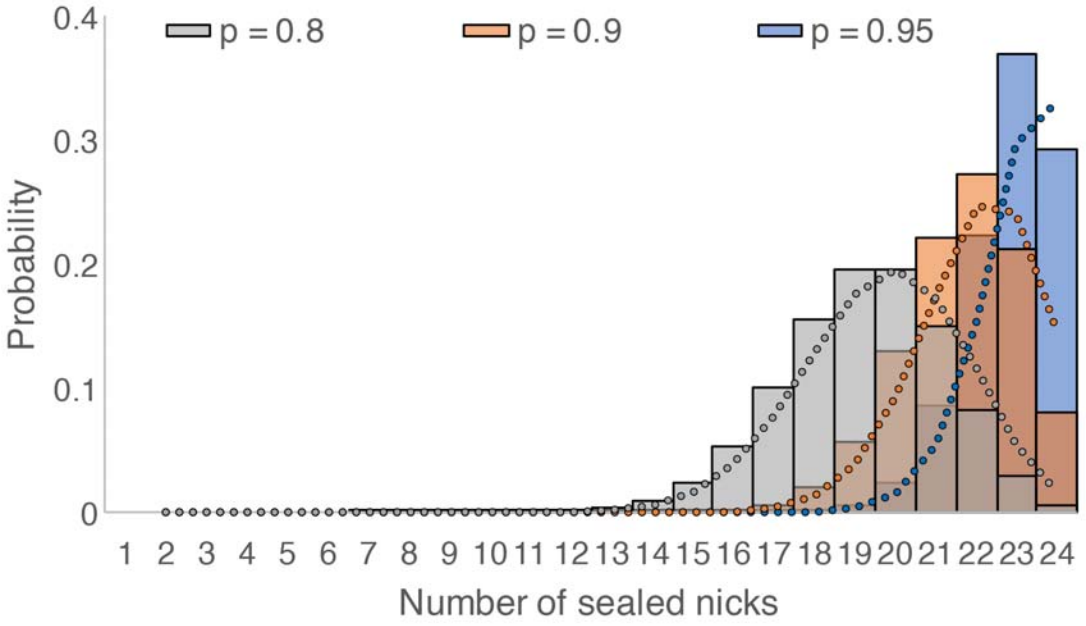

Figure S21. Population distribution of ligation products. An example of 24 nicks (n = 24) is presented with ligation efficiency (p) estimated at 0.8 (grey), 0.9 (orange) or 0.95 (blue).

S3.2 Design of the 3 × 3 lattice

We designed a 3 × 3 lattice of 4-arm junction motifs to investigate the ligation performance (Figure S22). The 3 × 3 lattice shared a similar motif domain design with the 2 × 2 lattice, which were a 11-bp root domain and a 10-bp stem domain. Based on the calibration results of the 2 × 2 lattice, we found that a circular product was more reliable when analyzing the ligation products. Therefore, the circumferential 4-arm junction motifs were adjusted so that the constituent strands were self-assembled into a 'closed' square shape without protruding overhangs (see Figure S22A for details). There are 24 strands in total corresponding to 24 ligatable nicks.

According to the calibration result in S2.4, the ligation efficiency for a certain nick is about 0.9. Based on our hypothesized binomial distribution, the majority of the ligated products should get most nicks sealed (≥20 nicks sealed, Figure S22B top) and a small portion of the ligated product should have all 24 nicks sealed (Figure S22B bottom) resulting in four small circles and one large circle interlocking in a chain mail fashion.

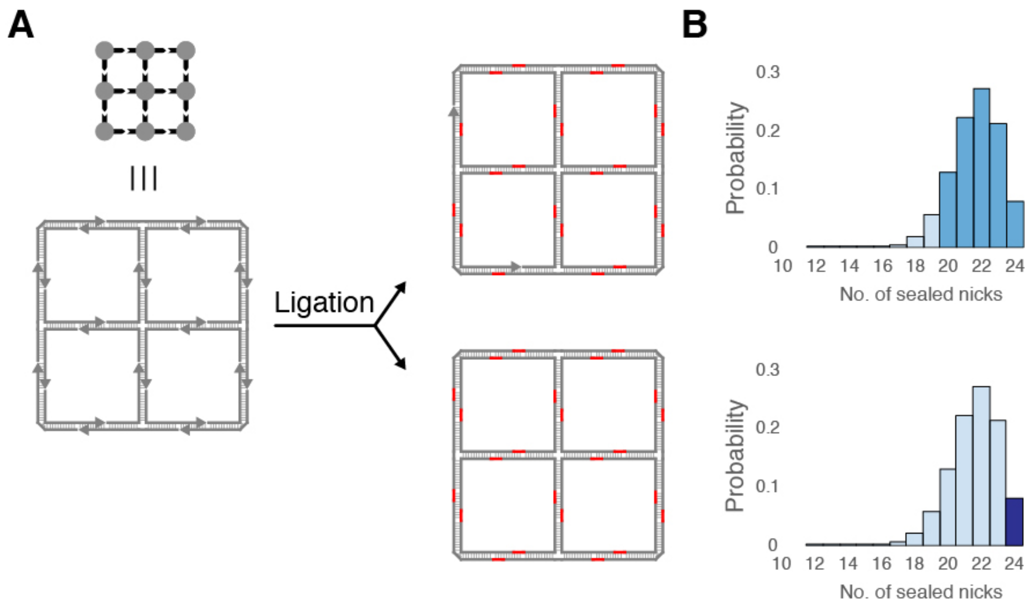

Figure S22. Enzymatic ligation treatment on the 3 × 3 lattice. (A) Schematics of enzymatic ligation treatment on the 3 × 3 lattice. Left, strand level design of the 3 × 3 lattice. Right, a typical ligation product with few remaining nicks is shown at the top and a nick-free ligation product is shown at the bottom (nicks sealed with phosphodiester bonds highlighted in red). (B) Bar charts showing hypothetical population distribution of ligated lattices with different numbers of sealed nicks. A large population of the lattices get most nicks sealed (≥20 nicks sealed; percentage shown as blue bars) and a small population get all the nicks sealed (24 nicks all sealed; percentage shown as navy bar).

S3.3 Characterization of the 3 × 3 lattice of ligation treatment

To investigate the 3 × 3 lattice after ligation treatment, we applied an exonuclease III digestion treatment, which initiated digestion at nicked sites in double-stranded DNA, to better distinguish the chain mail product out of the rest. The digested products were further collected by kit purification to remove enzymes and short oligos from the earlier rounds.

We characterized the chain mail product by AFM and the result showed different species of the products which were assumed to correspond to the stochastic ligation patterns of individual copies (Figure S23B).

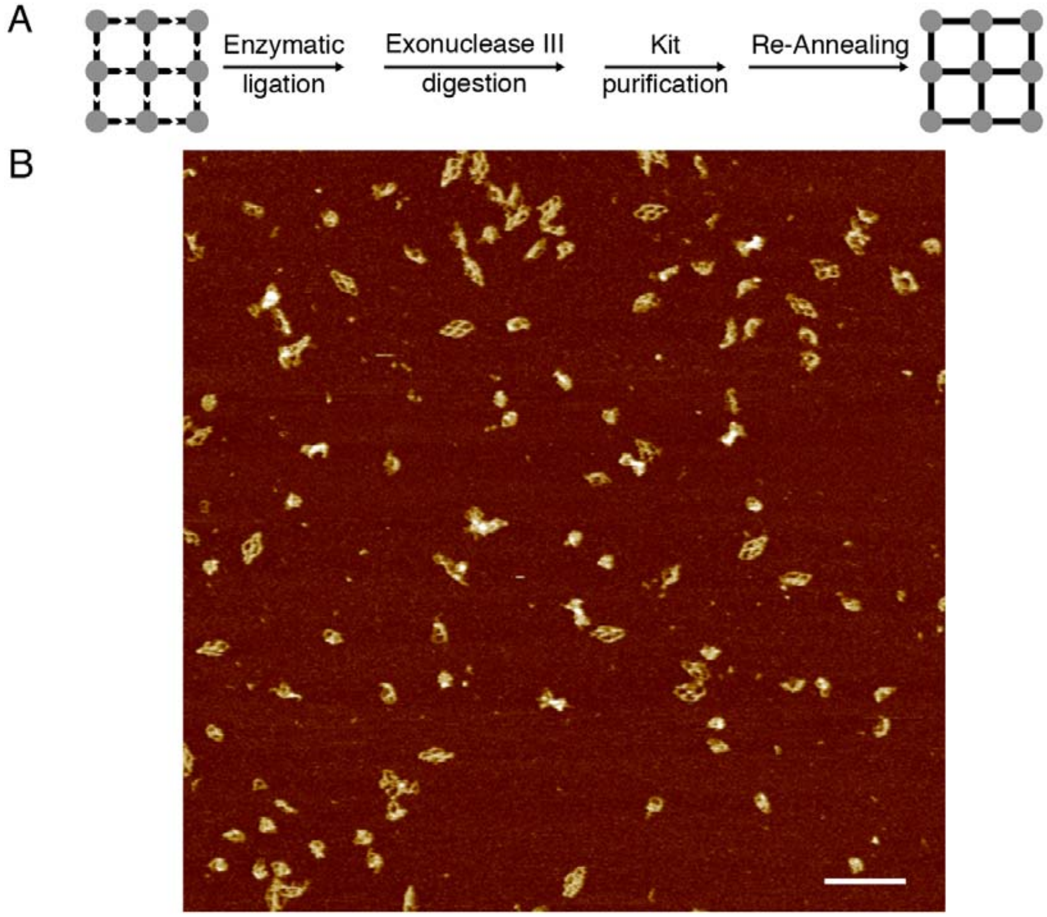

Figure S23. Full-size AFM image of the 3 × 3 lattice after ligation treatment and exonuclease III treatment. (A) Schematics of the workflow. (B) A representative AFM image. Scale bar: 100 nm.

We further characterized the chain mail product by AGE and the results showed that chain mail structures were resistant to stringent denaturing conditions and exonuclease digestion (Figure S24B, right), whereas unligated structures disassembled after treatments (Figure S24B, left).

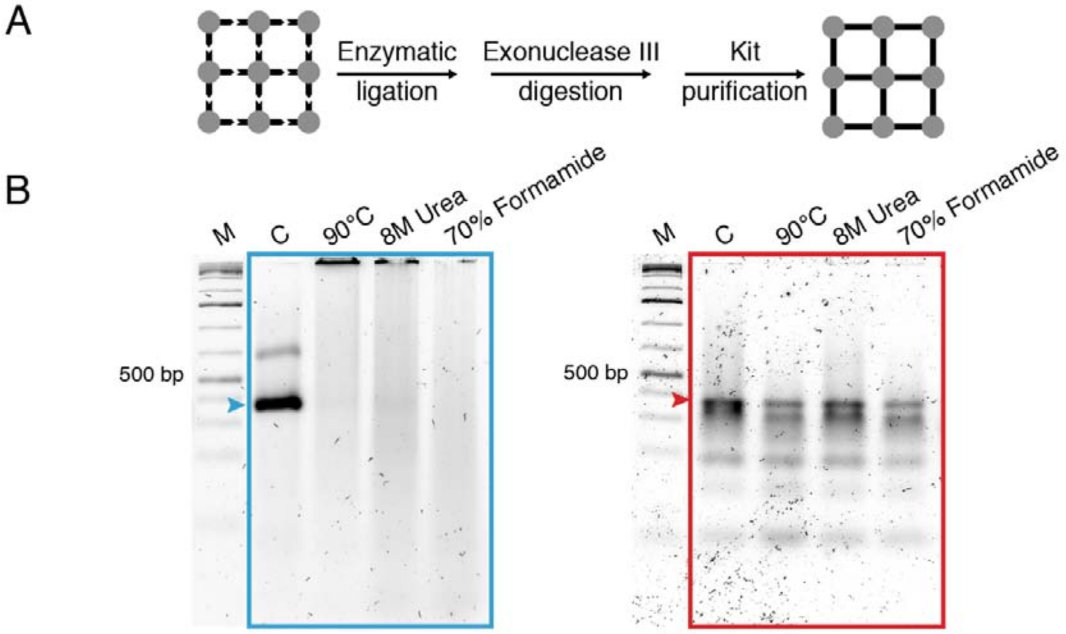

Figure S24. AGE results of chain mail structures under different treatments. (A) Schematics of the workflow. (B) AGE results of unligated (left) and completely ligated (right) structures. Treatment conditions are described above lanes. Lane C contains the control without any treatment. Lane M contains 1-kb ladder. Blue arrow points at unligated target structures. Red arrow points at completely ligated target structures.

S3.4 Analysis of population distribution of ligated structures

Since all the nicks of the 3 × 3 lattice are programmable, we can selectively engineer which nicks are ligatable. For example, when four nicks (I, II, III and IV) on the top left are selectively ligated, the resulting ligated product will be a small circular strand (Figure S25A). Likewise, when 8 circumferential nicks (I to VIII) are selectively ligated, the resulting ligated product will be a large circle (Figure S25B).

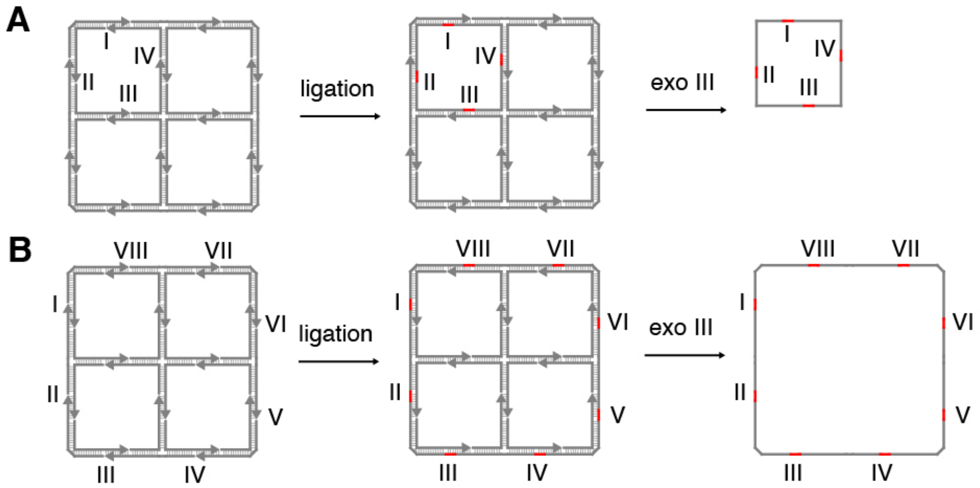

Figure S25. Enzymatic ligation treatment and digestion treatment on the 3 × 3 lattice of selective ligatable nicks. (A) A 3 × 3 lattice of four ligatable nicks (I to IV) results in a small circular product after ligation treatment and digestion treatment. (B) A 3 × 3 lattice of eight ligatable nicks (I to VIII) results in a large circular product after ligation treatment and digestion treatment. Nicks sealed with phosphodiester bonds highlighted in red.

We characterized the ligation products of seven distinct 3 × 3 lattices of specific patterns of ligatable nicks (8 to 24 ligatable nicks) and the AGE results indicated that the 3 × 3 lattice of 24 ligatable nicks was completely ligated (Figure S26). We further assessed the different species of the ligation products of the 3 × 3 lattice of 16 ligatable nicks (lane 7 in Figure S26) and the ligation efficiency was estimated to be around 0.9 compared to hypothetical distributions of the ligation products.

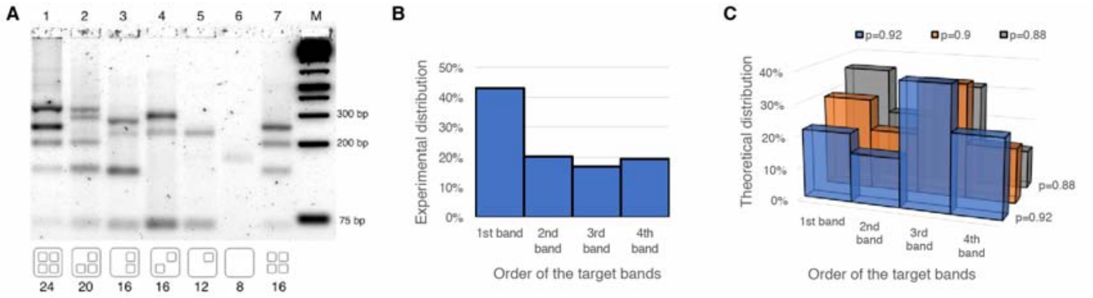

Figure S26. Ligation products of 3 × 3 lattices of different numbers of ligatable nicks. (A) AGE results. Lanes 1-7 contain 3 × 3 lattice of different numbers of ligatable nicks of unique patterns. The target ligation product and total ligatable nicks of each sample are presented at the bottom. Lane M contains 1-kb ladder. (B) Experimental population distribution of the 3 × 3 lattice of 16 ligatable nicks based on the AGE result of lane 7. (C) Hypothetical population distribution the 3 × 3 lattice of 16 ligatable nicks of four small circles with different ligation efficiency estimations. p = 0.88 in grey; p = 0.9 in orange; p = 0.92 in blue.

S4 Simulation of lattice integrity after ligation

We designed a simulation model to evaluate intact lattice percentage with gradient levels of ligation efficiency (0.0, 0.5, 0.6, 0.7, 0.8, 0.9). In our model, we implemented a Monte Carlo method to simulate the enhanced stability of the ligated structures.

A virtual lattice mesh (6 × 6 lattice or 6 × 6 lattice with a center hole) was placed for the component strands to map on. Four component strands were assigned for each 4-arm junction motif of the lattice and each component strand can be divided into three consecutive domains (11 nt, 11 nt and 10 nt) with 5' end at the first domain and 3' end at the third domain. When located in desired proximity, a nick site of a pair of 5' end and 3' end can form a covalent bond upon ligation.

In our model, certain criteria were set to evaluate the integrity of the resulted lattices survived in the elevated temperature which was above the melting temperature of a root/stem domain (10/11 bp) but below that of the combined segment of a root domain and a stem domain (21 bp) after ligation treatment. The dissociated or associated status of a component strand can be defined by whether and how the strand is locked in from specific ligation events.

1. When none of the nicks X_I, X_{II} and Y is ligated, the highlighted component strand was defined as status 1 (as shown in Figure S27A).
2. When neither X_I nor X_{II} is ligated but nick Y is ligated, the highlighted component strand was defined as status 2 (as shown in Figure S27B).
3. The ligation status of (status 1) ∪ (status 2) was defined as status 3.

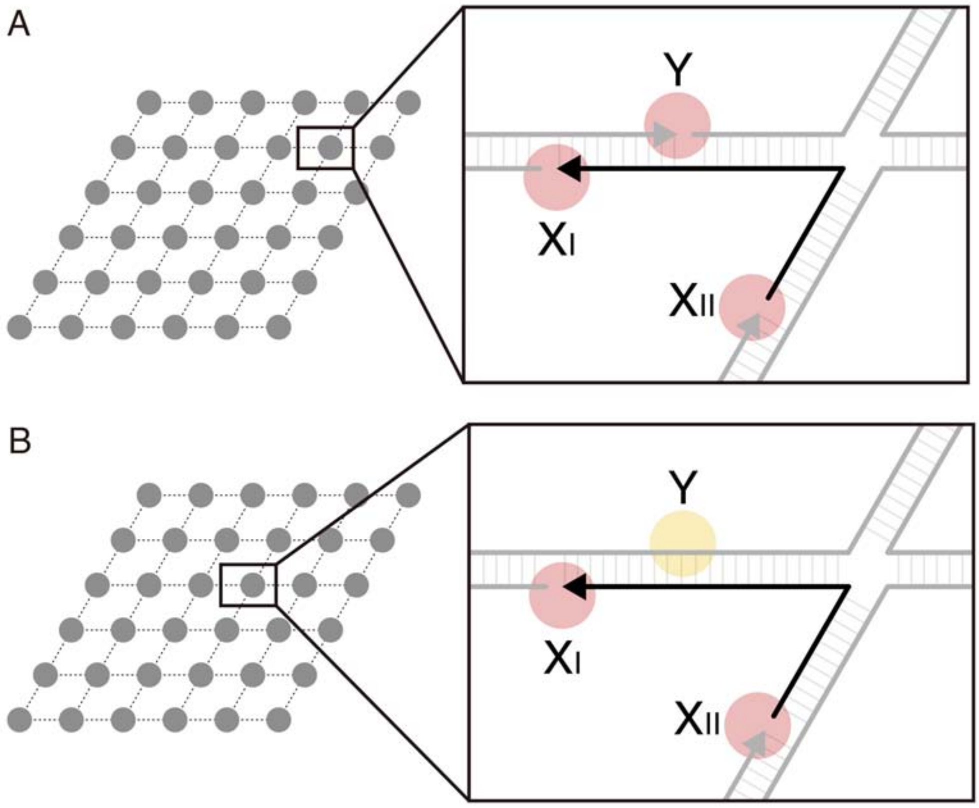

Figure S27. Schematics of representative ligation events based on 6 × 6 lattice. (A) Status 1 of the component strand with all three nicks X_I, X_{II} and Y unligated. (B) Status 2 of the component strand with nicks X_I, X_{II} unligated but nicks Y ligated.

According to our model, strands of status 1 would dissociate from the lattice, stands of status 3 would remain in the lattice, and strands of status 2 would partially dissociate from the lattice. When there is no any strand dissociated from the entire lattice, it is defined as an intact lattice. Detailed implementation can be found in the pseudocode in Figure S28. Code for simulation and analysis is provided as part of the public repository. It is available at https://github.com/Yunoinsky/DNA-lattices-simulation.

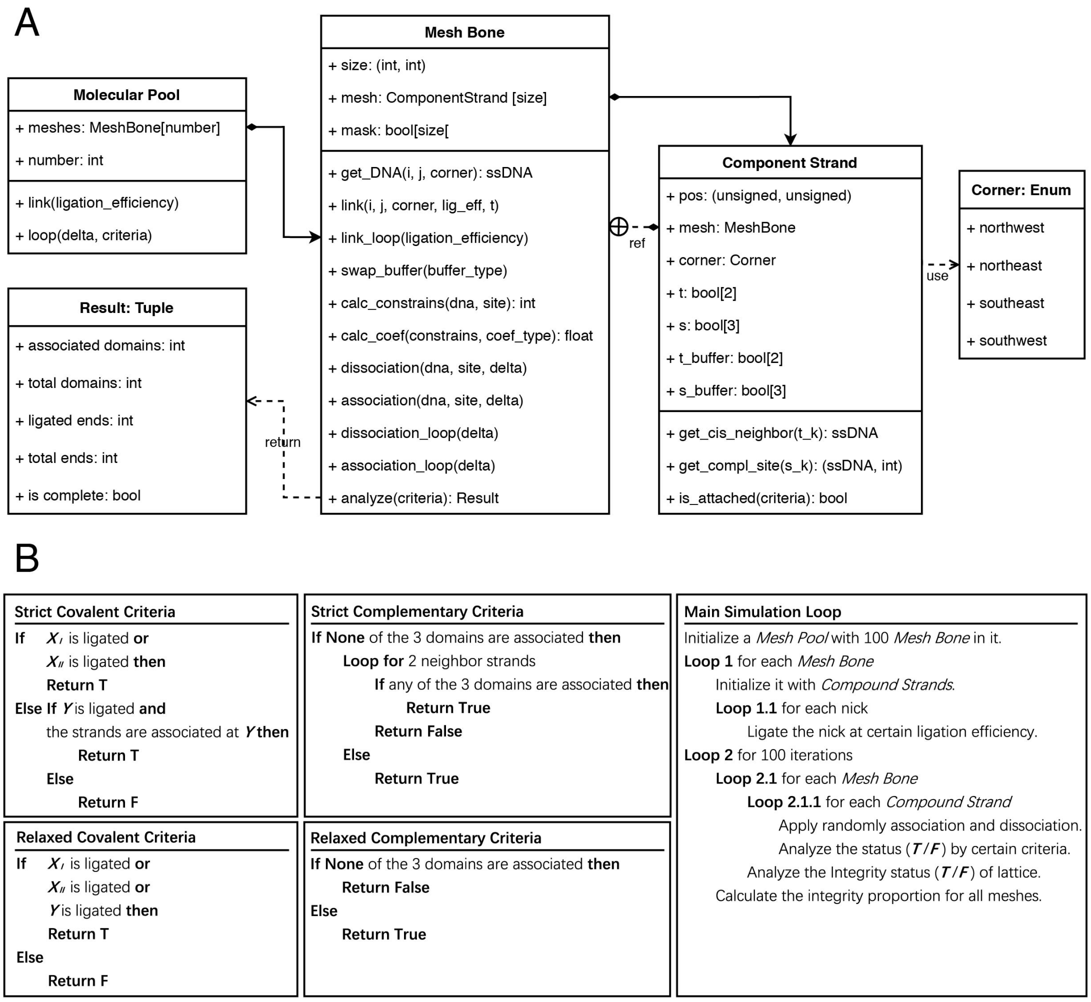

Figure S28. Construction and implementation of simulation system. (A) UML class diagram of simulation. Object-oriented programming paradigm is used to organize simulation data. Molecular Pools located on the top of the system handle all operations and analyze the total results. Mesh Bones represent lattices, in which component strands are assigned. They are functionalized as virtual component strands, which can be ligated, and then dissociate or associate in certain conditions. (B) The pseudocode of simulation programs and four criteria.

We simulated a batch of 100 lattices simultaneously in a molecular pool. 100 iterations were executed in each batch to reach equilibrium. At the end of each iteration, the proportion of intact lattices was analyzed. The elbow point was found at ~50th iteration, so we used the mean proportion of the last 50 iterations to represent the intact lattice proportion at equilibrium[2]. 100 batches were applied for each level of ligation efficiency in our simulation.

Simulation results based on 6 × 6 lattice with a center hole are also provided in Figure S29.

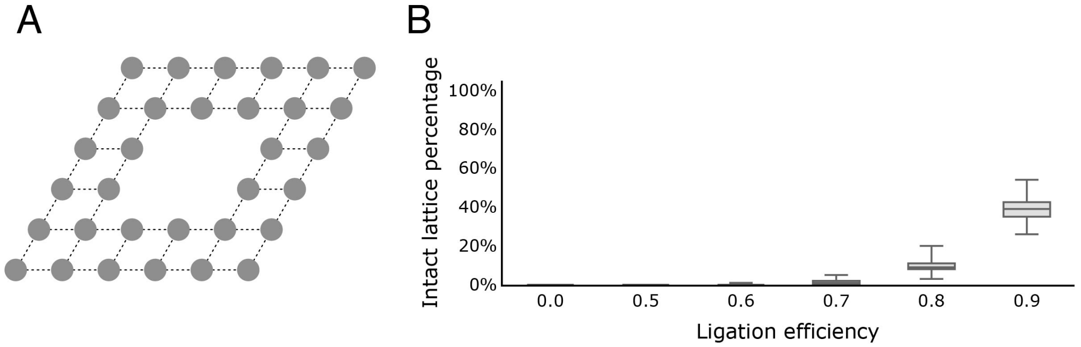

Figure S29. (A) Schematics of 6 × 6 lattice with a center hole. (B) Simulation results of intact lattice proportion.

S5 AGE results of structures without ligatable nicks

Another 6 × 6 wireframe lattice without ligatable nicks was also investigated by thermal denaturation test (Figure S30). Connectivity of motifs results in a hexagonal pattern (Figure S30, A and B). Nicks locate at the branching points instead of within continuous duplexes. Although we believe the 5' phosphate and 3' hydroxyl of such nicks are in proximity, they are not proper subjects of enzymatic ligation (i.e. unligatable nicks). Melting temperatures calculated based on melting curves of the lattices (Figure S30, C and D) are similar, 57 °C for unligated lattices and 54 °C for ligated lattices respectively.

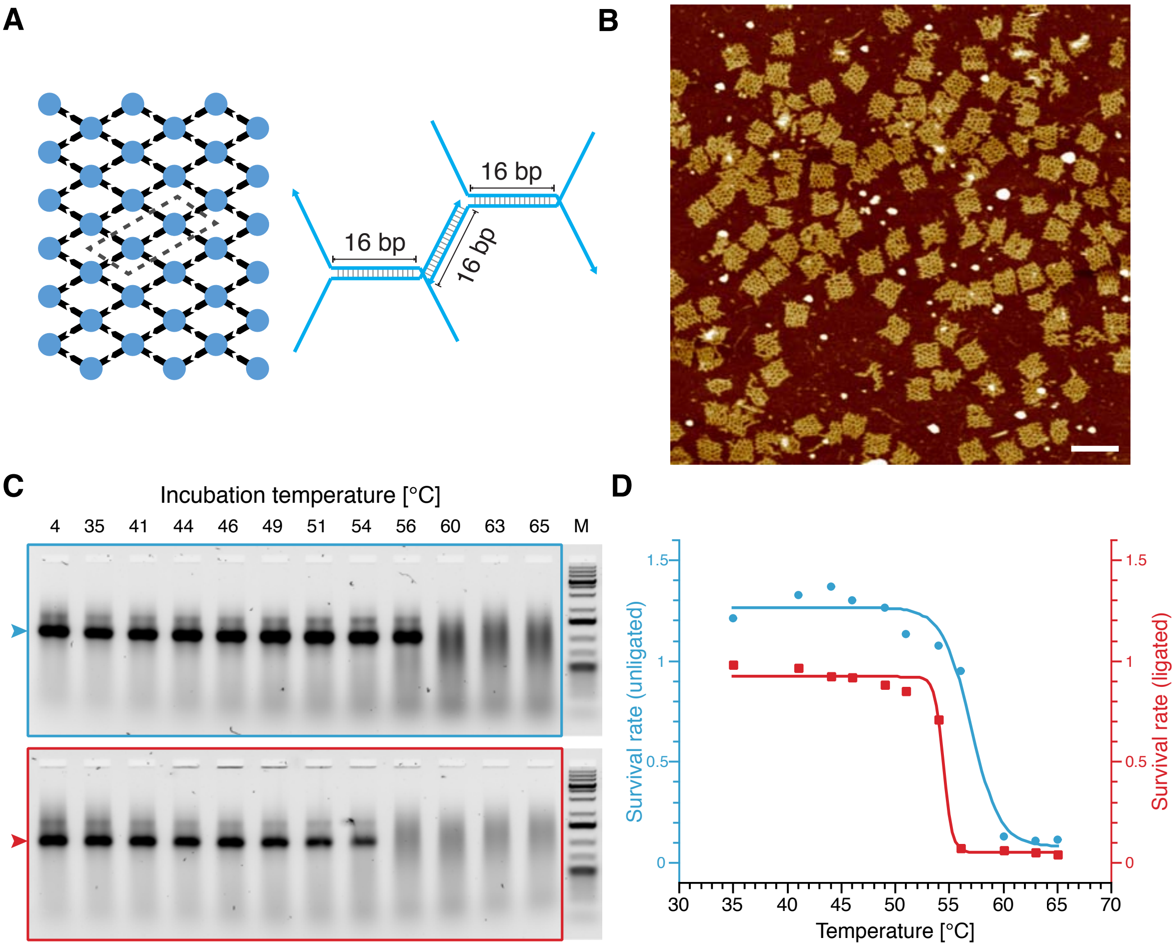

Figure S30. Thermal denaturation test results of a 6 × 6 lattice without ligatable nicks. (A) Schematics of the lattice (left) and motif design (right). (B) A representative AFM image of unligated lattices. Scale bar: 100 nm. (C) AGE results of lattices without (highlighted in blue box) and with (highlighted in red box) ligation treatment. Numbers above gel lanes indicate corresponding incubation temperatures. Arrows point at target structures. Lane M: 1-kb ladder. (D) Melting curves of unligated (blue) and ligated (red) lattices.

S6 Morphology investigation of mechanical stability

Although clear bands were observed in AGE results of control samples (stored at 4 °C) of lattice J4-II with and without ligation treatment (Figure S4, lane 4), the unligated lattices were mechanically fragile upon deposition on mica surface as shown in Figure S31A. Such vulnerability may be attributed to the poor pairing strength of the 6-bp stem domains. After ligation treatment, the structure integrity was substantially improved under AFM (Figure S31B), which is consistent with previous study[3].

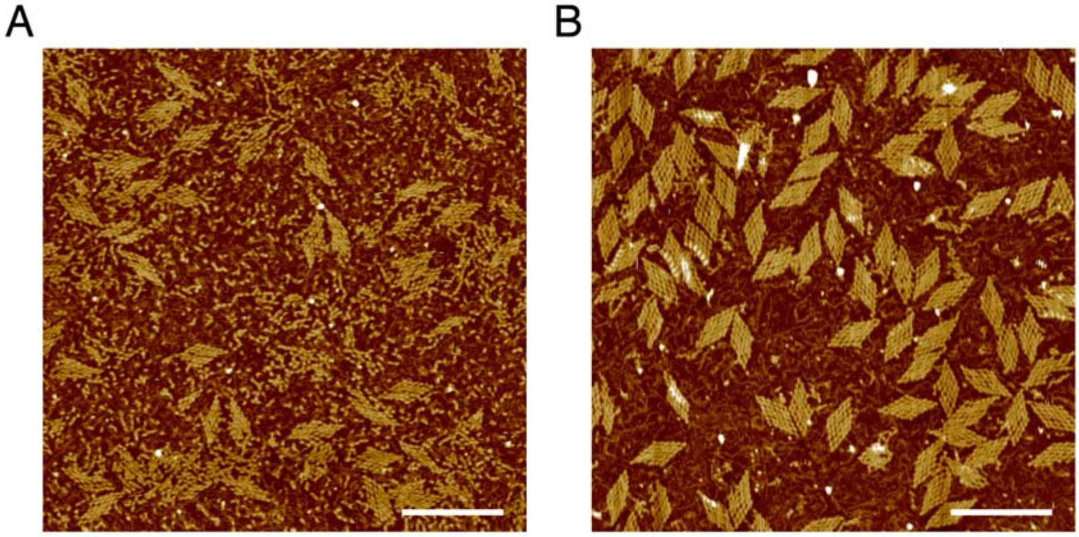

Figure S31. AFM images of lattice J4-II without (A) and with (B) ligation treatment. Scale bars: 200 nm.

S7 Dynamic structural reconfiguration by enzymatic ligation

S7.1 Loss of ligation function based structural reconfiguration

A lattice adapted from J4-I (J4-IA) was used to investigate dynamic structural reconfiguration by loss of ligation function (Figure S32 - Figure S35). Taking advantage of programmability and addressability of the J4-IA, we applied 5’ phosphorylation on the selective component strands of three J4-IA lattices, which is essential for successive ligation treatment. The three reconfigurable lattices consist of 96, 96 and 108 ligatable nicks (out of 120 nicks) and correspond to the three design patterns, loss of a corner (Figure S32A and Figure S33), loss of a center (Figure S32B and Figure S34), and splitting into halves (Figure S32C and Figure S35) respectively. AGE results showed substantial mobility difference between the reconfigured lattices and the original ones after ligation treatment and thermal incubation, indicating the dissociation of the unligatable segments and the enhanced thermal stability of the ligatable segments (highlighted by red arrows). AFM results further confirmed the designed morphologies after reconfiguration. The design of splitting into halves required slightly higher thermal treatment compared to the other two, which was assumed to correlate with the stronger stacking force between the ligatable and unligatable segments.

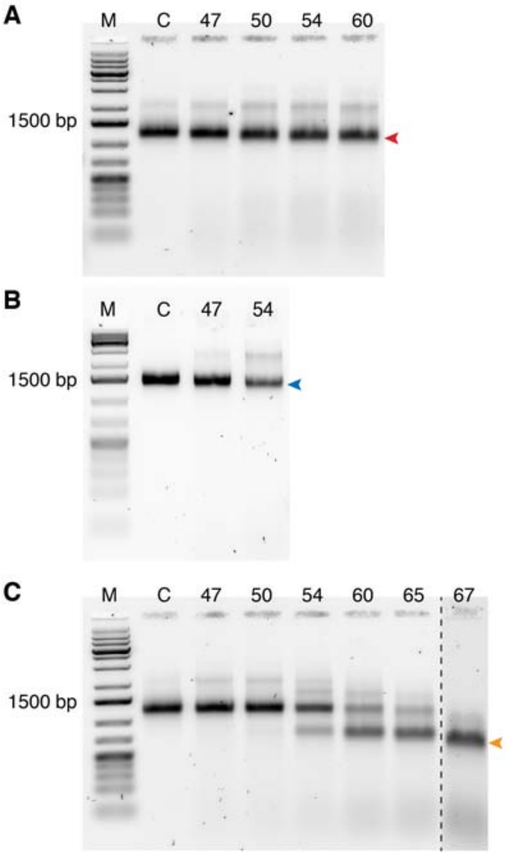

Figure S32. AGE results of J4-IA of different numbers of ligatable nicks after ligation treatment and thermal incubation. (A) J4-IA of loss of a corner. Red arrow points at the target band of ligated J4-IA of loss of a corner. Corresponding AFM image is shown in Figure S33B. (B) J4-IA of loss of a center. Blue arrow points at the target band of ligated J4-IA of loss of a center. Corresponding AFM image is shown in Figure S34B. (C) J4-IA of splitting into halves. Orange arrow points at the target band of ligated J4-IA of splitting into halves. Corresponding AFM image is shown in Figure S35B. Numbers above gel lanes indicate corresponding incubation temperatures. Lane M: 1-kb ladder.

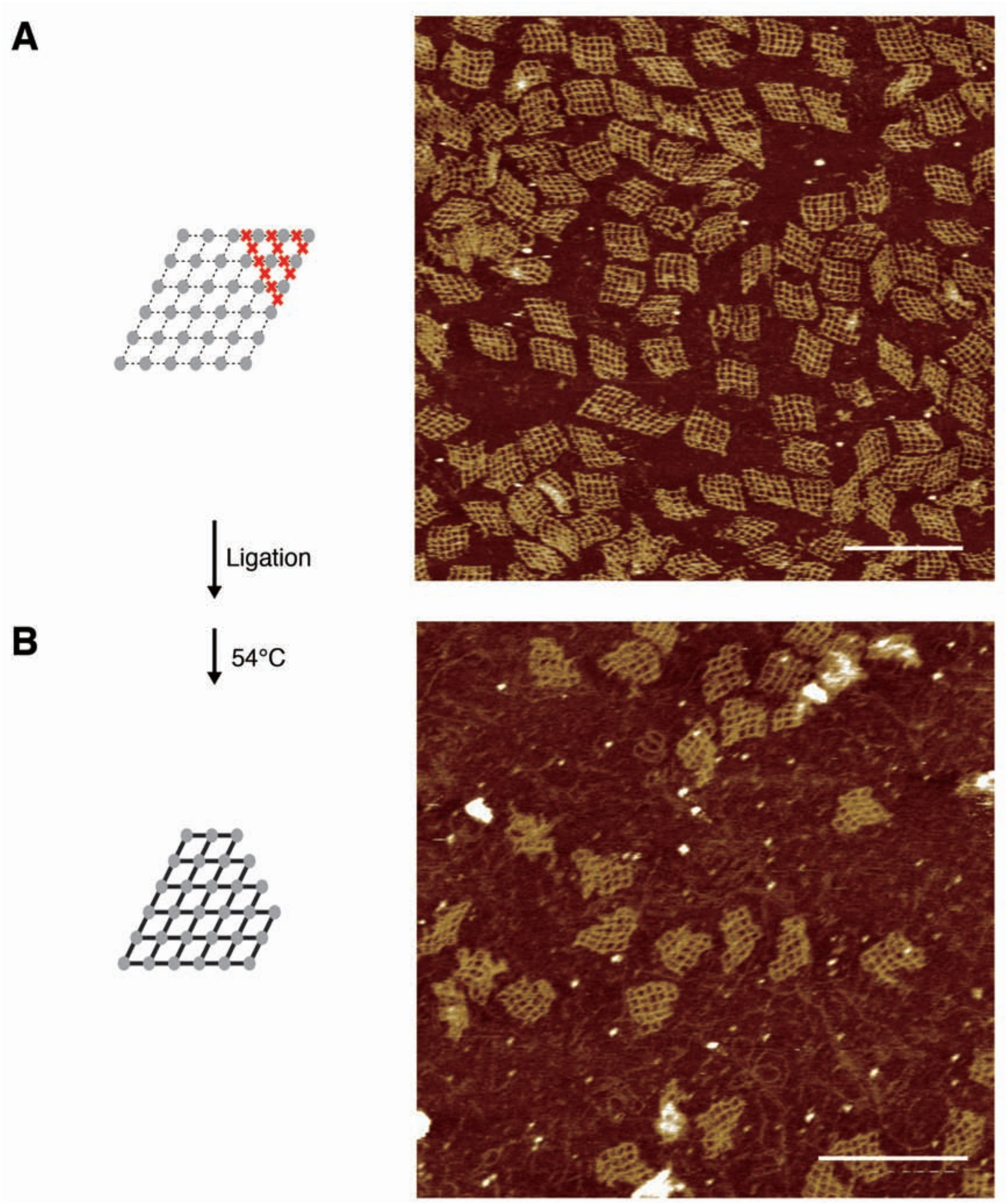

Figure S33. Full-size AFM images of dynamic reconfiguration of lattice J4-IA of loss of a corner design. J4-IA lattice before (A) and after (B) ligation treatment and thermal incubation. Left: schematics of the structures. Red cross checks represent unligatable nicks. Right: AFM images in correspondence with lane C (top) and lane 60 (bottom) of gel results in Figure S32A respectively. Scale bars: 200 nm.

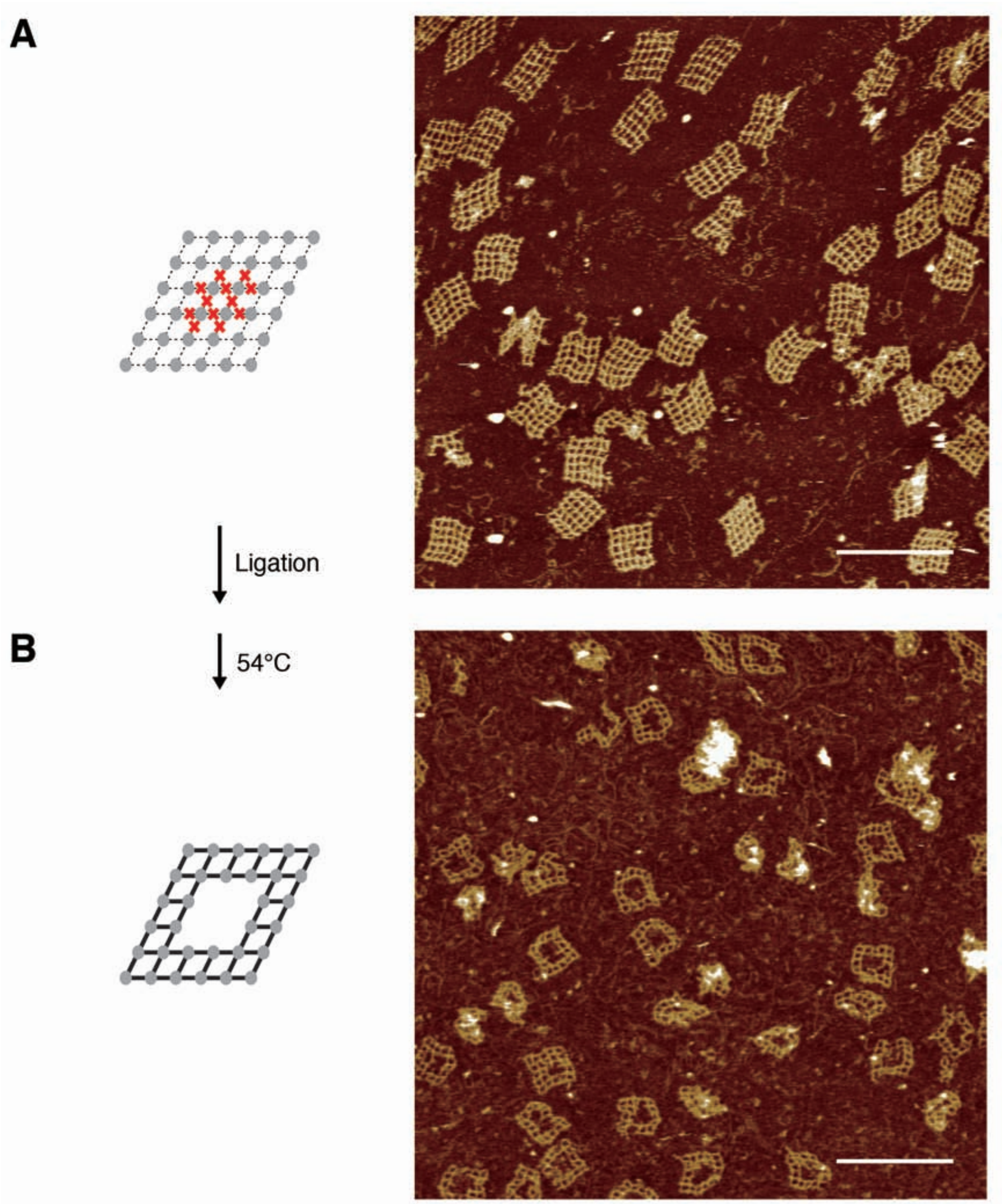

Figure S34. Full-size AFM images of dynamic reconfiguration of lattice J4-IA of loss of a center design. J4-IA lattice before (A) and after (B) ligation treatment and thermal incubation. Left: schematics of the workflow. Red cross checks represent unligatable nicks. Right: AFM images in correspondence with lane C (top) and lane 54 (bottom) of gel results in Figure S32B respectively. Scale bars: 200 nm.

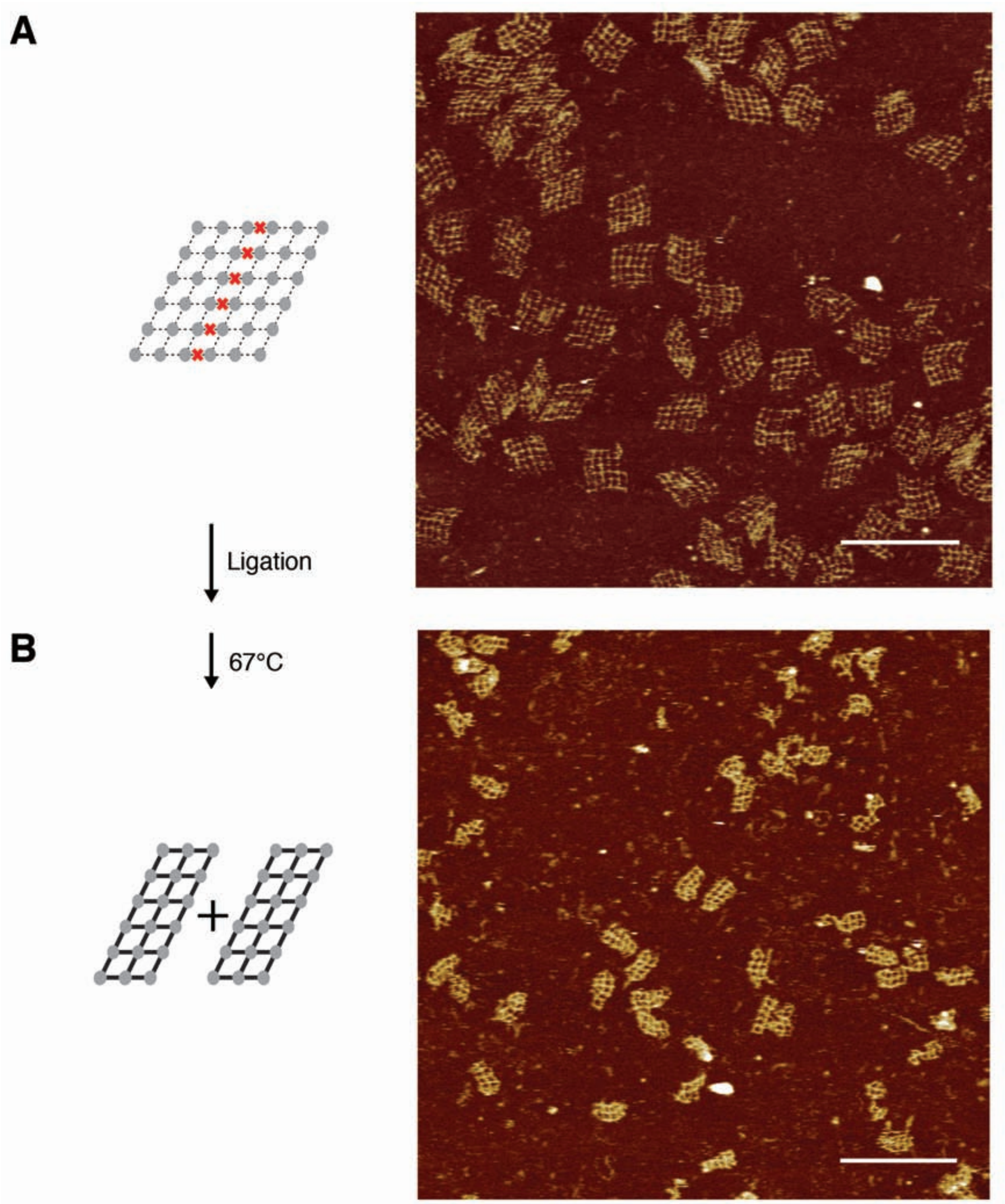

Figure S35. Full-size AFM images of dynamic reconfiguration of lattice J4-IA splitting into halves design. J4-IA lattice before (A) and after (B) ligation treatment and thermal incubation. Left: schematics of the workflow. Red cross checks represent unligatable nicks. Right: AFM images in correspondence with lane C (top) and lane 67 (bottom) of gel results in Figure S32C respectively. Scale bars: 200 nm.

S7.2 Gain of ligation function based structural reconfiguration

A lattice adapted from J4-I (J4-IB) was used to investigate dynamic structural reconfiguration by gain of ligation function (Figure S36 - Figure S38). We designed 3-nt complementary sticky end side overhangs on the east and west side of the lattice. As gain of ligation function, the transient sticky end cohesion was converted into a permanent one and in turn the planar structure reconfigured into a tubular configuration. Both AGE and AFM results confirmed the ligation-induced structural reconfiguration.

Before ligation, 3-nt sticky end side overhangs kept the lattice as a planar sheet after self-assembly (Figure S36A; lane 2 in Figure S37A). AGE results (lanes 1 and 2 in Figure S37A) showed the same mobility for a planar sheet lattice with non-complementary TTT side overhangs (T3) and the unligated lattice of 3-nt sticky end side overhangs (S3U), pointing to the planar configuration of the lattice S3U in solution. AFM results also confirmed the planar sheet morphology of the lattices T3 (Figure S37B) and S3U (Figure S36A and Figure S37C).

Upon ligation, the planar lattice reconfigured into a tubular shape (Figure S36B; lane 3 in Figure S37A). AGE results (lanes 2 and 3 in Figure S37A) showed the mobility difference between the lattice before (S3U) and after (S3L) ligation treatment, which indicated the ligation-induced reconfiguration of the lattice into a tubular shape. Similar mobility was also observed for the ligated structure (lane 3 in Figure S37A) and the tubular structures of 10-nt sticky ends (lanes 4 and 5 in Figure S37A), which further confirmed the tubular shapes of the lattice S3L. The mobility of the tubular structures of 10-nt sticky ends (lanes 4 and 5) remained the same before (S10U) and after (S10L) ligation, suggesting limited structural impact of ligation treatment for a well-defined tubular structure. AFM results were consistent with the AGE results and verified the tubular morphology of the lattice S3L (Figure S36B and Figure S37D) as well as the S10U and S10L (Figure S37, E and F)

When the side overhangs were intentionally prepared as unligatable, a planar configuration remained after ligation treatment (Figure S38), which further validated the induction role of ligation treatment on the sheet-to-tube reconfiguration.

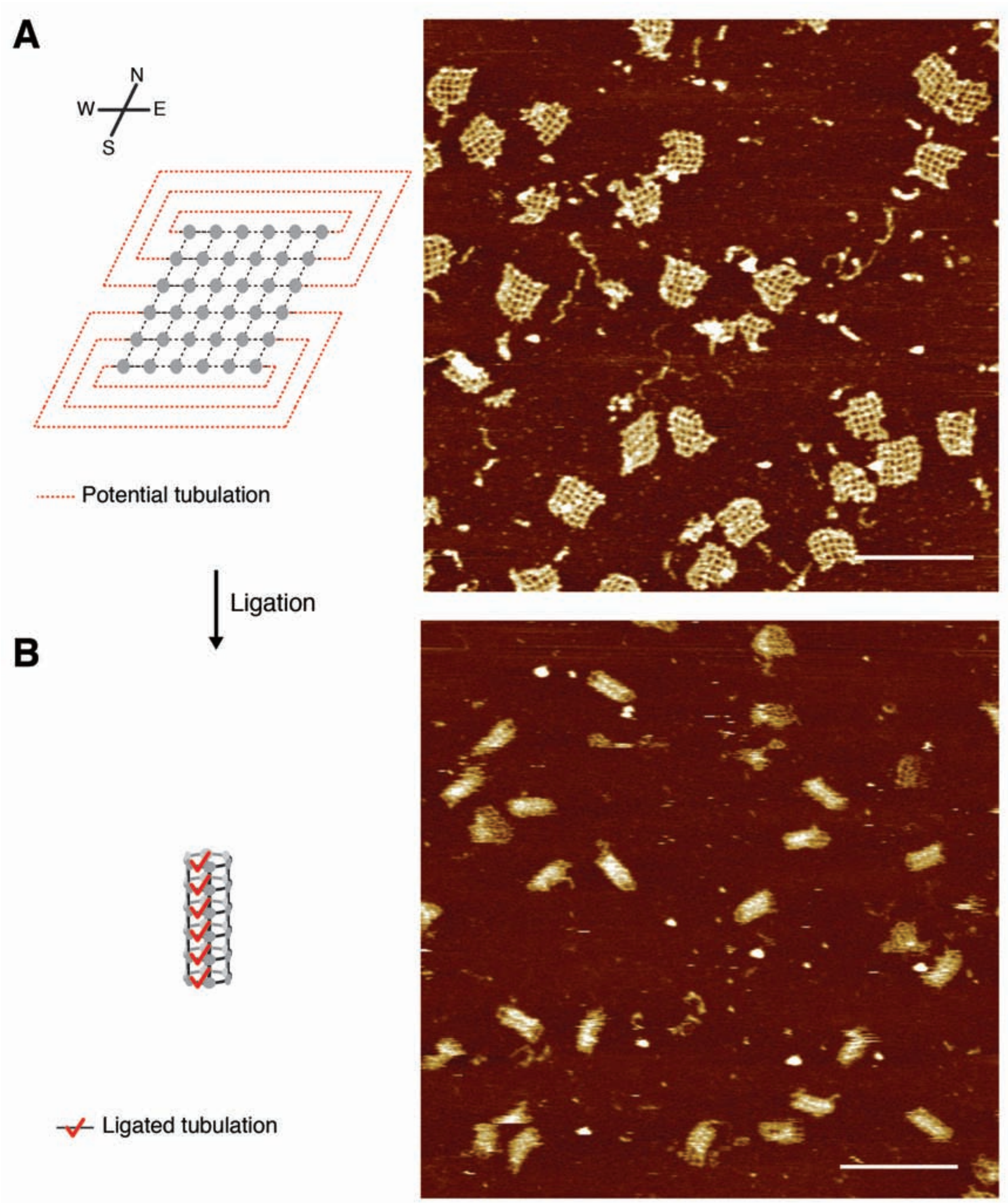

Figure S36. Full-size AFM images of dynamic reconfiguration of lattice J4-IB. Lattice J4-IB of 3-nt complementary sticky end side overhangs before (A) and after (B) ligation treatment. Dashed red ticks and dashed lines represent edges of potential tubulation. Solid red ticks and lines represent newly assembled edges after successful tubulation by ligation treatment. Left: schematics of the workflow. Right: AFM images in correspondence with lane 2 (top) and lane 3 (bottom) of gel results in Figure S37A respectively. Scale bars: 200 nm.

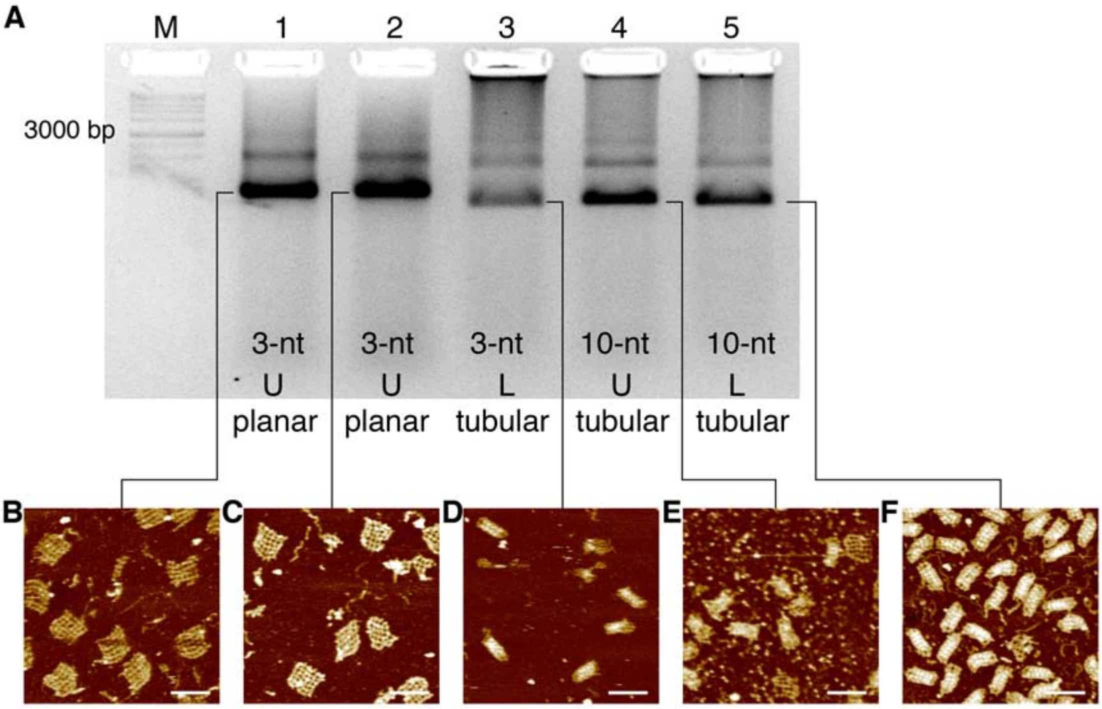

Figure S37. AGE and AFM results of ligation-induced reconfiguration of lattice J4-IB. (A) AGE results. Lane M: 1-kb ladder. Lane 1: A planer sheet lattice of non-complementary TTT side overhangs (T3), corresponding AFM image shown in Figure S37B. Lane 2: Unligated lattice J4-IB (planar) of 3-nt complementary sticky end side overhangs (S3U), corresponding AFM images shown in Figure S36A and Figure S37C. Lane 3: Ligated lattice J4-IB (tubular) of 3-nt complementary sticky end side overhangs (S3L), corresponding AFM images shown in Figure S36B and Figure S37D. Lane 4: A tubular structure of 10-nt complementary sticky ends before ligation (S10U), corresponding AFM image shown in Figure S37E. Lane 5: A tubular structure of 10-nt complementary sticky ends after ligation (S10L), corresponding AFM image shown in Figure S37F. U: unligated samples. L: ligated samples. (B-F) AFM results. AFM images are in correspondence with lanes 1-5 in A respectively. Scale bars: 100 nm.

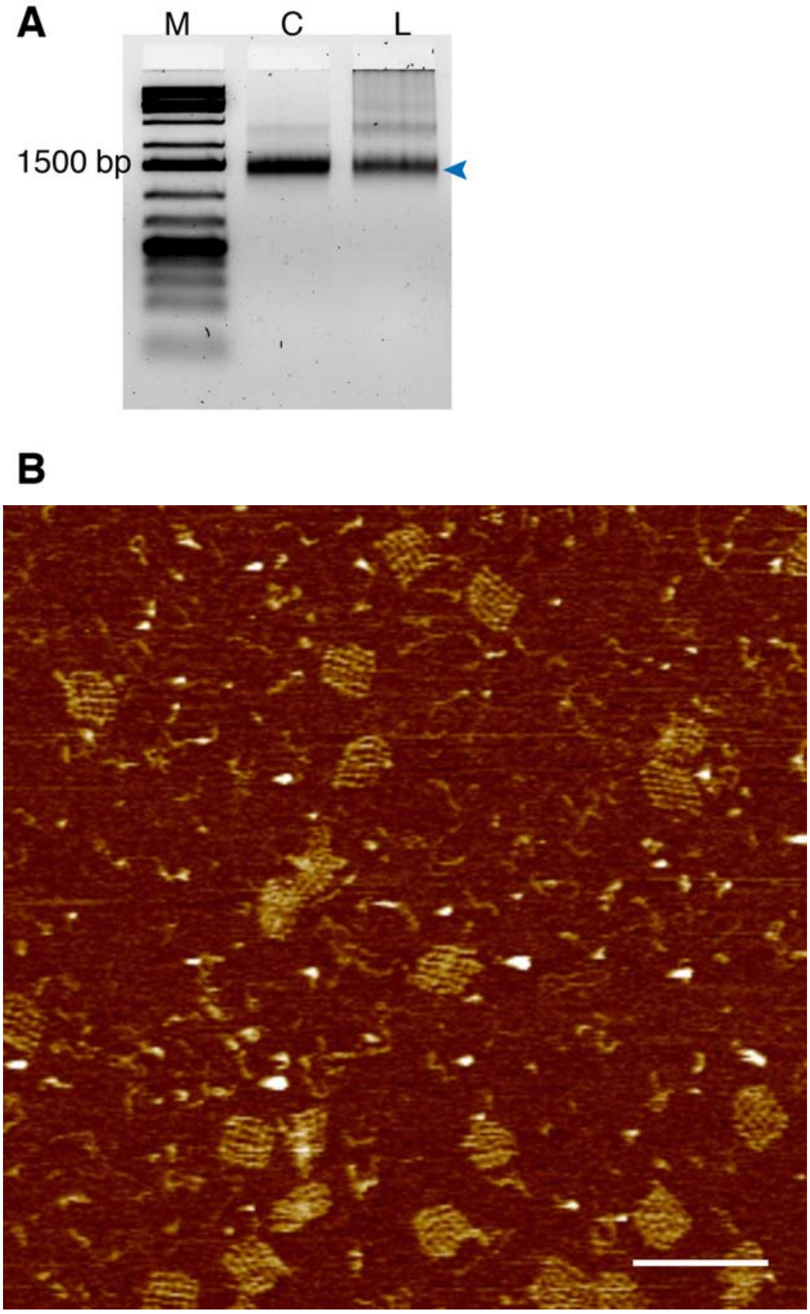

Figure S38. AGE and AFM results of lattice J4-IB of unligatable 3-nt complementary sticky end side overhangs. (A) AGE result. Lane M: 1-kb ladder. Lane C: samples before ligation treatment. Lane L: samples after ligation treatment. Blue arrows point at target band of ligated J4-IB of unligatable 3-nt complementary sticky ends (planar). (B) AFM result. AFM image of lattice J4-IB of unligatable 3-nt sticky ends after ligation, in correspondence with lane L in A. Scale bars: 200 nm.

References

1 O'Neill, P., Rothemund, P. W., Kumar, A. & Fygenson, D. K. Sturdier DNA nanotubes via ligation. *Nano Lett* **6**, 1379-1383, doi:10.1021/nl0603505 (2006).

2 Vologodskii, A. & Frank-Kamenetskii, M. D. DNA melting and energetics of the double helix. *Physics of Life Reviews* **25**, 1-21, doi:10.1016/j.plrev.2017.11.012 (2018).

3 Wang, W. *et al.* Self-assembly of fully addressable DNA nanostructures from double crossover tiles. *Nucleic Acids Research* **44**, 7989-7996, doi:10.1093/nar/gkw670 (2016).

J4-I(11bp10bp)	
NAME	SEQUENCE
J4-I-1	CCTTCAAGGGCCCAAGACAGACTTTTTTTTT
J4-I-2	GTCTGTCTTGGCAGTAAGAGCACGTCAGCCAG
J4-I-3	CATAGAATCCAGCCCTTGAAGGTTTTTTTTT
J4-I-4	TGTTGGTGTAGAAAGAGAGAACTGTATATCAT
J4-I-5	GTGGGCCGCGGACCAGGTTCATTTTTTTTTT
J4-I-6	ATGAACCTGGTTTACGACTGACTAATATAACA
J4-I-7	AGGGCGGGCGGCCGCGGCCCACCTGGCTGACG
J4-I-8	TGCTCTTACTGTGGATTCTATGATGATATACA
J4-I-9	GTTCTCTCTTTAGCTCCCAAACCTTGCCTTTA
J4-I-10	AACTGCATGGACTACACCAACATTTTTTTTT
J4-I-11	ATGCTTGAATTTACAAACTATTTGACCATCCA
J4-I-12	GGGACATGGATTGTTTGACCTCAATGCATACG
J4-I-13	GATCAACGCATGTGACCTTAATTTTTTTTTT
J4-I-14	ATTAAGGTCACAGCAACTTAGCTACTTTGGAG
J4-I-15	TTGAACTTCATATGCGTTGATCTGTTATATTA
J4-I-16	GTCAGTCGTAACCGCCCGCCCTCGTATGCATT
J4-I-17	GAGGTCAAACAGAGTCCGCTATGTTTGGCCCT
J4-I-18	GCCATGGTTTCATCCATGTCCCTAAAGGCAAG
J4-I-19	GTTTGGGAGCTTCCATGCAGTTTGGATGGTCA
J4-I-20	AATAGTTTGTAATTCATCCGTCAGCCGTTGCT
J4-I-21	CACGGCAGTCAAATTCAAGCATTTTTTTTTT
J4-I-22	GGGACATAGCGCCTATGTCCGTAGCCCTCGGT
J4-I-23	GTCAGACATGGCGACTGAAGCGACCCGTGTTC
J4-I-24	ATGTTGGTGAGAAGATAGTCGGACGGTGTGGA
J4-I-25	GGATCATCGCATCAGTAGACTCTTTTTTTTT
J4-I-26	GAGTCTACTGAAGGAGGATCGAAGGGCGCGGT
J4-I-27	CTCTCGTGGGCTGCGATGATCCCTCCAAAGTA
J4-I-28	GCTAAGTTGCTATGAAGTTCAATCCACACCGT
J4-I-29	CCGACTATCTTAAGATGGGCCGACGTGAACAT
J4-I-30	GCATTTAGATCCTCACCAACATAGGGCCAAAC
J4-I-31	ATAGCGGACTCGAAACCATGGCGAACACGGGT
J4-I-32	CGCTTCAGTCGGCCACATCCTTCGATTCCCGT
J4-I-33	CAAGTGAATAACCATGTCTGACAGCAACGGCT
J4-I-34	GACGGATGAATTGACTGCCGTGACCGAGGGCT
J4-I-35	ACGGACATAGGCCCTTGCCGACTATGGGCTCG
J4-I-36	CACTCAGATGGCGCTATGTCCCTTTTTTTTT
J4-I-37	GGCTCGGTAGTATTGCGCTTTCACCGTCAATT
J4-I-38	ACCGTTGCTTTATCCTGCGAGCACCCTGTCTG
J4-I-39	TAAAGTTCCCTTGTATATGTTTATAGCACGTA
J4-I-40	AACCCGCGTACTGCTCTACACTTTGTCAATTA
J4-I-41	GTAAAGATATTCCGCGACGGTTTTTTTTTTT
J4-I-42	AACCGTCGCGGATCTTGCAACGTTGCTTGCAG
J4-I-43	CGAGCGGTTAGAATATCTTTACACCGCGCCCT

J4-I-44	TCGATCCTCCTGCCCACGAGAGTAATTGACAA
J4-I-45	AGTGTAGAGCAGAATTCCTCGGTTTCGTTCCG
J4-I-46	TCACACGTCCAGTACGCGGGTTATGTTCACGT
J4-I-47	CGGCCCATCTTGATCTAAATGCTACGTGCTAT
J4-I-48	AAACATATACATCCCTTACCAGGTCGACGTCG
J4-I-49	GGCGCGCCAACAGGGAACTTTAACGGGAATCG
J4-I-50	AAGGATGTGGCTTATTCACTTGCAGACAGGGT
J4-I-51	GCTCGCAGGATAATTCAGGTCAACGCAGGTCA
J4-I-52	CGGGCTTTAACAAAGCAACGGTCGAGCCCATA
J4-I-53	GTCGGCAAGGGCCATCTGAGTGAATTGACGGT
J4-I-54	GAAAGCGCAATGGGATGCCGCTCGTTCCGGCT
J4-I-55	AGTTGTCTCCTACTACCGAGCCTTTTTTTTTT
J4-I-56	AGATTTGCAGTTGTGGGCGATGAGCACCACCG
J4-I-57	CAGGGAATGGACTGTCGCATTAGCTCCAGTGA
J4-I-58	TCTCACCGCACTGGACAAATTTAGGCCTGCAG
J4-I-59	CTCGGCGAGACTCAGAACCCATGTTCCCGATC
J4-I-60	GCTAAATGGCTTTCCATGAAGTTATATGCTAC
J4-I-61	CGTCTTTATATTCCGACGTAAGTTTTTTTTTT
J4-I-62	CTTACGTCGGACTATGTAATATTTTTTTTTTT
J4-I-63	TCAACGAGGACATATAAAGACGCTGCAAGCAA
J4-I-64	CGTTGCAAGATCTAACCGCTCGGTAGCATATA
J4-I-65	ACTTCATGGAATGTTCTTCGCAGTCGACGGAG
J4-I-66	TAAAGTAGAGTAGCCATTTAGCCGGAACGAAA
J4-I-67	CCGAGGAATTCTGGACGTGTGAGATCGGGAAC
J4-I-68	ATGGGTTCTGATCTGTAAAGACGCTAGGAGCA
J4-I-69	TGCTTCCCTCAGTCTCGCCGAGCGACGTCGAC
J4-I-70	CTGGTAAGGGAGTTGGCGCGCCCTGCAGGCCT
J4-I-71	AAATTTGTCCACACCTAGCTACTGGCGGGCAC
J4-I-72	TGAAACGATGTGTGCGGTGAGATGACCTGCGT
J4-I-73	TGACCTGAATTGTTAAAGCCCGTCACTGGAGC
J4-I-74	TAATGCGACAGATGGGCTTTAAGGCTATCGTA
J4-I-75	TTAACACCTGCTCCATTCCCTGAGCCGGAACG
J4-I-76	AGCGGCATCCCAGGAGACAACTCGGTGGTGCT
J4-I-77	CATCGCCCACAACGCTGAATAGCGCTGGAGTC
J4-I-78	GTCACGAACTAACTGCAAATCTTTTTTTTTTT
J4-I-79	GTTAACAGATCTGGTGTGGTATGATGTTGTGG
J4-I-80	TCGTCCACGTGTTCAATCTGATGGTTAACGCT
J4-I-81	AATGGTACAACGGTTAGCCGCGGCTATATTGG
J4-I-82	CGGAATGATAGGTCGGTTACTGGTAGGCAGTA
J4-I-83	GATCCCAACCGCCCTCCCTAAATCGTCAAACA
J4-I-84	ATATTACATAGGTCCTCGTTGATGTTTGACGA
J4-I-85	TTTAGGGAGGGCTAGTCAGAGATTTTTTTTTT
J4-I-86	GTTAGCTGTGCCGGTTGGGATCCTCCGTCGAC
J4-I-87	TGCGAAGAACAACTCTACTTTATACTGCCTAC
J4-I-88	CAGTAACCGACACGGTCCGCAGGTCAGTATCA

J4-I-89	CGGGCAGAGAGCTATCATTCCGTGCTCCTAGC
J4-I-90	GTCTTTACAGATGAGGGAAGCACCAATATAGC
J4-I-91	CGCGGCTAACCCTACTTGATATTCGGATAGAT
J4-I-92	TCCGGCTCGAAGTTGTACCATTGTGCCCGCCA
J4-I-93	GTAGCTAGGTGACATCGTTTCAAGCGTTAACC
J4-I-94	ATCAGATTGAAGGGCAAAGACCATGGTGATTC
J4-I-95	TATATTCCTGCCACGTGGACGATACGATAGCC
J4-I-96	TTAAAGCCCATGCAGGTGTTAACCACAACATC
J4-I-97	ATACCACACCACGTGCTAGCCACTGACCAGCT
J4-I-98	CTTGGTGACGGGATCTGTTAACGACTCCAGCG
J4-I-99	CTATTCAGCGTTAGTTCGTGACTTTTTTTTTT
J4-I-100	GTCGCTGCAAATTGTGTTGTATAATATGTCAG
J4-I-101	TCCCGTTGTCGTCGGCATGCTATCCCTCGAAG
J4-I-102	TCAGATGCCGATGATGGGTACGGTGCTCTAGC
J4-I-103	GCATGGCCCTCTACCGCCTTCGGAACAGTGGT
J4-I-104	TCTCTGACTAGGCACAGCTAACACCACTGTTC
J4-I-105	CGAAGGCGGTATGATCTGGTCGTTTTTTTTTT
J4-I-106	AGTTGCTGGTGGAGGGCCATGCTGATACTGAC
J4-I-107	CTGCGGACCGTCTCTCTGCCCGGCTAGAGCAC
J4-I-108	CGTACCCATCATCATCCACTCCTTGCTTAATG
J4-I-109	ATCTTACAAGTTCGGCATCTGAATCTATCCGA
J4-I-110	ATATCAAGTAGTTCGAGCCGGACTTCGAGGGA
J4-I-111	TAGCATGCCGAATTTATAGGGCGCCTCTGATG
J4-I-112	CGCCCTACATACGACAACGGGAGAATCACCAT
J4-I-113	GGTCTTTGCCCGCAGGAATATACTGACATATT
J4-I-114	ATACAACACAATGCTGTGCCCTCAAAGGTATC
J4-I-115	GCATATAGCGGTTTGCAGCGACAGCTGGTCAG
J4-I-116	TGGCTAGCACGCCGTCACCAAGTTTTTTTTTT
J4-I-117	AACCCTGCTATTAATTGCGCAGTGTTTAGGAT
J4-I-118	ACCTGCGGATATCAAGAGGGCTTTGAGCTACC
J4-I-119	GTTCGATGTCTAGTTCGAACAGTTGGAAGGTC
J4-I-120	CGACCAGATCACACCAGCAACTGACCTTCCAA
J4-I-121	CTGTTCGAACTCGGTGCCCTGTTTTTTTTTTT
J4-I-122	CCTGTCTAGCAAGACATCGAACCATTAAGCAA
J4-I-123	GGAGTGGATGAACTTGTAAGATGGTAGCTCAA
J4-I-124	AGCCCTCTTGACTGAACATGGCCCTTCGTCCG
J4-I-125	TACTACAAATATATCCGCAGGTCATCAGAGGC
J4-I-126	GCCCTATAAATTATGTAGGGCGATCCTAAACA
J4-I-127	CTGCGCAATTACCCTATATGCATCTAGTAAGT
J4-I-128	CATCCATGATGATAGCAGGGTTGATACCTTTG
J4-I-129	AGGGCACAGCACCGCTATATGCTTTTTTTTTT
J4-I-130	TCCGACGTTACATGGTCCCGCGGGCGGCACGT
J4-I-131	CGCGCGACATAATTCGGTATCTGTACTCAAAG
J4-I-132	ACAGGGCACCGTGCTAGACAGGCTTTGAGTAC
J4-I-133	AGATACCGAATCTCTGGTTGAATTTTTTTTTT

J4-I-134	CGTTGAAGTTATATGTCGCGCGGACGAAGG
J4-I-135	GCCATGTTCAGTATTTGTAGTAACGTGCCGCC
J4-I-136	CGCGGGACCATGCAGGACTGGACATGTAGCGA
J4-I-137	CCTACCGGGAAGTAACGTCGGAACTTACTAGA
J4-I-138	TGCATATAGGGCATCATGGATGTTTTTTTTTT
J4-I-139	AGGGCTTTATTCGTAAATAGAGTGGACCTGTA
J4-I-140	TTCAACCAGAGTAACTTCAACGTACAGGTCCA
J4-I-141	CTCTATTTACGTGAAGCGATGCTTTTTTTTTT
J4-I-142	GTAACTAATAGAATAAAGCCCTTCGCTACATG
J4-I-143	TCCAGTCCTGCTTCCCGGTAGGTTTTTTTTTT
J4-I-144	GCATCGCTTCACTATTAGTTACTTTTTTTTTT

J4-II(13bp6bp)	
NAME	SEQUENCE
J4-II-1	TGCCCTGTAAACACTCTATCGCGATTTTTTTT
J4-II-2	AATCGCGATAGAGGGTCAATAAAGTCGTACCT
J4-II-3	AAATGATACTCTATGTTTACAGGGCATTTTTT
J4-II-4	GGTTGCTACTCCAGGAGGCCGACGTTCCCGAG
J4-II-5	ATCCTGAGTGGAAAGCTAATGCGGCTTTTTTT
J4-II-6	AGCCGCATTAGCTGTCTGTGATATATGAGTTT
J4-II-7	GCATGTTGCTCTGTTCCACTCAGGATAGGTAC
J4-II-8	GACTTTATTGACCTAGAGTATCATTTCTCGGG
J4-II-9	AACGTCGGCCTCCCTGATCCATTTGTGATACG
J4-II-10	TGCGGCCTTTGGATGGAGTAGCAACCTTTTTT
J4-II-11	TATAGTATGCGCTACCAAAGTGGGTCTGGCCT
J4-II-12	TTAAGGTCTTTCGGCCTCCCTTCATCACATGT
J4-II-13	CTAACAAATTGAGTTTAACCTGTTGATTTTTT
J4-II-14	TCAACAGGTTAAACAAGATATTGTCTGTGTAT
J4-II-15	GGTGAACGTGAGACTCAATTTGTTAGAAACTC
J4-II-16	ATATATCACAGACCAGAGCAACATGCACATGT
J4-II-17	GATGAAGGGAGGCATCTTTGGCTATTTATCCT
J4-II-18	CGAGTGTCCGAACCGAAAGACCTTAACGTATC
J4-II-19	ACAAATGGATCAGTCCAAAGGCCGCAAGGCCA
J4-II-20	GACCCACTTTGGTTGAAAGCTGACTTAAATTG
J4-II-21	ACCATGAAGCATTAGCGCATACTATATTTTTT
J4-II-22	CCCTCTGAACACTCAGAAGCATCTCTAAATCC
J4-II-23	GCATCGGGAATAGCGGTATTATCACGGAAGAT
J4-II-24	AAGTCTGAAGCAAACGGGCTCACGGATTCCCT
J4-II-25	CTGTCTCATGCGTTCAAACGTTGGATTTTTTT
J4-II-26	ATCCAACGTTTGAGTGTCCTTGGTTCAAGTGG
J4-II-27	ATGCTAGGTGCGAACGCATGAGACAGATACAC
J4-II-28	AGACAATATCTTGTCTCACGTTCACCAGGGAA
J4-II-29	TCCGTGAGCCCGTTATCATGGCCGGTTTAATG
J4-II-30	AGAGTCTACCGCGTTGCTTCAGACTTAGGATA
J4-II-31	AATAGCCAAAGATGTTCGGACACTCGATCTTC
J4-II-32	CGTGATAATACCGTTGGAGCGCCGCATCATTT
J4-II-33	TAGACACGCCAATCTATTCCCGATGCCAATTT
J4-II-34	AAGTCAGCTTTCAAATGCTTCATGGTGGATTT
J4-II-35	AGAGATGCTTCTGCTCGAAAGACACGCCTCCT
J4-II-36	TTAGAATTCCAGTAGTGTTCAGAGGGTTTTTT
J4-II-37	GCGCTTTGAGAACTTGTCTTTGTGTGCAATAT
J4-II-38	AATAACAAGGGAGTCGAACTAGAAACGGGTTG
J4-II-39	CTCGATTGCCGAACCTCCGTAGGACATTCAGT
J4-II-40	AGGCGATATTGCTACCAGCTTGGTCTATTAGT
J4-II-41	TAGCCCATCAGAGCTCTATCTTCGCATTTTTT
J4-II-42	TGCGAAGATAGAGTGCGACTTATTCGAAACTG
J4-II-43	GACCTGAGGGTGGCTCTGATGGGCTACCACTT

J4-II-44	GAACCAAGGACACTCGCACCTAGCATACTAAT
J4-II-45	AGACCAAGCTGGTGGGCGGGCGAACTTCCGTG
J4-II-46	GCCCAATACAGTCAGCAATATCGCCTCATTAA
J4-II-47	ACCGGCCATGATACGCGGTAGACTCTACTGAA
J4-II-48	TGTCCTACGGAGGTTGCTACGTCAATGGAGTG
J4-II-49	GTGGGCTGTTCCTTTCGGCAATCGAGAAATGA
J4-II-50	TGCGGCGCTCCAAATTGGCGTGTCTACAACCC
J4-II-51	GTTTCTAGTTCGAACCCATTAGGTAAGAAACA
J4-II-52	CTAAAGCAGTTTCCTCCCTTGTTATTAGGAGG
J4-II-53	CGTGTCTTTCGAGACTGGAATTCTAAATATTG
J4-II-54	CACACAAAGACAACAAGGTCATATTGTGGTTC
J4-II-55	TCCAGCAAACCCTGTTCTCAAAGCGCTTTTTT
J4-II-56	TAGGCTTGTGACGGGAGGCGATGAATGAGTAT
J4-II-57	CGTTCGTATCACTCGCCGGGCGTAAAGCCCGT
J4-II-58	CAACCTTAGGAATCGCAATGTTGATAAATTGA
J4-II-59	CCTGTTCTATGGGCAGCACTGGCCTCCAACGT
J4-II-60	TAGCTACGATTACTTTAGGGCGCTGAAATCCT
J4-II-61	AGCGACAAGGATGGAGGTTACAGCGGTTTTTT
J4-II-62	CCGCTGTAACCTCCACAAGATACCTATTTTTT
J4-II-63	AGACTACCATTGCCATCCTTGTCGCTCAGTTT
J4-II-64	CGAATAAGTCGCACCACCCTCAGGTCAGGATT
J4-II-65	TCAGCGCCCTAAAGCTATATTTGGCGACGTGC
J4-II-66	GTTTACGCTTATAGTAATCGTAGCTACACGGA
J4-II-67	AGTTCGCCCGCCCGACTGTATTGGGCACGTTG
J4-II-68	GAGGCCAGTGCTGACAAGCAACATCCTGGATG
J4-II-69	GGTTATCTAAAGTCCCATAGAACAGGCACTCC
J4-II-70	ATTGACGTAGCAAAGGAACAGCCCACTCAATT
J4-II-71	TATCAACATTGCGCAGCGACGAACCTCGTCGG
J4-II-72	CAACATCTAGTCTATTCCTAAGGTTGTGTTTC
J4-II-73	TTACCTAATGGGTGAAACTGCTTTAGACGGGC
J4-II-74	TTTACGCCCGGCGACTCCTTATCAACGATCCC
J4-II-75	TGCGCAACCATTCAGTGATACGAACGGAACCA
J4-II-76	CAATATGACCTTGAGGGTTTGCTGGAATACTC
J4-II-77	ATTCATCGCCTCCTTTCCAATCGAAATCGCCG
J4-II-78	TAGAGCATGACTACGTCACAAGCCTATTTTTT
J4-II-79	CTCTGTATAAGCGTGCAGAGACGGCTAAGGGC
J4-II-80	AAACTGTCGTAACCGGACGCAGGCTAGTTTCC
J4-II-81	GCCTCTCGGGCCAAACTGATGAGAGGGTGCTG
J4-II-82	CGTATAGTAATCCGAAGTTTAGTTACCCGAAT
J4-II-83	CGTTCATGTGAAGAATAGGAATTGTTAGTTGG
J4-II-84	TAGGTATCTTGTGGCAATGGTAGTCTCCAACT
J4-II-85	AACAATTCCTATTAAACGGTGCAAGCTTTTTT
J4-II-86	TCAGTACGATCCGCTTCACATGAACGGCACGT
J4-II-87	CGCCAAATATAGCTATAAGCGTAAACATTCGG
J4-II-88	GTAACTAAACTTCTCTCACCCTCTCATCATTC

J4-II-89	GCTCGACGGTAAAGGATTACTATACGCATCCA
J4-II-90	GGATGTTGCTTGTACTTTAGATAACCCAGCAC
J4-II-91	CCTCTCATCAGTTATTATGCTAGTGGAATAGC
J4-II-92	TACAGATGTGAGGTGGCCCGAGAGGCCCGACG
J4-II-93	AGGTTCGTCGCTGAGACTAGATGTTGGGAAAC
J4-II-94	TAGCCTGCGTCCGAGGAAAGCAGTGTTAACCA
J4-II-95	TTTGTGACATAGAGTTACGACAGTTTGGGATC
J4-II-96	GTTGATAAGGAGTGAATGGTTGCGCAGCCCTT
J4-II-97	AGCCGTCTCTGCAGTAGGAGGATTTGCTCGAA
J4-II-98	TGCGGTTAACTCTCGCTTATACAGAGCGGCGA
J4-II-99	TTTCGATTGGAAATAGTCATGCTCTATTTTTT
J4-II-100	TTTGACCCTCGGTTAAGCGGATACATGTAGCC
J4-II-101	GTGCTTCCCAAGAGATAAGGGCACCCGTCATC
J4-II-102	TATAGCGCAACCGTTCATTAATTCTGCCGTGT
J4-II-103	CCTTGCGCTAAATGGGCGTACGCGACGGTCTA
J4-II-104	GCTTGCACCGTTTCGGATCGTACTGATAGACC
J4-II-105	GTCGCGTACGCCCTCGTTTGGAGTTATTTTTT
J4-II-106	TGGAGAATGCCAAATTTAGCGCAAGGGAATGA
J4-II-107	TGAGAGGGTGAGATTTACCGTCGAGCACACGG
J4-II-108	CAGAATTAATGAACCCGATCAGCAAGGAACAA
J4-II-109	CCAACACTGACTTCGGTTGCGCTATAGCTATT
J4-II-110	CCACTAGCATAATCCTCACATCTGTAGATGAC
J4-II-111	GGGTGCCCTTATCATCTCCAAGTCGGTCGGCC
J4-II-112	GCTTTGGTCTGCCTCTTGGGAAGCACTGGTTA
J4-II-113	ACACTGCTTTCCTTCTATGTCACAAAGGCTAC
J4-II-114	ATGTATCCGCTTATCGCTCACACGGATGCCTA
J4-II-115	ACTACGGTAATCAACCGAGGGTCAAATTCGAG
J4-II-116	CAAATCCTCCTACAGAGTTAACCGCATTTTTT
J4-II-117	GCATGAAGTCCGATTAATGGCACGTGTAGCAA
J4-II-118	AAAGGAAGGTCGTGCCGACCCGTATCTTCTAC
J4-II-119	ACATGTTGCGAGTGGGTTCGCTCCAGCCACTC
J4-II-120	TAACTCCAAACGATTGGCATTCTCCAGAGTGG
J4-II-121	CTGGAGCGAACCCTTGCCCGGGCGCATTTTTT
J4-II-122	TTAAGACCTCGAAACTCGCAACATGTTTGTTC
J4-II-123	CTTGCTGATCGGGAAGTCAGTGTTGGGTAGAA
J4-II-124	GATACGGGTCGGCCAGTGCTAGTGTCCATCGG
J4-II-125	TGTTTACGGATTCACGACCTTCCTTTGGCCGA
J4-II-126	CCGACTTGGAGATGGCAGACCAAAGCTTGCTA
J4-II-127	CACGTGCCATTAAAGCATCAGATTTAAGATAC
J4-II-128	CAAGAAGGTTCATTCGGACTTCATGCTAGGCA
J4-II-129	TCCGTGTGAGCGATGATTACCGTAGTTTTTTT
J4-II-130	TCTTTAAAGTTAGAGGGCTGTATGAGCGACTC
J4-II-131	GCCGGCGACCGTAACTTTAAAGTACCACCGCC
J4-II-132	TGCGCCCGGGCAATTCGAGGTCTTAAGGCGGT
J4-II-133	GGTACTTTAAAGTAGCTGTGGAAGAATTTTTT

J4-II-134	AAGTACATATCAATACGGTCGCCGGCCCGATG
J4-II-135	GACACTAGCACTGGAATCCGTAAACAGAGTCG
J4-II-136	CTCATACAGCCCTCCCGCCTCGTTTACCTCAA
J4-II-137	AGGCTTGTCCCGACTAACTTTAAAGAGTATCT
J4-II-138	TAAATCTGATGCTATGAACCTTCTTGTTTTTT
J4-II-139	CTTGCGAATTACAACTCATTTAACTTGTGTTC
J4-II-140	TTCTTCCACAGCTTTGATATGTACTTGAACAC
J4-II-141	AAGTTAAATGAGTCTGTATAAGACGATTTTTT
J4-II-142	TTATATTGTGATCTGTAATTCGCAAGTTGAGG
J4-II-143	TAAACGAGGCGGGTCGGGACAAGCCTTTTTTT
J4-II-144	TCGTCTTATACAGGATCACAATATAATTTTTT

J4-III(16bp10bp)	
NAME	SEQUENCE
J4-III-1	GATTTGCGAGAATGGCGACTTGGGAAGGACATTTTTTTTT
J4-III-2	TGTCCCTTCCCAAGTCTCACACCGCAATCCACTTGGCCAGGA
J4-III-3	AGTGTTATACACGCTCGCCATTCTCGCAAATCTTTTTTTTT
J4-III-4	TAGGGCCCTTGCCGGAGTCAGTCGCCAGAAAGGGAATGCAGT
J4-III-5	TAGCCCTCCACCGTTAAAGATTTAAGCGATTGTTTTTTTTT
J4-III-6	CAATCGCTTAAATCTTTAGCGGGAGTCGGGAGAAATACAAAG
J4-III-7	ATAATCCCGGGAAAGCTAACGGTGGAGGGCTATCCTGGCCAA
J4-III-8	GTGGATTGCGGTGTGAGAGCGTGTATAACACTACTGCATTCC
J4-III-9	CTTTCTGGCGACTGACGTATCCAATGGAATCAGTCACCGACC
J4-III-10	GAGGTAAACCCGCGTGTCCGGCAAGGGCCCTATTTTTTTTT
J4-III-11	AGCGCAAAGCGTCGTAGGCACGGTGAGGGACGTGCGGCGCGG
J4-III-12	AGCACTTTAGTTCATCTATATTGCCTTAAGCTGTGTGTGCTA
J4-III-13	GCACGTCAGATATGCTCTTAGAAAGTATGAGGTTTTTTTTT
J4-III-14	CCTCATACTTTCTAAGGAAGATAGATATACGCTGCTGCGGGC
J4-III-15	CTTGCAAGAGCGTACGAGCATATCTGACGTGCCTTTGTATTT
J4-III-16	CTCCCGACTCCCGCTAGCTTTCCCGGGATTATTAGCACACAC
J4-III-17	AGCTTAAGGCAATATATTAATGCTCTGGGCTAGCTATCCACG
J4-III-18	CCTTCCAGAATACGGCGATGAACTAAAGTGCTGGTCGGTGAC
J4-III-19	TGATTCCATTGGATACCACGCGGGTTTACCTCCCGCGCCGCA
J4-III-20	CGTCCCTCACCGTGCCGGGAGTTGAATGGCCTGGAACTGAGC
J4-III-21	TAGCGAGTCCGTCCCTTACGACGCTTTGCGCTTTTTTTTTT
J4-III-22	GAAGGGAGAACATACCATTACCAGAGCATTTACGCGTTACTT
J4-III-23	CCAATATGGTACCCTCGATAAGGGCTGTATTATTCACGAGTG
J4-III-24	TCAAGTCAGCCCTAACTTTGTTACGGTTACGTCGTCCAGCAT
J4-III-25	AAATACGTTTAGTCTTTAGTATCCGTGCGTCGTTTTTTTTT
J4-III-26	CGACGCACGGATACTAATCGCTCGTCGGTGAGGGTAGAGGTC
J4-III-27	TCTCGTTACGCGACACAAGACTAAACGTATTTGCCCGCAGCA
J4-III-28	GCGTATATCTATCTTCCGTACGCTCTTGCAAGATGCTGGACG
J4-III-29	ACGTAACCGTAACAAAGAGGGCTAAGATGCTTAGGTGGTCGG
J4-III-30	ATGGTGAATACGTGAGGTTAGGGCTGACTTGACGTGGATAGC
J4-III-31	TAGCCCAGAGCATTAAGCCGTATTCTGGAAGGCACTCGTGAA
J4-III-32	TAATACAGCCCTTATCCTAGATCGCGACGCTGCAGGATTATT
J4-III-33	GTCATGTGACTACGGAGAGGGTACCATATTGGGCTCAGTTCC
J4-III-34	AGGCCATTCAACTCCCAGGGACGGACTCGCTAAAGTAACGCG
J4-III-35	TAAATGCTCTGGTAATGGCGAGAAAGCGGAGGGCATAGCGAA
J4-III-36	ACGGTGATATTCAAAGGGTATGTTCTCCCTTCTTTTTTTTT
J4-III-37	ATACATGCCACGGCACGCCGTAGTACCGGGAAGTTCTTTGTA
J4-III-38	GAACCAGTGCATCGACTCAGATAACTCGATAATGCTACAGAT
J4-III-39	ATCCGTTAGTTCGCGCTGCACGTATAACAGTTATACGGTGTA
J4-III-40	GCTGACAGGGCTATTTAGTCCAGTGACCGCTGGGCTGTTTAG
J4-III-41	CGATCTAACAGCCGCTGCCCATAGCATCACAATTTTTTTTT
J4-III-42	TTGTGATGCTATGGGCGAGGCACACCACTGATGCGAGTGGTT
J4-III-43	CTAAGTCCGCACCAGGAGCGGCTGTTAGATCGGACCTCTACC

J4-III-44	CTCACCGACGAGCGATGTGTCGCGTAACGAGACTAAACAGCC
J4-III-45	CAGCGGTCACTGGACTGGCGTACCCTACGCCTGATTGCCGTA
J4-III-46	GCGGGCGAGACCCTTGAAATAGCCCTGTCAGCCCGACCACCT
J4-III-47	AAGCATCTTAGCCCTCCTCACGTATTCACCATTACACCGTAT
J4-III-48	AACTGTTATACGTGCACCCGCAGTACGCGTTGACTGCTATGC
J4-III-49	TGCTCGCTGAACCTGAGCGCGAACTAACGGATAATAATCCTG
J4-III-50	CAGCGTCGCGATCTAGTCCGTAGTCACATGACATCTGTAGCA
J4-III-51	TTATCGAGTTATCTGAACTCTCAGTTGGTAAGTTTGTTTCAG
J4-III-52	ACGCCTCTGGGTGTAAGTCGATGCACTGGTTCTTCGCTATGC
J4-III-53	CCTCCGCTTTCTCGCCCTTTGAATATCACCGTTACAAAGAAC
J4-III-54	TTCCCGGTACTACGGCATCTAACCCTGTGTCCGTGAACTGTT
J4-III-55	TGATGCCATTTAGTTGGTGCCGTGGCATGTATTTTTTTTTTT
J4-III-56	GCTCGTAGAACATACAGATCCACGGTCAGAAATTCGCCACTC
J4-III-57	TGACTCGCCACTCGTAAATTCAGGGATCCTCGGCAAGCCAAC
J4-III-58	AAAGCAGATAGGAGTTCGGGCCGCCTTGGCGAGCGCACTCTG
J4-III-59	CGCTAAAGGTCAAAGCAACCAGTTCGCCATTAATAGAATGAC
J4-III-60	TTGCTCCAATTGGGATACCCTTTACTTGCAATTACCACTATT
J4-III-61	TATATATTCTGAAAGAAGGTTCATGGTACCATTTTTTTTTTT
J4-III-62	ATGGTACCATGAACCTCCGAGAACGGCCTGCCTTTTTTTTTT
J4-III-63	TAGAACAGGTAGAGTATCTTTCAGAATATATAAACCACTCGC
J4-III-64	ATCAGTGGTGTGCCTCCCTGGTGCGGACTTAGAATAGTGGTA
J4-III-65	ATTGCAAGTAAAGGGTCGGTACCGGAATGCGGTCGCATAATC
J4-III-66	GTGGTTGATCTCATAAATCCCAATTGGAGCAATACGGCAATC
J4-III-67	AGGCGTAGGGTACGCCCAAGGGTCTCGCCCGCGTCATTCTAT
J4-III-68	TAATGGCGAACTGGTTTCAACAGAAATGGAACAGGCAGAGAC
J4-III-69	GTGCGGTGGCATCTGAGCTTTGACCTTTAGCGGCATAGCAGT
J4-III-70	CAACGCGTACTGCGGGTCAGGTTCAGCGAGCACAGAGTGCGC
J4-III-71	TCGCCAAGGCGGCCCGATCTCCGTGATACTCTCGTGGGCATT
J4-III-72	ACCACTATGCCTTCACAACTCCTATCTGCTTTCTGAAACAAA
J4-III-73	CTTACCAACTGAGAGTTTACACCCAGAGGCGTGTTGGCTTGC
J4-III-74	CGAGGATCCCTGAATTGACGTGAATGCAATTTGCCTGTGCTC
J4-III-75	TCACCTTCGCACGCACTACGAGTGGCGAGTCAAACAGTTCAC
J4-III-76	GGACACAGGGTTAGATCAACTAAATGGCATCAGAGTGGCGAA
J4-III-77	TTTCTGACCGTGGATCCGGCCTGAGAGTGCCGCCCTTATTGA
J4-III-78	GTTGTCATACGATACGTGTATGTTCTACGAGCTTTTTTTTTT
J4-III-79	GCCCTTTGATCCGTTGCGAAGGTCTCCCGCCAGAGCTTAGGC
J4-III-80	TCGGATTATCTACGTACTCCGAGGGAGGGTCTCTACGCTTGA
J4-III-81	CTGTCATTGGTTTGATTGATCGCCCTTACGGAGAGTGTGCTG
J4-III-82	ATCGAGAAGGTGTTCGAGGTGAGCCCGCTACCTAAGGGACTT
J4-III-83	AAACGCCTTACCACTGAGATCAATATCTAAGCCTTATCATCA
J4-III-84	GGCAGGCCGTTCTCGGTACTCTACCTGTTCTATGATGATAAG
J4-III-85	GCTTAGATATTGATCTTGCTCGGTTGAGGTACTTTTTTTTTT
J4-III-86	CGACCATTAAGGATCCCAGTGGTAAGGCGTTTGATTATGCGA
J4-III-87	CCGCATTCCGGTACCGTTATGAGATCAACCACAAGTCCCTTA
J4-III-88	GGTAGCGGGCTCACCTTCGAGCTTTAGTAAAGTCAAGGGCGG

J4-III-89	TCCCAGGCTTAATGAACGAACACCTTCTCGATGTCTCTGCCT
J4-III-90	GTTCCATTTCTGTTGATCAGATGCCACCGCACCAGCACACTC
J4-III-91	TCCGTAAGGGCGATCATCAATAGAAAGACATCCCATCGTCGC
J4-III-92	GCCCTAAGCCACGCCTATCAAACCAATGACAGAATGCCCACG
J4-III-93	AGAGTATCACGGAGATGTGAAGGCATAGTGGTTCAAGCGTAG
J4-III-94	AGACCCTCCCTCGGAGCGGCCTCCTGGTGAGTAGGCTCCGTA
J4-III-95	ACCCTCTCGGTTCGACTACGTAGATAATCCGAGAGCACAGGC
J4-III-96	AAATTGCATTCACGTCGTGCGTGCGAAGGTGAGCCTAAGCTC
J4-III-97	TGGCGGGAGACCTTCGTGTAGCCTTATTGTAGTAAATTGTGA
J4-III-98	TGCACGGTACCTTAGACAACGGATCAAAGGGCTCAATAAGGG
J4-III-99	CGGCACTCTCAGGCCGCGTATCGTATGACAACTTTTTTTTTT
J4-III-100	GCCCTTCGATCGTACTTGAAGTGCAACTAGGGTATAATGCCG
J4-III-101	TCTTCCAGAAGCAGCGTTAGTCGCTGTGAAGCAGAAGGCTTT
J4-III-102	CACTCGCGAAAGTATTCGCCTAGGGCACTCCGTAACTGTTGT
J4-III-103	TCGTGGAAGCCTACTATACTGTTCACTACATTGTTCTTTAAA
J4-III-104	GTACCTCAACCGAGCAGGATCCTTAATGGTCGTTTAAAGAAC
J4-III-105	AATGTAGTGAACAGTAAGCACACCCTATTAACTTTTTTTTTT
J4-III-106	ATTCAAGTCTTTGAGGTAGTAGGCTTCCACGACCGCCCTTGA
J4-III-107	CTTTACTAAAGCTCGATTCATTAAGCCTGGGAACAACAGTTA
J4-III-108	CGGAGTGCCCTAGGCGCTCTGCGTATATCCCTCTTGAAGGGC
J4-III-109	GTGTGTGTCTTGAGTGAATACTTTCGCGAGTGGCGACGATGG
J4-III-110	GATGTCTTTCTATTGAAGGCGTGGCTTAGGGCAAAGCCTTCT
J4-III-111	GCTTCACAGCGACTAAAGCCTGGAGCCCTAGTCACAATCCGC
J4-III-112	GTAATTTAAGTCGAAGCGCTGCTTCTGGAAGATACGGAGCCT
J4-III-113	ACTCACCAGGAGGCCGGTCGAACCGAGAGGGTCGGCATTATA
J4-III-114	CCCTAGTTGCACTTCACACTAGGTAGTCAAACGTTCCGGCGC
J4-III-115	AATCAGTCGCGGTTGAAGTACGATCGAAGGGCTCACAATTTA
J4-III-116	CTACAATAAGGCTACATCTAAGGTACCGTGCATTTTTTTTTT
J4-III-117	GTGAACCCTTTCCACATCATGCACACAACCCGCCCAATGAGT
J4-III-118	AAGTTGTACAAACATGTGCTTGTTCCGGCTTGGTCACGAGCC
J4-III-119	AGTAAAGGGCTTGAGTCGTAAGAAGAATGTTTCCCTAGGATT
J4-III-120	GTTAATAGGGTGTGCTCCTCAAAGACTTGAATAATCCTAGGG
J4-III-121	AAACATTCTTCTTACGTTAATGCCATTCGCGGTTTTTTTTTT
J4-III-122	ACACCACTCTGACAAGACTCAAGCCCTTTACTGCCCTTCAAG
J4-III-123	AGGGATATACGCAGAGCACTCAAGACACACACGGCTCGTGAC
J4-III-124	CAAGCCGGAACAAGCAAGACGTGTCCGCGTCTACGTGAGTGG
J4-III-125	GTGATTTGTGACATTTCATGTTTGTACAACTTGCGGATTGTG
J4-III-126	ACTAGGGCTCCAGGCTCTTCGACTTAAATTACACTCATTGGG
J4-III-127	CGGGTTGTGTGCATGAGAATAACGTCGCGGAGAGTGCTTCGA
J4-III-128	GAGTGGTGCCCTGAATTGTGGAAAGGGTTCACGCGCCGGAAC
J4-III-129	GTTTGACTACCTAGTGTCAACCGCGACTGATTTTTTTTTTTT
J4-III-130	AGGGTATTCTGTCCAGAGCGTGAAAGACCAGCTTGAGAATTG
J4-III-131	TAAGGGACTTCCATCTTACTATAGTACAAAGTTCTCAGGTGT
J4-III-132	CCGCGAATGGCATTAACTTGTCAGAGTGGTGTACACCTGAGA
J4-III-133	ACTTTGTACTATAGTAGGTCAAGTATTGTGTATTTTTTTTTT

J4-III-134	TCAGGGCCTCACAAATAGATGGAAGTCCCTTACCACTCACGT
J4-III-135	AGACGCGGACACGTCTAAATGTCACAAATCACCAATTCTCAA
J4-III-136	GCTGGTCTTTCACGCTATATTATTCTAAGGGCTATCGGTCGG
J4-III-137	CCTGTAGTCCCGGAAGCTGGACAGAATACCCTTCGAAGCACT
J4-III-138	CTCCGCGACGTTATTCATTCAGGGCACCACTCTTTTTTTTTT
J4-III-139	TATGATCTGCAGGATGCTCTGCGAACACATTCCCACATAGGG
J4-III-140	TACACAATACTTGACCATTTGTGAGGCCCTGACCCTATGTGG
J4-III-141	GAATGTGTTCGCAGAGCCTTCCGCCGAAGCTATTTTTTTTTT
J4-III-142	ACCTCTTCTCAACGTGCATCCTGCAGATCATACCGACCGATA
J4-III-143	GCCCTTAGAATAATATCTTCCGGGACTACAGGTTTTTTTTTT
J4-III-144	TAGCTTCGGCGGAAGGCACGTTGAGAAGAGGTTTTTTTTTTT

J3(11bp10bp)	
NAME	SEQUENCE
J3-1	TTTTTTTTTTCTTATCGGGCCTACGGCTTGT
J3-2	AGCGTCTGGCGTCGTAGAGGCGCCCGATAAGA
J3-3	CCAGAAAGGTACAAGCCGTAGGCCTCTACGAC
J3-4	ACCTTTCTGGCCTTGGCTATTAGCGGCGCTCA
J3-5	AGTCCACGATTGAACGACAAGAATAGCCAAGG
J3-6	TTTTTTTTTTGAGCGCCGCTCTTGTCGTTCA
J3-7	ATCGTGGACTCATTTAAGTAAAGCTCGGGTAT
J3-8	TCCGTGGATCATTGTTAGACGTTACTTAAATG
J3-9	CATGCGCCGCATACCCGAGCTCGTCTAACAAT
J3-10	GCGGCGCATGACTGACTGAAGGGCAATATTTA
J3-11	ATACAAGAATTACAGATTGATCTTCAGTCAGT
J3-12	TTTTTTTTTTAAATATTGCCATCAATCTGTA
J3-13	ATTCTTGTATTCACAGCGCCAGGCTTGCACGA
J3-14	AATCGTAGGCACGCAATAATATGGCGCTGTGA
J3-15	TTAGCTGGTATCGTGCAAGCCTATTATTGCGT
J3-16	TACCAGCTAAATCAGACACCAATTTCAGGTCT
J3-17	TTGAATTCCGATTCGACGATTTGGTGTCTGAT
J3-18	TTTTTTTTTAGACCTGAAATAATCGTCGAAT
J3-19	CGGAATTCAACCTCAGTATATGTGACGCACCT
J3-20	CCGAAAGAATTCCACGTGGCGATATACTGAGG
J3-21	TTTTTTTTTAGGTGCGTCACCGCCACGTGGA
J3-22	ATTCTTTCGGTGGTTAAACGACTGCAGCAATC
J3-23	CCTGCCAGACAAGCACCTCATTCGTTTAACCA
J3-24	TTTTTTTTTGATTGCTGCAGATGAGGTGCTT
J3-25	GTCTGGCAGGCATCCAAATCGGCTCGGACCTA
J3-26	GATCAGCTTGATGTCCTGAGTCGATTTGGATG
J3-27	GCCCTAGACATAGGTCCGAGCACTCAGGACAT
J3-28	CAAGCTGATCACCTCGAAGCAAATCAGTTGTC
J3-29	GCCTACGATTAGACAAGCGGATGCTTCGAGGT
J3-30	AGCCCTCTTTGACAACTGATTTCCGCTTGTCT
J3-31	AAAGAGGGCTTTAAATGAGTCGAAGTTTAAAG
J3-32	ACAAATGAAGTAGATTGCCTCGACTCATTTAA
J3-33	CAAATGAACGCTTTAAACTTCGAGGCAATCTA
J3-34	CTTCATTTGTAAAGAGTGGGACACGGTCATAA
J3-35	GATCCACGGAAACTGCCCGAATCCCACTCTTT
J3-36	CTGCGTGCCGTTATGACCGTGTTCGGGCAGTT
J3-37	CGGCACGCAGCATCTGGAATAGACACTATCCT
J3-38	AACGTACGATGCTTCCTCTCTTATTCCAGATG
J3-39	AATGACCTCGAGGATAGTGTCAGAGAGGAAGC
J3-40	ATCGTACGTTCGATATTGCAGGTACTCTGCTT
J3-41	GCCAGACGCTGGTCCTACTGCCTGCAATATCG
J3-42	TTTTTTTTTAAGCAGAGTACGCAGTAGGACC
J3-43	TTTTTTTTTCCATGCCAGCATTTGTGAGACC

J3-44	CCGATTGTCACGTGTACCCGCTGCTGGCATGG
J3-45	CTAGTTCATAGGTCTCACAAAGCGGGTACACG
J3-46	TATGAACTAGGACCCGTTTATGTAACCGCGGG
J3-47	TATAGTTCGTACCATCGCGAGATAAACGGGTC
J3-48	CGAGGTCATTCCCGCGGTTACCTCGCGATGGT
J3-49	ACGAACTATAGAAATCCGCTTTGAGGCGAAAG
J3-50	GCTGTAAGGCCAGTTCGTTTAAAGCGGATTTC
J3-51	TCATTGGACCCTTTCGCCTCATAAACGAACTG
J3-52	GGTCCAATGAAGTAACGAGACAGAAGTAGATA
J3-53	TACTTAGAGTTCCCTTGGTGGGTCTCGTTACT
J3-54	CGTTCATTTGTATCTACTTCTCCACCAAGGGA
J3-55	ACTCTAAGTATAATGTGGAAATACGCCTGGTT
J3-56	GCGTGATGAGGTGACTTTACGTTTCCACATTA
J3-57	CGGCCTCAATAACCAGGCGTACGTAAAGTCAC
J3-58	ATTGAGGCCGAGAAAGCGCGATTAGCAGACAT
J3-59	CGGCGATTCAGACAGCCAGCTTCGCGCTTTCT
J3-60	TGTCTAGGGCATGTCTGCTAAAGCTGGCTGTC
J3-61	TGAATCGCCGTCGGAAGATACAGCTCAATTAC
J3-62	GTGCTTTAATCCCACTACCTTGTATCTTCCGA
J3-63	TTTTTTTTTTGTAATTGAGCTAAGGTAGTGGG
J3-64	ATTAAAGCACGAGGTATTCGGAAGGGACCATG
J3-65	TATGGGCAGTGGATTGCTGGGCCGAATACCTC
J3-66	TTTTTTTTTTCATGGTCCCTTCCCAGCAATCC
J3-67	ACTGCCCATAACGAACCTCCTTGTCGGGACAC
J3-68	GGCCAAGCTACCGTCACTTATAGGAGGTTCGT
J3-69	ACTGTGCATGGTGTCCCGACAATAAGTGACGG
J3-70	TAGCTTGGCCTTCAATTATCTAGAATACTGAG
J3-71	CTCATCACGCCATTTGCGTGGAGATAATTGAA
J3-72	AAATGCTTACCTCAGTATTCTCCACGCAAATG
J3-73	GTAAGCATTTCTCATGAAAGGAAAGGGCCGTC
J3-74	CCGTATAAAGGTTATCGTTATCCTTTCATGAG
J3-75	CGCAACTCCGGACGGCCCTTTATAACGATAAC
J3-76	CTTTATACGGTTCCTGCAAGAGTTCTACGGTC
J3-77	GCCTTACAGCATACGTGTGGCTCTTGCAGGAA
J3-78	CATTTAGAATGACCGTAGAACGCCACACGTAT
J3-79	ATTCTAAATGCCTCGGGTAGCAATACCCAGGG
J3-80	TAACAGGTTGTTGGGAAAGACGCTACCCGAGG
J3-81	CTATGGCGATCCCTGGGTATTGTCTTTCCCAA
J3-82	CAACCTGTTACAACCACGTGTCACTGCCTATT
J3-83	TGACAATCGGTGTCCTGCCCTACACGTGGTTG
J3-84	TTTTTTTTTTAATAGGCAGTGAGGGCAGGACA
J3-85	TTTTTTTTTTCATGTTAAGCCCGATTTGGCCC
J3-86	CTTTGTCCGGCTCACTGCCCTGGCTTAACATG
J3-87	GTCGAAAGTTGGGCCAAATCGAGGGCAGTGAG
J3-88	AACTTTCGACCTGGTGATCCTCGCGGATCAGC

J3-89	TATGCTGTAGTCCCTTGTCTGAGGATCACCAG
J3-90	ATCGCCATAGGCTGATCCGCGCAGACAAGGGA
J3-91	CTACAGCATAACATGGGAGATTACAACCTAAA
J3-92	GTCACCGGTCTGTTCAGTATAATCTCCCATGT
J3-93	AGGTTAAAGATTTAGGTTGTATATACTGAACA
J3-94	TCTTTAACCTCCGTGTCTGTGTATTTAAAGTT
J3-95	TTAGTAAGGTAGACTGTATTCCACAGACACGG
J3-96	CGGAGTTGCGAACTTTAAATAGAATACAGTCT
J3-97	ACCTTACTAAAGAGCAAAGGATGTCGGAGGCT
J3-98	ATCAAGCATCCACCCTTACCTTCCTTTGCTCT
J3-99	TGACATGAAGAGCCTCCGACAAGGTAAGGGTG
J3-100	CTTCATGTCATCGGGCTGAAGTTTAACCCTAC
J3-101	TTCTCCCTCTCCATGCAGGTGCTTCAGCCCGA
J3-102	CATGCACAGTGTAGGGTTAAACACCTGCATGG
J3-103	AGAGGGAGAAATGGAGGGATAACCGATAAGGT
J3-104	AACAATTATGAGAATCACCATTATCCCTCCAT
J3-105	TTTTTTTTTTACCTTATCGGTATGGTGATTCT
J3-106	CATAATTGTTTACGGTCGGTCGTATTACACCT
J3-107	ACAGCTACTGCACGCTCGTCGGACCGACCGTA
J3-108	TTTTTTTTTTAGGTGTAATACCGACGAGCGTG
J3-109	CAGTAGCTGTCCTGGCGCGTGCGATAGTGCAT
J3-110	AAACATACTAAGAGTTCCATCCACGCGCCAGG
J3-111	TTTTTTTTTTATGCACTATCGGATGGAACTCT
J3-112	TAGTATGTTTGCTGGCTGATCTTTGGAAGGGC
J3-113	GATGCTTGATAAGACTTAGGAGATCAGCCAGC
J3-114	TTCATCCGTGGCCCTTCCAAATCCTAAGTCTT
J3-115	CACGGATGAACTCTTGAACGGTCAGACACTCG
J3-116	TGTTGTGAATACCGGTTTACTCCGTTCAAGAG
J3-117	TTTTTTTTTTCGAGTGTCTGAAGTAAACCGGT
J3-118	ATTCACAACAGCGCGTAGATCGATCTCACTAT
J3-119	GACCGGTGACGGGTCAGCAAAGATCTACGCGC
J3-120	CAAGCGACAGATAGTGAGATCTTTGCTGACCC
J3-121	CTGTCGCTTGAGTGGGCTAGCTTCAGAGCTGT
J3-122	TGCGTAGAGGGAATCTGCGGCGCTAGCCCACT
J3-123	TTTTTTTTTTACAGCTCTGAAGCCGCAGATTC
J3-124	CCTCTACGCACACCAGGGCCCGCAGCGACATT
J3-125	CCGGACAAAGGCTCTCCCTACGGGCCCTGGTG
J3-126	TTTTTTTTTTAATGTCGCTGCGTAGGGAGAGC

DX-I(21bp11bp10bp)	
NAME	SEQUENCE
DX-I-1	CTGCTACCATACTGGTACTTACAGGATCATGTTATTTGGCGAC
DX-I-2	GTGCCCTAAAGTGAATAAATT
DX-I-3	GAGACGTCGCCCAGACCTCAA
DX-I-4	CTTGCCTTTACAAGTAAGGGT
DX-I-5	GTCTATTACCAGCCGGCGTGTGTTACCACGGGTGTATCCGCAA
DX-I-6	CTCGGCCGGGAAGACCCATTT
DX-I-7	AGAGGCAGGCTAAATTAGACT
DX-I-8	CTAAAGGGAAGTTGCCCAGAC
DX-I-9	CGGCTTATAGGGCATAGCCATCAACAGATGCGCGTTCATTAGG
DX-I-10	AAGTTCATATAGTGGTAATAA
DX-I-11	TAATATAGTAGGCTAAGCAAC
DX-I-12	TCGTCCTTATCTTTTTTTTTT
DX-I-13	GATAAGGACGAGTTGCTTAGCCTACTATATTATTATTACCACT
DX-I-14	ATATGAACTTCCTAATGAACGGTCTTACACCGCTTACACGAT
DX-I-15	GTCGGACTGGCGCATCTGTTGATGGCTATGCCGAAGTTTAGG
DX-I-16	AAGCTGTATGTAGATGTCCTACCTATAAGCCGGTCTGGGCAA
DX-I-17	CTTCCCTTTAGAGTCTAATTTAGCCTGCCTCTAAATGGGTCTT
DX-I-18	CCCGGCCGAGTTGCGGATACAGCAATGATGATGGAATGTGTC
DX-I-19	TCAGAAGCCACCCGTGGTAACACACGCCGGCGTGAGCTTCAC
DX-I-20	TCGATGGTCTACTCATGGCGCTGGTAATAGACACCCTTACTT
DX-I-21	GTAAAGGCAAGTTGAGGTCTGGGCGACGTCTCAATTTATTCAC
DX-I-22	TTTAGGGCACGTCGCCAAATAACACGAGTTATATCCGGGACG
DX-I-23	TCAATTAGGTACATGATCCTGTAAGTACCAGACAAATGGGCA
DX-I-24	TTTTTTTTTTACGCTATAACCTATGGTAGCAGTTTTTTTTTT
DX-I-25	ACTCGCGAGCAGCCTCTATGGTGGGCTGATTCCCTCACGGGCC
DX-I-26	GTGAGCTTTAAGATGGTGCCCTCGGTGCGACACGGGCAATATC
DX-I-27	GTCCAAATATCGACTATCCTCACGAGCTGCACCAGCTATGGTC
DX-I-28	TTTTTTTTTTGACCATAGCTGCTGAGGGACTTTTTTTTTTT
DX-I-29	AAGCAAGGTAGTGCAGCTCGTGAGGATAGTCGCACTGTCGAC
DX-I-30	CGAGAAGATATCTCTATATAAGATATTTGGACATCGTGTAAG
DX-I-31	CGGTGTAAGACCCAGTCCGACCCTAAACTTCGTAGGACATCTA
DX-I-32	CATACAGCTTGATATTGCCCGTCCAACTGGGTATATAATGGG
DX-I-33	GACCGACGCATGTCGCACCGAGGGCACCATCGAGAGGTGGTG
DX-I-34	TTCCCTAGTGATTTCACTCACTTAAAGCTCACGACACATTCC
DX-I-35	ATCATCATTGCTGGCTTCTGAGTGAAGCTCACGCGCCATGAGT
DX-I-36	AGACCATCGAGGCCCGTGAGGGCACTGCCTCGCCTAAAGCCT
DX-I-37	CGAGCTACGTGAATCAGCCCACCATAGAGGCAGCCAGTTGCT
DX-I-38	AGTTTACACTGATTTAGGTGCTGCTCGCGAGTCGTCCCGGAT
DX-I-39	ATAACTCGTGTACCTAATTGATGCCCATTTGTGGTTATAGCGT
DX-I-40	CAATCGGCCTACATACAACGCCTTCGCCTTCTAGCCGATAGTC
DX-I-41	TACTACCCATTACCCTGATGTTCCCGACCAAACACGATTGTTG
DX-I-42	CCCGACACGAGCGACAAACCCTAAGTACGCAACCATGAGCCAT
DX-I-43	AAGTCCCTCAGTACCTTGCTTGTCGACAGTGCTTATATAGAGA

DX-I-44	TATCTTCTCGATGGCTCATGGTCTCTTGAAATAGTTCGGTAC
DX-I-45	AGTTTGCAATTTGCGTACTTAGGGTTTGTCGTCAGTGTGAAG
DX-I-46	TCTCCCTACCCTTAACTCGTGCTCGTGTCGGGCCCATTATAT
DX-I-47	ACCCAGTTGGATGCGTCGGTCCACCACCTCTCGTGAGTGAAAT
DX-I-48	CACTAGGGAACAACAATCGTGAAACGTGATTTAGTGGTTAGC
DX-I-49	AGGGCGGATGTTTGGTCGGGAACATCAGGGTCTGTCGAAGCT
DX-I-50	TGATTGGAGACGGCATGCGTCAATGGGTAGTAAGGCTTTAGG
DX-I-51	CGAGGCAGTGCACGTAGCTCGAGCAACTGGCTGCACCTAAATC
DX-I-52	AGTGTAAACTGACTATCGGCTATGTAATGGTAGTCCGACGTT
DX-I-53	TCGTCTCTACAGAAGGCGAAGGCGTTGTATGGAGTGGAGGAA
DX-I-54	TTTTTTTTTTGTGTCCGGACCTAGGCCGATTGTTTTTTTTTT
DX-I-55	ACATGCTAAGCATATAGCTAAGCGATATATTACAGGACACACG
DX-I-56	CAGCCCTCAGTCTCGCGCTTCGCCGCGAGGGCGAACTTTAACA
DX-I-57	ATGTCGGCGTATCTTAAGAACACATTTATCGTACCACATTGGG
DX-I-58	TTTTTTTTTTCCCAATGTGGTTGATCTGTGTATTTTTTTTTT
DX-I-59	TAGAAGAAATACGATAAATGTGTTCTTAAGATGTTATGGTCT
DX-I-60	GAGGCTCTCTCCAGTCACAGGTACGCCGACATGTACCGAACT
DX-I-61	ATTTCAAGAGAATTGCAAACTCTTCACACTGACACGAGTTAAG
DX-I-62	GGTAGGGAGATGTTAAAGTTCCCGATCGCGTAGCAGGAAAGA
DX-I-63	AACGGCGCTTGCCCTCGCGGCGAAGCGCGAGCGGTCGGCGCT
DX-I-64	CAGGCCCTGCAAGACTATCTCACTGAGGGCTGGCTAACCACT
DX-I-65	AAATCACGTTTCATCCGCCCTAGCTTCGACAGGACGCATGCCG
DX-I-66	TCTCCAATCACGTGTGTCCTGAATGCGGTCTTCCTGTTCTAA
DX-I-67	GCGCGTCAGTTAATATATCGCTTAGCTATATTCGTAATATGA
DX-I-68	ATTCGCCAGAGATACATTTCGGCTTAGCATGTAACGTCGGAC
DX-I-69	TACCATTACATGTAGAGACGATTCCTCCACTCGGTCCGGACAC
DX-I-70	TTCCGTGAGCGATAGGCTAAGAACGGTTTACGGCTTACTTCGA
DX-I-71	AGGTGGTCCGTGGCGAGGATCCACAGGGAAATTTACACTGGAC
DX-I-72	GCGTCACCACCGCTACCTCAACGTGATCGTAGAGCGATGGAAT
DX-I-73	TACACAGATCAATTTCTTCTAAGACCATAACACCTGTGACTGG
DX-I-74	AGAGAGCCTCATTCCATCGCTGACTCCGCAAGGCGTGCGTGT
DX-I-75	AAGCGGTACCCTACGATCACGTTGAGGTAGCACGATGGCATT
DX-I-76	TACGGATAAGGCATCCCGTCTGGTGGTGACGCTCTTTCCTGC
DX-I-77	TACGCGATCGGAAGCGCCGTTAGCGCCGACCGGAGATAGTCTT
DX-I-78	GCAGGGCCTGGTCCAGTGTAAGTGGCGTTCAGCTTCGCGTTA
DX-I-79	GGGCAATCCCATTTCCCTGTGGATCCTCGCCCGGTTTACTGC
DX-I-80	GTCTACAGTTAACCAGAGCTAACGGACCACCTTTAGAACAGG
DX-I-81	AAGACCGCATTACTGACGCGCTCATATTACGACGAAATGTATC
DX-I-82	TCTGGCGAATTCGAAGTAAGCACGTGGTCTATTTACGAGTGC
DX-I-83	GGGCTAGGACCGTAAACCGTTCTTAGCCTATGAACTCAGTCC
DX-I-84	TTTTTTTTTTCTCAAACGCTCCGCTCACGGAATTTTTTTTTT
DX-I-85	TAGCGGCGTTGTACTCCTGACTAAATGGGTGGAGGTGGGCTAC
DX-I-86	AAGCGGCGTTAAATAAAGGCATTTCTTGCGCGCGGGTTCTAGG
DX-I-87	GCCCTGAACTCTGTACACTAAGTATCTATTGCTCCCTTTACAC
DX-I-88	TTTTTTTTTTGTGTAAAGGGAGAGGACTACCGTTTTTTTTTT

DX-I-89	CTTCCTTTCTGCAATAGATACTTAGTGTACACGCTGAGTCCG
DX-I-90	AAACGGCGCGCATCCCGAAAGGAGTTCAGGGCACACGCACGC
DX-I-91	CTTGCGGAGTCGGTACCGCTTAATGCCATCGTAGACGGGATGC
DX-I-92	CTTATCCGTACCTAGAACCCGCTAGCAAAGAATGAGATTTGG
DX-I-93	GACGACATTTCGCGCAAGAAATGCCTTTATTCCATTCATATG
DX-I-94	ATGCGGGCGATCAACCCTCAGTAACGCCGCTTTAACGCGAAG
DX-I-95	CTGAACGCCACGGGATTGCCCGCAGTAAACCGTAGCTCTGGTT
DX-I-96	AACTGTAGACGTAGCCCACCTATTAGGGCGAACTCCCTTTAC
DX-I-97	ATTAATCGGGCCACCCATTTAGTCAGGAGTAGGCTCCGTGTC
DX-I-98	CGGACCCTTAAGACCAAATCTCAACGCCGCTAGCACTCGTAA
DX-I-99	ATAGACCACGTGTCCTAGCCCGGACTGAGTTCGAGCGTTTGAG
DX-I-100	GCCCACTTCCCGGTACGCTGCGGAGCCGATGAGCATATGTGGG
DX-I-101	CCTTAATGCCCTTGTGACCTCTGGAGTACATAATTGTGCATTT
DX-I-102	TTTCGGACCTACCAGAGCGAAGGAGACACGCTTACTAGGGCTG
DX-I-103	CGGTAGTCCTCAGAAAGGAAGCGGACTCAGCGCTTTCGGGATG
DX-I-104	CGCGCCGTTTCAGCCCTAGTAATCTATCCACTACCGTCTAAC
DX-I-105	ATTAGAGAATAGCGTGTCTCCTTCGCTCTGGGTATATAACCG
DX-I-106	AGGATATCTTCGGCTCTCGACTAGGTCCGAAACCAAATCTCA
DX-I-107	TTCTTTGCTAGAAATGTCGTCCATATGAATGGCTGAGGGTTGA
DX-I-108	TCGCCCGCATAAATGCACAATGAACGATACAGCCGGAAGCTG
DX-I-109	TCCTTGATGCTATGTACTCCAGAGGTCACAATATAATATGGT
DX-I-110	TTGGCATCCCAACGGCAGGGCGGGCATTAAGGGTAAAGGGAG
DX-I-111	TTCGCCCTAATCCCGATTAATGACACGGAGCCAGATTTGGTCT
DX-I-112	TAAGGGTCCGCCCACATATGCTAATGAATCACCAATTACGTG
DX-I-113	AAGAGTGGGTTCATCGGCTCCGCAGCGTACCCTCCAGCCGAG
DX-I-114	TTTTTTTTTTGCTTGAGAAGCGGGAAGTGGGCTTTTTTTTTT
DX-I-115	CGAGGGAACGACTGAATATTGAACCCTCAAGCCAGCTCGGGAG
DX-I-116	GAACCTTCGTCATCTGAAACACACAACTCGTTAAGCCGGGTGT
DX-I-117	GATGACACGCACCTAAGCTTACCCTCGATCTCCAACCTCCAGA
DX-I-118	TTTTTTTTTTCTGGAGGTTGAGGTTCGTTTATTTTTTTTTT
DX-I-119	CTATCGCCAAGAGATCGAGGGTAAGCTTAGGGCCTGGTACTG
DX-I-120	GCAGTATAGTCTGCGTCTCCATGCGTGTCATCGTTAGACGGT
DX-I-121	AGTGGATAGATATTCTCTAATCGGTTATATACGTCGAGAGCCG
DX-I-122	AAGATATCCTACACCCGGCTTTGAAATCTAGGTGATTTGCGC
DX-I-123	CTAAAGTTGTAACGAGTTGTGTGTTTCAGATCAAATACTCCT
DX-I-124	GAAGGTCACCAGCGCACGAGAGACGAAGGTTCCAGCTTCCGG
DX-I-125	CTGTATCGTTCGCATCAAGGAACCATATTATAGCCCTGCCGTT
DX-I-126	GGGATGCCAACTCCCGAGCTGCGTGTCACCGCCGTCAACGAT
DX-I-127	GTTAAGCCCTGCTTGAGGGTTCAATATTCAGCCACGCCTTAT
DX-I-128	TGCGGCGCAGTCCACGAAGTGTCGTTCCCTCGCACGTAATTG
DX-I-129	GTGATTCATTAACCCACTCTTCTCGGCTGGAGGCTTCTCAAGC
DX-I-130	TCGTTGCTTTGGCCGAAGATCTAGACGACGCCCGACGGGCCTG
DX-I-131	TCCAAGGGTGTCGCGTATCCGCAGTCCGGTCAATGGTTACCGC
DX-I-132	AGAGTACTAGGCCCTGGCTTCTGGGTCGTAGATGGAGTCCCTC
DX-I-133	TAAACGAACCTTTGGCGATAGCAGTACCAGGCTGGAGACGCAG

DX-I-134	ACTATACTGCGAGGGACTCCAACCCATGCCCTGAGTTAACAT
DX-I-135	AAGTAAGACGTCTACGACCCAGAAGCCAGGGCGACCCTTTGC
DX-I-136	GCTGACCTGGTTCCCTTCTTTCCTAGTACTCTGCGCAAATCA
DX-I-137	CCTAGATTTCAACAACTTTAGAGGAGTATTTGTCTCGTGCGCT
DX-I-138	GGTGACCTTCGCGGTAACCATCCTTCGCAGGCGACGATACAC
DX-I-139	TTTACATTGGTGACCGGACTGCGGATACGCGACAGTTACACA
DX-I-140	TCTTAAGAAATATCAGGACCCACACCCTTGGAATCGTTGACG
DX-I-141	GCGGTGACACGAGGGCTTAACATAAGGCGTGGCACTTCGTGGA
DX-I-142	CTGCGCCGCACAGGCCCGTCGGCAACGCAAATGTCACGGTGC
DX-I-143	CTAGGGCTGCGGCGTCGTCTAGATCTTCGGCCCGCCCGGCGC
DX-I-144	TTTTTTTTTTCGTCACTACAGCAAAGCAACGATTTTTTTTTT
DX-I-145	ACGTACAGGGCGTAATTGCTTCGGCGCGCGATGGCTGACACAG
DX-I-146	GGTCCCTGAAACACTTAATGTTTGGACAGCATGTTACCGCCTT
DX-I-147	GCGAACCTGTTGCCGGTGCACGACAGGATCTGCCCGAAATTGG
DX-I-148	TTTTTTTTTTCCAATTTCGGGCGGTCCTTCGCTTTTTTTTTT
DX-I-149	GGCAGACCATCAGATCCTGTCGTGCACCGGCCACAACGCGTG
DX-I-150	TCGCCTGCCCTCCTATCTACCAACAGGTTCGCATGTTAACTC
DX-I-151	AGGGCATGGGTCGTCTTACTTGCAAAGGGTCGAAAGAAGGGAA
DX-I-152	CCAGGTCAGCAAGGCGGTAACCGTTACAGGCTCCAATAACAA
DX-I-153	CACTTCCCTCATGCTGTCCAAACATTAAGTGTTGGTTATAGG
DX-I-154	CACGGCAGGATTATTTAGTAGTTTCAGGGACCGTGTATCGTC
DX-I-155	GCCTGCGAAGGCCAATGTAAATGTGTAACTGTGGGTCCTGATA
DX-I-156	TTTCTTAAGACTGTGTCAGCCGAATCGCCTGTTTAGACGCCA
DX-I-157	CTCGAGCGAGATCGCGCGCCGAAGCAATTACTCTCCTAGGTA
DX-I-158	TGTTTGCCGCAATAGACAATAGCCCTGTACGTGCACCGTGAC
DX-I-159	ATTTGCGTTGCGCAGCCCTAGGCGCCGGGCGGCTGTAGTGACG
DX-I-160	GTACCTCGCCCTCAAAGAAAGCATTTGTTACACATAGTTATCG
DX-I-161	AATACAACGGATGCAATCCCAAAGCTCTCCTGGATCGCCGTGA
DX-I-162	TTCCACAAAGGGTTCTCAGCTTGTCGTATTTCTGCAGAGATAC
DX-I-163	GCGAAGGACCGATGGTCTGCCCACGCGTTGTGGGTAGATAGGA
DX-I-164	GGGCAGGCGAGTATCTCTGCA
DX-I-165	GAAATACGACAAGCTGAGAAC
DX-I-166	CCTTTGTGGAATTGTTATTGG
DX-I-167	AGCCTGTAACGGAGGGAAGTGCCTATAACCAACTACTAAATAA
DX-I-168	TCCTGCCGTGTCACGGCGATC
DX-I-169	CAGGAGAGCTTTGGGATTGCA
DX-I-170	TCCGTTGTATTTGGCGTCTAA
DX-I-171	ACAGGCGATTCCTCGCTCGAGTACCTAGGAGATATTGTCTATT
DX-I-172	GCGGCAAACACGATAACTATG
DX-I-173	TGTAACAAATGCTTTCTTTGA
DX-I-174	GGGCGAGGTACTTTTTTTTTT

DX-II(10bp11bp10bp)	
NAME	SEQUENCE
DX-II-1	TGTGCATCCCTAGAAGGTTGAAACGGATCAA
DX-II-2	TTGCACGCGACTCGTACCTAG
DX-II-3	CGAGAGCAAAT
DX-II-4	TAATCGATGCGAGGTTCGTTT
DX-II-5	AACGCACGCACGTGTGCCTACTGACACAAGA
DX-II-6	GAAGAGGGTCGCGTGCTGCGC
DX-II-7	AGGACGTACAA
DX-II-8	AGCTACCCTCGGCAGTTGTTA
DX-II-9	ATTGGTTGTACCACGACTAATTGACACAAGA
DX-II-10	TGAAGACCCACCCTGGAAGGC
DX-II-11	TCCCGTCTGAT
DX-II-12	TAATCGGTCTTTTTTTTTTTT
DX-II-13	AGACCGATTAATCAGACGGGAGCCTTCCAGG
DX-II-14	GTGGGTCTTCATCTTGTGTCAGAACACGGTGGTAGAGCGTCT
DX-II-15	ATTAGTCGTGGCTCCCGGACCG
DX-II-16	CCTGGTCGTCCGGAAACGTAGTACAACCAATTAACAACTGCC
DX-II-17	GAGGGTAGCTTTGTACGTCCTGCGCAGCACG
DX-II-18	CGACCCTCTTCTCTTGTGTCACAGGCTGCCGTATCCCGCATC
DX-II-19	GTAGGCACACGTAAACAGTCTG
DX-II-20	ACGAATTCAGATACCGGGAATTGCGTGCGTTAAACGAACCTC
DX-II-21	GCATCGATTAATTTGCTCTCGCTAGGTACGA
DX-II-22	GTCGCGTGCAATTGATCCGTTGTTGTTGACCTGCAGGTAGGA
DX-II-23	TCAACCTTCTACAAGATGGTCG
DX-II-24	TTTTTTTTTTGGAGCACCACGGGATGCACATTTTTTTTTT
DX-II-25	CTCATATGAAAGAGGGAGATACGATGTCCAC
DX-II-26	ACCTATAGTGTGCGAATCGAGTACAACAGCT
DX-II-27	GGAGCACTGAAGGAGAATACGCTGGCTGCGA
DX-II-28	TTTTTTTTTTTCGCAGCCAGCTACTAAGTATTTTTTTTTTT
DX-II-29	CGTATTCTCCTGCCGTTGCGGA
DX-II-30	GAGACTAGGACCGGCATCAGCTCAGTGCTCCAGACGCTCTAC
DX-II-31	CACCGTGTTCCGGTCCGGGAGCTACGTTTCC
DX-II-32	GGACGACCAGGAGCTGTTGTAAATAGGGCACTTGGCGTTTGT
DX-II-33	CTCGATTCGCAGGCGAACTGTA
DX-II-34	CACCGGGCTCCGGCGTATTGCCACTATAGGTGATGCGGGATA
DX-II-35	CGGCAGCCTGCAGACTGTTTAATTCCCGGTA
DX-II-36	TCTGAATTCGTGTGGACATCGGAAATGCCCTGGTAAAGATTA
DX-II-37	TATCTCCCTCTCTGCATACGAG
DX-II-38	CGTTAAGACTCCCTGTCGGTCTTCATATGAGTCCTACCTGCA
DX-II-39	GGTCAACAACCGACCATCTTGGTGGTGCTCC
DX-II-40	GTCATGGGAAACCGTCGGCCGCCAATTTCGA
DX-II-41	ACGGAAAGCCGATACGATTAGCCTGTTAATG
DX-II-42	AATGAACGCCCACAATGTGCAGGCTTATGTA
DX-II-43	TACTTAGTAGTCCGCAACGGCGCTGATGCCG

DX-II-44	GTCCTAGTCTCTACATAAGCCTTGTCCGTGGCCGCTCCGCGT
DX-II-45	TGCACATTGTGACGACGTGTGC
DX-II-46	AGTCTTTGGGAATTCTCCTAAGGCGTTCATTACAAACGCCAA
DX-II-47	GTGCCCTATTTACAGTTCGCCGCAATACGCC
DX-II-48	GGAGCCCGGTGCATTAACAGGGCCGAGCTAGACCTAAGAGTT
DX-II-49	CTAATCGTATCCTAAGTCGCGA
DX-II-50	TTGGATCTACTGACGTCCGTTGGCTTTCCGTTAATCTTTACC
DX-II-51	AGGGCATTTCCTCGTATGCAGGACCGACAGG
DX-II-52	GAGTCTTAACGTCGAAATTGGTCCAGCCCGGCTACTTACCCT
DX-II-53	CGGCCGACGGTGTGTTGTAGCT
DX-II-54	TTTTTTTTTTTTCTGGAAGAATTCCCATGACTTTTTTTTTTT
DX-II-55	CCAAAGGTAAGGGCCTCCGCCCTACTCCGCC
DX-II-56	GTAGGACGGCGTTGCCTACCTTAACTGCGGG
DX-II-57	CCTCCTTAGATGTTTAGGAACGATGGGCACA
DX-II-58	TTTTTTTTTTTTGTGCCCATCCGCCGTTATATTTTTTTTTTT
DX-II-59	GTTCCTAAACACTCGATAATCT
DX-II-60	TACCGCATTGAATGTGCATTCTCTAAGGAGGACGCGGAGCGG
DX-II-61	CCACGGACAAGCACACGTCGTTTAGGAGAAT
DX-II-62	TCCCAAAGACTCCCGCAGTTAGTCGCCCTTCTGTTTAAGCGT
DX-II-63	AGGTAGGCAACATTAACTGAAT
DX-II-64	CCCGTGTGGTGCCACGGCTAAGCCGTCCTACAACTCTTAGGT
DX-II-65	CTAGCTCGGCTCGCGACTTAGAACGGACGTC
DX-II-66	AGTAGATCCAAGGCGGAGTAGCTGCACGCTCAGGTTAGAATA
DX-II-67	GGCGGAGGCCCTAGATATACTG
DX-II-68	ATTTACACGATGGCCGTAGTCTTACCTTTGGAGGGTAAGTAG
DX-II-69	CCGGGCTGGAAGCTACAACACTTCTTCCAGA
DX-II-70	AGAGGGTTCCCTTAGGACTCTGATTCTTTCG
DX-II-71	ATGTAATGCCTTAATGCTGGTAAACTCCCAT
DX-II-72	GGCTACTGAAGTAGCCTGTCCAACTATCAGT
DX-II-73	TATAACGGCGAGATTATCGAGGAATGCACAT
DX-II-74	TCAATGCGGTAACTGATAGTTTGCCACTGCCAGCTTAGTTCA
DX-II-75	GGACAGGCTACACTAAGATTCC
DX-II-76	GCCCTCATAGAGTTGACGATATTCAGTAGCCACGCTTAAACA
DX-II-77	GAAGGGCGACATTCAGTTAATTTAGCCGTGG
DX-II-78	CACCACACGGGATGGGAGTTTCCTACTGCACGCACACGGGCA
DX-II-79	ACCAGCATTAAACAACATTGAT
DX-II-80	TCGCGACAACACGTGAACTCGGGCATTACATTATTCTAACCT
DX-II-81	GAGCGTGCAGCAGTATATCTAGACTACGGCC
DX-II-82	ATCGTGTAAATCGAAAGAATCCGGAGTGATGGACTATCCTCG
DX-II-83	AGAGTCCTAAGTGTAGCGACTT
DX-II-84	TTTTTTTTTTTGGGAAGTCTTGGAACCCTCTTTTTTTTTTTT
DX-II-85	CACTGGTATTCTGACCGCCACTTGGCAGTTG
DX-II-86	GGAGCCCTACGTTTGCTTTAATGCGAAAGGC
DX-II-87	GGCTGACGGTCCGTGTGGACTTTAAGAATAG
DX-II-88	TTTTTTTTTTTCTATTCTTAATTGGGTATGATTTTTTTTTTT

DX-II-89	AGTCCACACGGCGGGCTTGTGA
DX-II-90	CAGGGCGTGACGGGTAAGTAAACCGTCAGCCTGAACTAAGCT
DX-II-91	GGCAGTGGCAGGAATCTTAGTTATCGTCAAC
DX-II-92	TCTATGAGGGCGCCTTTCGCACGGACGACGTTAGTCAGTAAA
DX-II-93	TTAAAGCAAACGACTGTGGAAT
DX-II-94	GTCGAAACCTTGACGGTAAAGGTAGGGCTCCTGCCCGTGTGC
DX-II-95	GTGCAGTAGGATCAATGTTGTCGAGTTCACG
DX-II-96	TGTTGTCGCGACAACTGCCAAATTGGATGTCCAAATCGCTAT
DX-II-97	GTGGCGGTCAGTTTAAGTTTCT
DX-II-98	CGAGTGTTTAGGGCTTCTTCCAATACCAGTGCGAGGATAGTC
DX-II-99	CATCACTCCGAAGTCGCTACAAAGACTTCCC
DX-II-100	ATCGTTGCGAACCAGGTCGAGGTTCCGCCGG
DX-II-101	GCCAAAGGTGGGCGACGCCCTGGGTAAGATG
DX-II-102	TTGGCGCGTTCTGGGACCGCTTAGTGCGCAG
DX-II-103	TCATACCCAATCACAAGCCCGTTACTTACCC
DX-II-104	GTCACGCCCTGCTGCGCACTAGTTCACGTACCGGGTGGACAA
DX-II-105	AGCGGTCCCAGGAACGGTTATT
DX-II-106	GATGTTAAATGCCCTAAAGGAAACGCGCCAATTTACTGACTA
DX-II-107	ACGTCGTCCGATTCCACAGTCCTTTACCGTC
DX-II-108	AAGGTTTCGACCATCTTACCCATCACCTTACGACAGCCACAG
DX-II-109	AGGGCGTCGCCCATTAACTCTT
DX-II-110	TTCTTAATTTAAGACGACCACCACCTTTGGCATAGCGATTTG
DX-II-111	GACATCCAATAGAAACTTAAAGGAAGAAGCC
DX-II-112	CTAAACACTCGCCGGCGGAACGTCGAATGCGAGTCAGGAGCT
DX-II-113	CTCGACCTGGTCTATGCTTCCA
DX-II-114	TTTTTTTTTTCGCAACAGATTCGCAACGATTTTTTTTTTT
DX-II-115	ATTTATGTGGTCTGGTTATCCAACAAGGTAT
DX-II-116	CCCTACCGCCAACTGCGCGATGTCTTGAATA
DX-II-117	TCCGTAGGCATTAAAGAGGGTCCGAACCATT
DX-II-118	TTTTTTTTTTAATGGTTCGGGCTAGGATCGTTTTTTTTTT
DX-II-119	ACCCTCTTTAATGCTATGATTA
DX-II-120	TTGAGTTAAAGATGATGTTCCTGCCTACGGATTGTCCACCCG
DX-II-121	GTACGTGAACAATAACCGTTCTCCTTTAGGG
DX-II-122	CATTTAACATCTATTCAAGACATCGTATATCTAGTTCCAAAG
DX-II-123	ATCGCGCAGTTAGACGGCTGCG
DX-II-124	GGCGTGAGGGCTCTGTGAAGCGGCGGTAGGGCTGTGGCTGTC
DX-II-125	GTAAGGTGATAAGAGTTAATGGTGGTCGTCT
DX-II-126	TAAATTAAGAAATACCTTGTTTGCAATATAGGACTTAGCGCC
DX-II-127	GGATAACCAGATTGGAGACGTG
DX-II-128	GGCCAGGCACCGTTCAACTTGCCACATAAATAGCTCCTGACT
DX-II-129	CGCATTCGACTGGAAGCATAGATCTGTTGCG
DX-II-130	GTGGAAATGTGACCCGGAATGCGCCCTCTTT
DX-II-131	CGCAAGCGCCATGAGAAGATTTAGGCTGGGC
DX-II-132	CCTACATGACATCGGGCTCTCATATGCCCTC
DX-II-133	CGATCCTAGCTAATCATAGCAGGAACATCAT

DX-II-134	CTTTAACTCAAGAGGGCATATACCGAATGGACGTACACAGTG
DX-II-135	GAGAGCCCGATGGTGTGTGTCA
DX-II-136	TGCGCCGTTGTCCAGTCGGCAGTCATGTAGGCTTTGGAACTA
DX-II-137	GATATACGATCGCAGCCGTCTGCTTCACAGA
DX-II-138	GCCCTCACGCCGCCCAGCCTAGTGCAACGCTTCTCCCGGCTA
DX-II-139	AATCTTCTCATTCAATCATACG
DX-II-140	AAAGTTAGAGTCACCAACTTCGGCGCTTGCGGGCGCTAAGTC
DX-II-141	CTATATTGCACACGTCTCCAACAAGTTGAAC
DX-II-142	GGTGCCTGGCCAAAGAGGGCGACAGCTGCACATGTTTACGGA
DX-II-143	CATTCCGGGTCGGTTCACTCGT
DX-II-144	TTTTTTTTTTGACAAACCGTACATTTCCACTTTTTTTTTTT
DX-II-145	TTGGCCCATTTCGACTCTAGGTTTCTCTATT
DX-II-146	CAAGAGGGAGGGAAAGACGATCGAAGCCTGA
DX-II-147	ATAACTGAGGCTGAAGGGCGGAGACATGTCC
DX-II-148	TTTTTTTTTTGGACATGTCTCGGAGAGGAATTTTTTTTTT
DX-II-149	CCGCCCTTCAGAACGTCATACT
DX-II-150	GTGAGTATGTGAATGGTGTGACCTCAGTTATCACTGTGTACG
DX-II-151	TCCATTCGGTTGACACACACCTGCCGACTGG
DX-II-152	ACAACGGCGCATCAGGCTTCGACCCTAATCGGGAGGTGGTTC
DX-II-153	ATCGTCTTTCCAGGCGACTTTA
DX-II-154	CTGAAACAATGCGCGTATAAGCTCCCTCTTGTAGCCGGGAGA
DX-II-155	AGCGTTGCACCGTATGATTGAGAAGTTGGTG
DX-II-156	ACTCTAACTTTAATAGAGAAAGAAAGAACAGGGCAGCCGGAT
DX-II-157	CCTAGAGTCGAATCGCAAGTTG
DX-II-158	GGGTGTTAGAAAGATCCGTGGAATGGGCCAATCCGTAAACAT
DX-II-159	GTGCAGCTGTACGAGTGAACCACGGTTTGTC
DX-II-160	CGGAGCTAAAGGACTGTGCGGACGGTTTACT
DX-II-161	GACAACCAGCGCTAACTTACTCATCTATTAC
DX-II-162	TTAGGCATGTTCACCACTTGACCTATGCGTC
DX-II-163	TTCCTCTCCGAGTATGACGTTTCACACCATT
DX-II-164	CACATACTCACGACGCATAGG
DX-II-165	TCAAGTGGTGA
DX-II-166	ACATGCCTAAGAACCACCTCC
DX-II-167	CGATTAGGGTTAAAGTCGCCTCTTATACGCG
DX-II-168	CATTGTTTCAGGTAATAGATG
DX-II-169	AGTAAGTTAGC
DX-II-170	GCTGGTTGTCATCCGGCTGCC
DX-II-171	CTGTTCTTTCCAACTTGCGATCCACGGATCT
DX-II-172	TTCTAACACCCAGTAAACCGT
DX-II-173	CCGCACAGTCC
DX-II-174	TTTAGCTCCGTTTTTTTTTT

2x2(11bp10bp)	
NAME	SEQUENCE
2x2-1	CCTTCAAGGGCCCAAGACAGACTTTTTTTTTT
2x2-2	GTCTGTCTTGGCAGTAAGAGCACGTCAGCCAG
2x2-3	CATAGAATCCAGCCCTTGAAGGTTTTTTTTTT
2x2-4	TGTTGGTGTAGAAAGAGAGAACTGTATATCAT
2x2-5	GTGGGCCGCGGACCAGGTTCATTTTTTTTTTT
2x2-6	ATGAACCTGGTTTACGACTGACTTTTTTTTTT
2x2-7	AGGGCGGGCGGCCGCGGCCCACCTGGCTGACG
2x2-8	TGCTCTTACTGTGGATTCTATGATGATATACA
2x2-9	GTTCTCTCTTTAGCTCCCAAACCTTGCCTTTA
2x2-10	AACTGCATGGACTACACCAACATTTTTTTTTT
2x2-11	GGGACATGGATTGTTTGACCTCAATGCATACG
2x2-12	GTCAGTCGTAACCGCCCGCCCTCGTATGCATT
2x2-13	GAGGTCAAACAGAGTCCGCTATTTTTTTTTTT
2x2-14	GCCATGGTTTCATCCATGTCCCTAAAGGCAAG
2x2-15	GTTTGGGAGCTTCCATGCAGTTTTTTTTTTTT
2x2-16	ATAGCGGACTCGAAACCATGGCTTTTTTTTTT

3x3(11bp10bp)	
NAME	SEQUENCE
3x3-1	AGAGACGCACAttttTCAGTTTCTGTTCACGTCCTT
3x3-2	ACAGAAACTGATGTGCGTCTCTTAGTGCTTAA
3x3-3	TGAAAGACCGGttttAGTGTGGTAAAGGCATGGACT
3x3-4	TTTACCACACTCCGGTCTTTCATTTAGGCTGG
3x3-5	GTGGAACTTTGttttCTCTATTGGGCAATGCTAATA
3x3-6	GCCCAATAGAGCAAAGTTCCACTGCGAAATCG
3x3-7	CATATAAACAAttttGGTATCCGTCAGGTTCGGCCT
3x3-8	TGACGGATACCTTGTTTATATGCAGTCACCAG
3x3-9	CTTGAGCCATCttCCTGCCAAACGCCAGCCTAAA
3x3-10	CGTTTGGCAGGGAAGATGATAAGGGTGCCGTT
3x3-11	TTATCATCTTCGATGGCTCAAGAAGGACGTGA
3x3-12	CGCTTGGTCGTttGGCGGACCTACCGATTTCGCA
3x3-13	GTAGGTCCGCCCGAGAACGTAACCCTCCCATT
3x3-14	TTACGTTCTCGACGACCAAGCGAGTCCATGCC
3x3-15	AGGGCTTCAACttTCGGAATTTGTCTGGTGACTG
3x3-16	ACAAATTCCGACATAACTAGCCGGGTACGATC
3x3-17	GGCTAGTTATGGTTGAAGCCCTTATTAGCATT
3x3-18	GTTAATTGTATttTCAGCGTAGCTTTAAGCACTA
3x3-19	AGCTACGCTGAGAGGCGCAAGTAATCCTGGAT
3x3-20	ACTTGCGCCTCATACAATTAACAGGCCGAACC
3x3-21	CCCGCTCGTTAGAATCTGCTGCAACGGCACCC
3x3-22	GCAGCAGATTCTAGGAGCTGTGAATGGGAGGG
3x3-23	CACAGCTCCTAGACTTACAGTAGATCGTACCC
3x3-24	TACTGTAAGTCTAACGAGCGGGATCCAGGATT

J4-IA(11bp10bp)	
NAME	SEQUENCE
J4-IA-1	CCTTCAAGGGCttCCAAGACAGACtttttttttt
J4-IA-2	GTCTGTCTTGGttCAGTAAGAGCACGTCAGCCAG
J4-IA-3	CATAGAATCCAttGCCCTTGAAGGtttttttttt
J4-IA-4	TGTTGGTGTAGttAAAGAGAGAACTGTATATCAT
J4-IA-5	CCTTCAAGGGCttCCAAGACAGACtttttttttt
J4-IA-6	ATGAACCTGGTttTTACGACTGACTAATATAACA
J4-IA-7	AGGGCGGGCGGttCCGCGGCCCACCTGGCTGACG
J4-IA-8	TGCTCTTACTGttTGGATTCTATGATGATATACA
J4-IA-9	GTTCTCTCTTTttAGCTCCCAAACCTTGCCTTTA
J4-IA-10	AACTGCATGGAttCTACACCAACAtttttttttt
J4-IA-11	ATGCTTGAATTttTACAAACTATTTGACCATCCA
J4-IA-12	GGGACATGGATttTGTTTGACCTCAATGCATACG
J4-IA-13	GATCAACGCATttGTGACCTTAATtttttttttt
J4-IA-14	ATTAAGGTCACttAGCAACTTAGCTACTTTGGAG
J4-IA-15	TTGAACTTCATttATGCGTTGATCTGTTATATTA
J4-IA-16	GTCAGTCGTAAttCCGCCCGCCCTCGTATGCATT
J4-IA-17	GAGGTCAAACAttGAGTCCGCTATGTTTGGCCCT
J4-IA-18	GCCATGGTTTCttATCCATGTCCCTAAAGGCAAG
J4-IA-19	GTTTGGGAGCTttTCCATGCAGTTTGGATGGTCA
J4-IA-20	AATAGTTTGTAttATTCATCCGTCAGCCGTTGCT
J4-IA-21	CACGGCAGTCAttAATTCAAGCATtttttttttt
J4-IA-22	GGGACATAGCGttCCTATGTCCGTAGCCCTCGGT
J4-IA-23	GTCAGACATGGttCGACTGAAGCGACCCGTGTTC
J4-IA-24	ATGTTGGTGAGttAAGATAGTCGGACGGTGTGGA
J4-IA-25	GGATCATCGCAttTCAGTAGACTCtttttttttt
J4-IA-26	GAGTCTACTGAttAGGAGGATCGAAGGGCGCGGT
J4-IA-27	CTCTCGTGGGCttTGCGATGATCCCTCCAAAGTA
J4-IA-28	GCTAAGTTGCTttATGAAGTTCAATCCACACCGT
J4-IA-29	CCGACTATCTTttAAGATGGGCCGACGTGAACAT
J4-IA-30	GCATTTAGATCttCTCACCAACATAGGGCCAAAC
J4-IA-31	ATAGCGGACTCttGAAACCATGGCGAACACGGGT
J4-IA-32	CGCTTCAGTCGttGCCACATCCTTCGATTCCCGT
J4-IA-33	CAAGTGAATAAttCCATGTCTGACAGCAACGGCT
J4-IA-34	GACGGATGAATttTGACTGCCGTGACCGAGGGCT
J4-IA-35	ACGGACATAGGttCCCTTGCCGACTATGGGCTCG
J4-IA-36	CACTCAGATGGttCGCTATGTCCCtttttttttt
J4-IA-37	GGCTCGGTAGTttATTGCGCTTTCACCGTCAATT
J4-IA-38	ACCGTTGCTTTttATCCTGCGAGCACCCTGTCTG
J4-IA-39	TAAAGTTCCCTttTGTATATGTTTATAGCACGTA
J4-IA-40	AACCCGCGTACttTGCTCTACACTTTGTCAATTA
J4-IA-41	GTAAAGATATTttCCGCGACGGTTtttttttttt
J4-IA-42	AACCGTCGCGGttATCTTGCAACGTTGCTTGCAG
J4-IA-43	CGAGCGGTTAGttAATATCTTTACACCGCGCCCT

J4-IA-44	TCGATCCTCCTttGCCCACGAGAGTAATTGACAA
J4-IA-45	AGTGTAGAGCAttGAATTCCTCGGTTTCGTTCCG
J4-IA-46	TCACACGTCCAttGTACGCGGGTTATGTTCACGT
J4-IA-47	CGGCCCATCTTttGATCTAAATGCTACGTGCTAT
J4-IA-48	AAACATATACAttTCCCTTACCAGGTCGACGTCG
J4-IA-49	GGCGCGCCAACttAGGGAACTTTAACGGGAATCG
J4-IA-50	AAGGATGTGGCttTTATTCACTTGCAGACAGGGT
J4-IA-51	GCTCGCAGGATttAATTCAGGTCAACGCAGGTCA
J4-IA-52	CGGGCTTTAACttAAAGCAACGGTCGAGCCCATA
J4-IA-53	GTCGGCAAGGGttCCATCTGAGTGAATTGACGGT
J4-IA-54	GAAAGCGCAATttGGGATGCCGCTCGTTCCGGCT
J4-IA-55	AGTTGTCTCCTttACTACCGAGCCtttttttttt
J4-IA-56	AGATTTGCAGTttTGTGGGCGATGAGCACCACCG
J4-IA-57	CAGGGAATGGAttCTGTCGCATTAGCTCCAGTGA
J4-IA-58	TCTCACCGCACttTGGACAAATTTAGGCCTGCAG
J4-IA-59	CTCGGCGAGACttTCAGAACCCATGTTCCCGATC
J4-IA-60	GCTAAATGGCTttTTCCATGAAGTTATATGCTAC
J4-IA-61	CGTCTTTATATttTCCGACGTAAGtttttttttt
J4-IA-62	CTTACGTCGGAttCTATGTAATATtttttttttt
J4-IA-63	TCAACGAGGACttATATAAAGACGCTGCAAGCAA
J4-IA-64	CGTTGCAAGATttCTAACCGCTCGGTAGCATATA
J4-IA-65	ACTTCATGGAAttTGTTCTTCGCAGTCGACGGAG
J4-IA-66	TAAAGTAGAGTttAGCCATTTAGCCGGAACGAAA
J4-IA-67	CCGAGGAATTCttTGGACGTGTGAGATCGGGAAC
J4-IA-68	ATGGGTTCTGAttTCTGTAAAGACGCTAGGAGCA
J4-IA-69	TGCTTCCCTCAttGTCTCGCCGAGCGACGTCGAC
J4-IA-70	CTGGTAAGGGAttGTTGGCGCGCCCTGCAGGCCT
J4-IA-71	AAATTTGTCCAttCACCTAGCTACTGGCGGGCAC
J4-IA-72	TGAAACGATGTttGTGCGGTGAGATGACCTGCGT
J4-IA-73	TGACCTGAATTttGTTAAAGCCCGTCACTGGAGC
J4-IA-74	TAATGCGACAGttATGGGCTTTAAGGCTATCGTA
J4-IA-75	TTAACACCTGCttTCCATTCCCTGAGCCGGAACG
J4-IA-76	AGCGGCATCCCttAGGAGACAACTCGGTGGTGCT
J4-IA-77	CATCGCCCACAttACGCTGAATAGCGCTGGAGTC
J4-IA-78	GTCACGAACTAttACTGCAAATCTtttttttttt
J4-IA-79	GTTAACAGATCttTGGTGTGGTATGATGTTGTGG
J4-IA-80	TCGTCCACGTGttTTCAATCTGATGGTTAACGCT
J4-IA-81	AATGGTACAACttGGTTAGCCGCGGCTATATTGG
J4-IA-82	CGGAATGATAGttGTCGGTTACTGGTAGGCAGTA
J4-IA-83	GATCCCAACCGttCCCTCCCTAAATCGTCAAACA
J4-IA-84	ATATTACATAGttGTCCTCGTTGATGTTTGACGA
J4-IA-85	TTTAGGGAGGGttCTAGTCAGAGAtttttttttt
J4-IA-86	GTTAGCTGTGCttCGGTTGGGATCCTCCGTCGAC
J4-IA-87	TGCGAAGAACAttACTCTACTTTATACTGCCTAC
J4-IA-88	CAGTAACCGACttACGGTCCGCAGGTCAGTATCA

J4-IA-89	CGGGCAGAGAGttCTATCATTCCGTGCTCCTAGC
J4-IA-90	GTCTTTACAGAttTGAGGGAAGCACCAATATAGC
J4-IA-91	CGCGGCTAACCttCTACTTGATATTCGGATAGAT
J4-IA-92	TCCGGCTCGAAttGTTGTACCATTGTGCCCGCCA
J4-IA-93	GTAGCTAGGTGttACATCGTTTCAAGCGTTAACC
J4-IA-94	ATCAGATTGAAttGGGCAAAGACCATGGTGATTC
J4-IA-95	TATATTCCTGCttCACGTGGACGATACGATAGCC
J4-IA-96	TTAAAGCCCATttGCAGGTGTTAACCACAACATC
J4-IA-97	ATACCACACCAttCGTGCTAGCCACTGACCAGCT
J4-IA-98	CTTGGTGACGGttGATCTGTTAACGACTCCAGCG
J4-IA-99	CTATTCAGCGTttTAGTTCGTGACttttttttt
J4-IA-100	GTCGCTGCAAAttTTGTGTTGTATAATATGTCAG
J4-IA-101	TCCCGTTGTCGttTCGGCATGCTATCCCTCGAAG
J4-IA-102	TCAGATGCCGAttTGATGGGTACGGTGCTCTAGC
J4-IA-103	GCATGGCCCTCttTACCGCCTTCGGAACAGTGGT
J4-IA-104	TCTCTGACTAGttGCACAGCTAACACCACTGTTC
J4-IA-105	CGAAGGCGGTAttTGATCTGGTCGttttttttt
J4-IA-106	AGTTGCTGGTGttGAGGGCCATGCTGATACTGAC
J4-IA-107	CTGCGGACCGTttCTCTCTGCCCGGCTAGAGCAC
J4-IA-108	CGTACCCATCAttTCATCCACTCCTTGCTTAATG
J4-IA-109	ATCTTACAAGTttTCGGCATCTGAATCTATCCGA
J4-IA-110	ATATCAAGTAGttTTCGAGCCGGACTTCGAGGGA
J4-IA-111	TAGCATGCCGAttATTTATAGGGCGCCTCTGATG
J4-IA-112	CGCCCTACATAttCGACAACGGGAGAATCACCAT
J4-IA-113	GGTCTTTGCCCttGCAGGAATATACTGACATATT
J4-IA-114	ATACAACACAAttTGCTGTGCCCTCAAAGGTATC
J4-IA-115	GCATATAGCGGttTTTGCAGCGACAGCTGGTCAG
J4-IA-116	TGGCTAGCACGttCCGTCACCAAGttttttttt
J4-IA-117	AACCCTGCTATttTAATTGCGCAGTGTTTAGGAT
J4-IA-118	ACCTGCGGATAttTCAAGAGGGCTTTGAGCTACC
J4-IA-119	GTTCGATGTCTttAGTTCGAACAGTTGGAAGGTC
J4-IA-120	CGACCAGATCAttCACCAGCAACTGACCTTCCAA
J4-IA-121	CTGTTCGAACTttCGGTGCCCTGTttttttttt
J4-IA-122	CCTGTCTAGCAttAGACATCGAACCATTAAGCAA
J4-IA-123	GGAGTGGATGAttACTTGTAAGATGGTAGCTCAA
J4-IA-124	AGCCCTCTTGAttCTGAACATGGCCCTTCGTCCG
J4-IA-125	TACTACAAATAttTATCCGCAGGTCATCAGAGGC
J4-IA-126	GCCCTATAAATttTATGTAGGGCGATCCTAAACA
J4-IA-127	CTGCGCAATTAttCCCTATATGCATCTAGTAAGT
J4-IA-128	CATCCATGATGttATAGCAGGGTTGATACCTTTG
J4-IA-129	AGGGCACAGCAttCCGCTATATGCttttttttt
J4-IA-130	TCCGACGTTACttATGGTCCCGCGGGCGGCACGT
J4-IA-131	CGCGCGACATAttATTCGGTATCTGTACTCAAAG
J4-IA-132	ACAGGGCACCGttTGCTAGACAGGCTTTGAGTAC
J4-IA-133	AGATACCGAATttCTCTGGTTGAAttttttttt

J4-IA-134	CGTTGAAGTTAttTATGTCGCGCGGACGAAGG
J4-IA-135	GCCATGTTCAGttTATTTGTAGTAACGTGCCGCC
J4-IA-136	CGCGGGACCATttGCAGGACTGGACATGTAGCGA
J4-IA-137	CCTACCGGGAAttGTAACGTCGGAACTTACTAGA
J4-IA-138	TGCATATAGGGttCATCATGGATGtttttttttt
J4-IA-139	AGGGCTTTATTttCGTAAATAGAGTGGACCTGTA
J4-IA-140	TTCAACCAGAGttTAACTTCAACGTACAGGTCCA
J4-IA-141	CTCTATTTACGttTGAAGCGATGCtttttttttt
J4-IA-142	GTAACTAATAGttAATAAAGCCCTTCGCTACATG
J4-IA-143	TCCAGTCCTGCttTTCCCGGTAGGtttttttttt
J4-IA-144	GCATCGCTTCAttCTATTAGTTACtttttttttt

J4-IB(11bp10bp)	
NAME	SEQUENCE
J4-IB-1	AAGCCTTCAAGGGCCCAAGACAGACGATGCACTGA
J4-IB-2	GTCTGTCTTGGttCAGTAAGAGCACGTCAGCCAG
J4-IB-3	CATAGAATCCAGCCCTTGAAGGCTTAAC
J4-IB-4	CATTGTTGGTGTAGAAAGAGAGAACTGTATATCAT
J4-IB-5	CCTTCAAGGGCttCCAAGACAGACtttttttttt
J4-IB-6	ATGAACCTGGTttTTACGACTGACTAATATAACA
J4-IB-7	AGGGCGGGCGGttCCGCGGCCCACCTGGCTGACG
J4-IB-8	TGCTCTTACTGttTGGATTCTATGATGATATACA
J4-IB-9	GTTCTCTCTTTttAGCTCCCAAACCTTGCCTTTA
J4-IB-10	AACTGCATGGACTACACCAACAATGTTA
J4-IB-11	TCGATGCTTGAATTTACAAACTATTTGACCATCCA
J4-IB-12	GGGACATGGATttTGTTTGACCTCAATGCATACG
J4-IB-13	GATCAACGCATttGTGACCTTAATtttttttttt
J4-IB-14	ATTAAGGTCACttAGCAACTTAGCTACTTTGGAG
J4-IB-15	TTGAACTTCATttATGCGTTGATCTGTTATATTA
J4-IB-16	GTCAGTCGTAAttCCGCCCGCCCTCGTATGCATT
J4-IB-17	GAGGTCAAACAttGAGTCCGCTATGTTTGGCCCT
J4-IB-18	GCCATGGTTTCttATCCATGTCCCTAAAGGCAAG
J4-IB-19	GTTTGGGAGCTttTCCATGCAGTTTGGATGGTCA
J4-IB-20	AATAGTTTGTAttATTCATCCGTCAGCCGTTGCT
J4-IB-21	CACGGCAGTCAAATTCAAGCATCGAGTA
J4-IB-22	ATCGGGACATAGCGCCTATGTCCGTAGCCCTCGGT
J4-IB-23	GTCAGACATGGttCGACTGAAGCGACCCGTGTTC
J4-IB-24	ATGTTGGTGAGttAAGATAGTCGGACGGTGTGGA
J4-IB-25	GGATCATCGCAttTCAGTAGACTCtttttttttt
J4-IB-26	GAGTCTACTGAttAGGAGGATCGAAGGGCGCGGT
J4-IB-27	CTCTCGTGGGCttTGCGATGATCCCTCCAAAGTA
J4-IB-28	GCTAAGTTGCTttATGAAGTTCAATCCACACCGT
J4-IB-29	CCGACTATCTTttAAGATGGGCCGACGTGAACAT
J4-IB-30	GCATTTAGATCttCTCACCAACATAGGGCCAAAC
J4-IB-31	ATAGCGGACTCttGAAACCATGGCGAACACGGGT
J4-IB-32	CGCTTCAGTCGttGCCACATCCTTCGATTCCCGT
J4-IB-33	CAAGTGAATAAttCCATGTCTGACAGCAACGGCT
J4-IB-34	GACGGATGAATttTGACTGCCGTGACCGAGGGCT
J4-IB-35	ACGGACATAGGttCCCTTGCCGACTATGGGCTCG
J4-IB-36	CACTCAGATGGCGCTATGTCCCGATGAT
J4-IB-37	ATTGGCTCGGTAGTATTGCGCTTTCACCGTCAATT
J4-IB-38	ACCGTTGCTTTttATCCTGCGAGCACCCTGTCTG
J4-IB-39	TAAAGTTCCCTttTGTATATGTTTATAGCACGTA
J4-IB-40	AACCCGCGTACttTGCTCTACACTTTGTCAATTA
J4-IB-41	GTAAAGATATTttCCGCGACGGTTtttttttttt
J4-IB-42	AACCGTCGCGGttATCTTGCAACGTTGCTTGCAG
J4-IB-43	CGAGCGGTTAGttAATATCTTTACACCGCGCCCT

J4-IB-44	TCGATCCTCCTttGCCCACGAGAGTAATTGACAA
J4-IB-45	AGTGTAGAGCAttGAATTCCTCGGTTTCGTTCCG
J4-IB-46	TCACACGTCCAttGTACGCGGGTTATGTTCACGT
J4-IB-47	CGGCCCATCTTtGATCTAAATGCTACGTGCTAT
J4-IB-48	AAACATATACAttTCCCTTACCAGGTCGACGTCG
J4-IB-49	GGCGCGCCAACttAGGGAACTTTAACGGGAATCG
J4-IB-50	AAGGATGTGGCttTTATTCACTTGCAGACAGGGT
J4-IB-51	GCTCGCAGGATttAATTCAGGTCAACGCAGGTCA
J4-IB-52	CGGGCTTTAACttAAAGCAACGGTCGAGCCCATA
J4-IB-53	GTCGGCAAGGGttCCATCTGAGTGAATTGACGGT
J4-IB-54	GAAAGCGCAATttGGGATGCCGCTCGTTCCGGCT
J4-IB-55	AGTTGTCTCCTACTACCGAGCCAATTGA
J4-IB-56	CCTAGATTTGCAGTTGTGGGCGATGAGCACCACCG
J4-IB-57	CAGGGAATGGAttCTGTCGCATTAGCTCCAGTGA
J4-IB-58	TCTCACCGCACttTGGACAAATTTAGGCCTGCAG
J4-IB-59	CTCGGCGAGACttTCAGAACCCATGTTCCCGATC
J4-IB-60	GCTAAATGGCTttTTCCATGAAGTTATATGCTAC
J4-IB-61	CGTCTTTATATttTCCGACGTAAGtttttttttt
J4-IB-62	CTTACGTCGGACTATGTAATATTGAGTT
J4-IB-63	TCAACGAGGACttATATAAAGACGCTGCAAGCAA
J4-IB-64	CGTTGCAAGATttCTAACCGCTCGGTAGCATATA
J4-IB-65	ACTTCATGGAAttTGTTCTTCGCAGTCGACGGAG
J4-IB-66	TAAAGTAGAGTttAGCCATTTAGCCGGAACGAAA
J4-IB-67	CCGAGGAATTCttTGGACGTGTGAGATCGGGAAC
J4-IB-68	ATGGGTTCTGAttTCTGTAAAGACGCTAGGAGCA
J4-IB-69	TGCTTCCCTCAttGTCTCGCCGAGCGACGTCGAC
J4-IB-70	CTGGTAAGGGAttGTTGGCGCGCCCTGCAGGCCT
J4-IB-71	AAATTTGTCCAttCACCTAGCTACTGGCGGGCAC
J4-IB-72	TGAAACGATGTttGTGCGGTGAGATGACCTGCGT
J4-IB-73	TGACCTGAATTttGTTAAAGCCCGTCACTGGAGC
J4-IB-74	TAATGCGACAGttATGGGCTTTAAGGCTATCGTA
J4-IB-75	TTAACACCTGCttTCCATTCCCTGAGCCGGAACG
J4-IB-76	AGCGGCATCCCttAGGAGACAACTCGGTGGTGCT
J4-IB-77	CATCGCCCACAttACGCTGAATAGCGCTGGAGTC
J4-IB-78	GTCACGAACTAACTGCAAATCTAGGAGT
J4-IB-79	GTTAACAGATCttTGGTGTGGTATGATGTTGTGG
J4-IB-80	TCGTCCACGTGttTTCAATCTGATGGTTAACGCT
J4-IB-81	AATGGTACAACttGGTTAGCCGCGGCTATATTGG
J4-IB-82	CGGAATGATAGttGTCGGTTACTGGTAGGCAGTA
J4-IB-83	GATCCCAACCGttCCCTCCCTAAATCGTCAAACA
J4-IB-84	TCAATATTACATAGGTCCTCGTTGATGTTTGACGA
J4-IB-85	TTTAGGGAGGGCTAGTCAGAGAATTTAA
J4-IB-86	GTTAGCTGTGCttCGGTTGGGATCCTCCGTCGAC
J4-IB-87	TGCGAAGAACAttACTCTACTTTATACTGCCTAC
J4-IB-88	CAGTAACCGACttACGGTCCGCAGGTCAGTATCA

J4-IB-89	CGGGCAGAGAGttCTATCATTCCGTGCTCCTAGC
J4-IB-90	GTCTTTACAGAttTGAGGGAAGCACCAATATAGC
J4-IB-91	CGCGGCTAACCttCTACTTGATATTCGGATAGAT
J4-IB-92	TCCGGCTCGAAttGTTGTACCATTGTGCCCGCCA
J4-IB-93	GTAGCTAGGTGttACATCGTTTCAAGCGTTAACC
J4-IB-94	ATCAGATTGAAttGGGCAAAGACCATGGTGATTC
J4-IB-95	TATATTCCTGCttCACGTGGACGATACGATAGCC
J4-IB-96	TTAAAGCCCATttGCAGGTGTTAACCACAACATC
J4-IB-97	ATACCACACCAttCGTGCTAGCCACTGACCAGCT
J4-IB-98	CTTGGTGACGGttGATCTGTTAACGACTCCAGCG
J4-IB-99	CTATTCAGCGTttTAGTTCGTGACtttttttttt
J4-IB-100	GTCGCTGCAAAttTTGTGTTGTATAATATGTCAG
J4-IB-101	TCCCGTTGTCGttTCGGCATGCTATCCCTCGAAG
J4-IB-102	TCAGATGCCGAttTGATGGGTACGGTGCTCTAGC
J4-IB-103	GCATGGCCCTCttTACCGCCTTCGGAACAGTGGT
J4-IB-104	AATTCTCTGACTAGGCACAGCTAACACCACTGTTC
J4-IB-105	CGAAGGCGGTATGATCTGGTCGGAGTAC
J4-IB-106	AGTTGCTGGTGttGAGGGCCATGCTGATACTGAC
J4-IB-107	CTGCGGACCGTttCTCTCTGCCCGGCTAGAGCAC
J4-IB-108	CGTACCCATCAttTCATCCACTCCTTGCTTAATG
J4-IB-109	ATCTTACAAGTttTCGGCATCTGAATCTATCCGA
J4-IB-110	ATATCAAGTAGttTTCGAGCCGGACTTCGAGGGA
J4-IB-111	TAGCATGCCGAttATTTATAGGGCGCCTCTGATG
J4-IB-112	CGCCCTACATAttCGACAACGGGAGAATCACCAT
J4-IB-113	GGTCTTTGCCCttGCAGGAATATACTGACATATT
J4-IB-114	ATACAACACAAttTGCTGTGCCCTCAAAGGTATC
J4-IB-115	GCATATAGCGGttTTTGCAGCGACAGCTGGTCAG
J4-IB-116	TGGCTAGCACGttCCGTCACCAAGtttttttttt
J4-IB-117	AACCCTGCTATttTAATTGCGCAGTGTTTAGGAT
J4-IB-118	ACCTGCGGATAttTCAAGAGGGCTTTGAGCTACC
J4-IB-119	GTTCGATGTCTttAGTTCGAACAGTTGGAAGGTC
J4-IB-120	CTCCGACCAGATCACACCAGCAACTGACCTTCCAA
J4-IB-121	CTGTTCGAACTCGGTGCCCTGTAACATC
J4-IB-122	CCTGTCTAGCAttAGACATCGAACCATTAAGCAA
J4-IB-123	GGAGTGGATGAttACTTGTAAGATGGTAGCTCAA
J4-IB-124	AGCCCTCTTGAttCTGAACATGGCCCTTCGTCCG
J4-IB-125	TACTACAAATAttTATCCGCAGGTCATCAGAGGC
J4-IB-126	GCCCTATAAATttTATGTAGGGCGATCCTAAACA
J4-IB-127	CTGCGCAATTAttCCCTATATGCATCTAGTAAGT
J4-IB-128	CATCCATGATGttATAGCAGGGTTGATACCTTTG
J4-IB-129	AGGGCACAGCAttCCGCTATATGCtttttttttt
J4-IB-130	TCCGACGTTACttATGGTCCCGCGGGCGGCACGT
J4-IB-131	CGCGCGACATAttATTCGGTATCTGTACTCAAAG
J4-IB-132	GTTACAGGGCACCGTGCTAGACAGGCTTTGAGTAC
J4-IB-133	AGATACCGAATCTCTGGTTGAACATTCA

J4-IB-134	CGTTGAAGTTAttTATGTCGCGCGGACGAAGG
J4-IB-135	GCCATGTTCAGttTATTTGTAGTAACGTGCCGCC
J4-IB-136	CGCGGGACCATttGCAGGACTGGACATGTAGCGA
J4-IB-137	CCTACCGGGAAttGTAACGTCGGAACTTACTAGA
J4-IB-138	TGCATATAGGGttCATCATGGATGtttttttttt
J4-IB-139	AGGGCTTTATTttCGTAAATAGAGTGGACCTGTA
J4-IB-140	ATGTTCAACCAGAGTAACTTCAACGTACAGGTCCA
J4-IB-141	CTCTATTTACGTGAAGCGATGCATGACT
J4-IB-142	GTAACTAATAGttAATAAAGCCCTTCGCTACATG
J4-IB-143	TCCAGTCCTGCttTTCCCGGTAGGtttttttttt
J4-IB-144	CATGCATCGCTTCACTATTAGTTACGAGTATAATT
